# NuCLS: A scalable crowdsourcing approach and dataset for nucleus classification and segmentation in breast cancer

**DOI:** 10.1093/gigascience/giac037

**Published:** 2022-05-17

**Authors:** Mohamed Amgad, Lamees A Atteya, Hagar Hussein, Kareem Hosny Mohammed, Ehab Hafiz, Maha A T Elsebaie, Ahmed M Alhusseiny, Mohamed Atef AlMoslemany, Abdelmagid M Elmatboly, Philip A Pappalardo, Rokia Adel Sakr, Pooya Mobadersany, Ahmad Rachid, Anas M Saad, Ahmad M Alkashash, Inas A Ruhban, Anas Alrefai, Nada M Elgazar, Ali Abdulkarim, Abo-Alela Farag, Amira Etman, Ahmed G Elsaeed, Yahya Alagha, Yomna A Amer, Ahmed M Raslan, Menatalla K Nadim, Mai A T Elsebaie, Ahmed Ayad, Liza E Hanna, Ahmed Gadallah, Mohamed Elkady, Bradley Drumheller, David Jaye, David Manthey, David A Gutman, Habiba Elfandy, Lee A D Cooper

**Affiliations:** Department of Pathology, Northwestern University, 750 N Lake Shore Dr., Chicago, IL 60611, USA; Cairo Health Care Administration, Egyptian Ministry of Health, 3 Magles El Shaab Street, Cairo, Postal code 222, Egypt; Department of Pathology, Nasser institute for research and treatment, 3 Magles El Shaab Street, Cairo, Postal code 222, Egypt; Department of Pathology and Laboratory Medicine, University of Pennsylvania, 3620 Hamilton Walk M163, Philadelphia, PA 19104, USA; Department of Clinical Laboratory Research, Theodor Bilharz Research Institute, 1 El-Nile Street, Imbaba Warrak El-Hadar, Giza, Postal code 12411, Egypt; Department of Medicine, Cook County Hospital, 1969 W Ogden Ave, Chicago, IL 60612, USA; Department of Pathology, Baystate Medical Center, University of Massachusetts, 759 Chestnut St, Springfield, MA 01199, USA; Faculty of Medicine, Menoufia University, Gamal Abd El-Nasir, Qism Shebeen El-Kom, Shibin el Kom, Menofia Governorate, Postal code: 32511, Egypt; Faculty of Medicine, Al-Azhar University, 15 Mohammed Abdou, El-Darb El-Ahmar, Cairo Governorate, Postal code 11651, Egypt; Consultant for The Center for Applied Proteomics and Molecular Medicine (CAPMM), George Mason University, 10920 George Mason Circle Institute for Advanced Biomedical Research Room 2008, MS1A9 Manassas, Virginia 20110, USA; Department of Pathology, National Liver Institute, Gamal Abd El-Nasir, Qism Shebeen El-Kom, Shibin el Kom, Menofia Governorate, Postal code: 32511, Egypt; Department of Pathology, Northwestern University, 750 N Lake Shore Dr., Chicago, IL 60611, USA; Faculty of Medicine, Ain Shams University, 38 Abbassia, Next to the Al-Nour Mosque, Cairo, Postal code: 1181, Egypt; Cleveland Clinic Foundation, 9500 Euclid Ave. Cleveland, Ohio 44195, USA; Department of Pathology, Indiana University, 635 Barnhill Drive Medical Science Building A-128 Indianapolis, IN 46202, USA; Faculty of Medicine, Damascus University, Damascus, Damaskus, PO Box 30621, Syria; Faculty of Medicine, Ain Shams University, 38 Abbassia, Next to the Al-Nour Mosque, Cairo, Postal code: 1181, Egypt; Faculty of Medicine, Mansoura University, 1 El Gomhouria St, Dakahlia Governorate 35516, Egypt; Faculty of Medicine, Cairo University, Kasr Al Ainy Hospitals, Kasr Al Ainy St., Cairo, Postal code: 11562, Egypt; Faculty of Medicine, Ain Shams University, 38 Abbassia, Next to the Al-Nour Mosque, Cairo, Postal code: 1181, Egypt; Faculty of Medicine, Menoufia University, Gamal Abd El-Nasir, Qism Shebeen El-Kom, Shibin el Kom, Menofia Governorate, Postal code: 32511, Egypt; Faculty of Medicine, Mansoura University, 1 El Gomhouria St, Dakahlia Governorate 35516, Egypt; Faculty of Medicine, Cairo University, Kasr Al Ainy Hospitals, Kasr Al Ainy St., Cairo, Postal code: 11562, Egypt; Faculty of Medicine, Menoufia University, Gamal Abd El-Nasir, Qism Shebeen El-Kom, Shibin el Kom, Menofia Governorate, Postal code: 32511, Egypt; Department of Anaesthesia and Critical Care, Menoufia University Hospital, Gamal Abd El-Nasir, Qism Shebeen El-Kom, Shibin el Kom, Menofia Governorate, Postal code: 32511, Egypt; Department of Clinical Pathology, Ain Shams University, 38 Abbassia, Next to the Al-Nour Mosque, Cairo, Postal code: 1181, Egypt; Faculty of Medicine, Ain Shams University, 38 Abbassia, Next to the Al-Nour Mosque, Cairo, Postal code: 1181, Egypt; Research Department, Oncology Consultants, 2130 W. Holcombe Blvd, 10th Floor, Houston, Texas 77030, USA; Department of Pathology, Nasser institute for research and treatment, 3 Magles El Shaab Street, Cairo, Postal code 222, Egypt; Faculty of Medicine, Ain Shams University, 38 Abbassia, Next to the Al-Nour Mosque, Cairo, Postal code: 1181, Egypt; Siparadigm Diagnostic Informatics, 25 Riverside Dr no. 2, Pine Brook, NJ 07058, USA; Department of Pathology and Laboratory Medicine, Emory University School of Medicine, 201 Dowman Dr, Atlanta, GA 30322, USA; Department of Pathology and Laboratory Medicine, Emory University School of Medicine, 201 Dowman Dr, Atlanta, GA 30322, USA; Kitware Inc., 1712 Route 9. Suite 300. Clifton Park, New York 12065, USA; Department of Neurology, Emory University School of Medicine, 201 Dowman Dr, Atlanta, GA 30322, USA; Department of Pathology, National Cancer Institute, Kasr Al Eini Street, Fom El Khalig, Cairo, Postal code: 11562, Egypt; Department of Pathology, Children’s Cancer Hospital Egypt (CCHE 57357), 1 Seket Al-Emam Street, El-Madbah El-Kadeem Yard, El-Saida Zenab, Cairo, Postal code: 11562, Egypt; Department of Pathology, Northwestern University, 750 N Lake Shore Dr., Chicago, IL 60611, USA; Lurie Cancer Center, Northwestern University, 675 N St Clair St Fl 21 Ste 100, Chicago, IL 60611, USA; Center for Computational Imaging and Signal Analytics, Northwestern University Feinberg School of Medicine, 750 N Lake Shore Dr., Chicago, IL 60611, USA

**Keywords:** crowdsourcing, deep learning, nucleus segmentation, nucleus classification, breast cancer

## Abstract

**Background:**

Deep learning enables accurate high-resolution mapping of cells and tissue structures that can serve as the foundation of interpretable machine-learning models for computational pathology. However, generating adequate labels for these structures is a critical barrier, given the time and effort required from pathologists.

**Results:**

This article describes a novel collaborative framework for engaging crowds of medical students and pathologists to produce quality labels for cell nuclei. We used this approach to produce the NuCLS dataset, containing >220,000 annotations of cell nuclei in breast cancers. This builds on prior work labeling tissue regions to produce an integrated tissue region- and cell-level annotation dataset for training that is the largest such resource for multi-scale analysis of breast cancer histology. This article presents data and analysis results for single and multi-rater annotations from both non-experts and pathologists. We present a novel workflow that uses algorithmic suggestions to collect accurate segmentation data without the need for laborious manual tracing of nuclei. Our results indicate that even noisy algorithmic suggestions do not adversely affect pathologist accuracy and can help non-experts improve annotation quality. We also present a new approach for inferring truth from multiple raters and show that non-experts can produce accurate annotations for visually distinctive classes.

**Conclusions:**

This study is the most extensive systematic exploration of the large-scale use of wisdom-of-the-crowd approaches to generate data for computational pathology applications.

## Background

### Motivation

Convolutional neural networks and other deep learning methods have been at the heart of recent advances in medicine (see Supplementary Table S1 for terminology) [1]. A key challenge in computational pathology is the scarcity of large-scale labeled datasets for model training and validation [2–4]. Specifically, there is a shortage of annotation data for delineating tissue regions and cellular structures in histopathology. This information is critical for training interpretable deep-learning models because they allow the detection of entities that are understood by pathologists and map to known diagnostic criteria [4–7]. These entities can then be used to construct higher-order relational graphs that encode complex spatial and hierarchical relationships within the tumor microenvironment, paving the way for the computationally driven discovery of histopathologic biomarkers and biological associations [4, 8–13]. Data shortage is often attributed to the domain expertise required to produce annotation labels, with pathologists spending years in residency and fellowship training [2, 14]. This problem is exacerbated by the time constraints of clinical practice and the repetitive nature of annotation work. Manual tracing of object boundaries is an incredibly demanding task, and there is a pressing need to obtain these data using facilitated or assisted annotation strategies [15]. By comparison, traditional annotation problems such as detecting people in natural images require almost no training and typically engage the general public [15]. Moreover, unique problems often require new annotation data, underscoring the need for scalable and reproducible annotation workflows [16].

We address these issues using an assisted annotation method that leverages the participation of non-pathologists (NPs), including medical students and graduates. Medical students typically have strong incentives to participate in annotation studies, with increased reliance on research participation in residency selection [17]. We describe adaptations to the data collection to improve scalability and reduce effort. This work focuses on nucleus classification, localization, and segmentation (NuCLS) in whole-slide scans of hematoxylin and eosin (H&E)-stained slides of breast carcinoma from 18 institutions from The Cancer Genome Atlas (TCGA). Our annotation pipeline enables low-effort collection of nucleus segmentation and classification data, paving the way for systematic discovery of histopathologic-genomic associations and morphological biomarkers of disease progression [4, 5, 8, 10, 11].

### Related work

There has been growing interest in addressing data scarcity in histopathology by either (i) scaling data generation or (ii) reducing reliance on manually labeled data using data synthesis techniques such as generative adversarial networks [18–25]. While there is a pressing need for both approaches, this work is meant to fit into the broad context of scalable assisted manual data generation when expert annotation is expensive or difficult. Crowdsourcing, the process of engaging a “crowd” of individuals to annotate data, is critical to solving this problem. There exists a large body of relevant work in crowdsourcing for medical image analysis [15, 26, 27]. Previously, we published a study and dataset using crowdsourcing of NPs for annotation of low-power regions in breast cancer [28]. Our approach was structured because we assigned different tasks depending on the level of expertise and leveraged collaborative annotation to obtain data that are large in scale and high in quality. Here, we significantly expand this idea by focusing on the challenging problems of nucleus classification, localization, and segmentation. This computer vision problem is a subject of significant interest in computational pathology [29–31].

While the public release of data is only 1 aspect of our study, it is essential to acknowledge related nucleus classification datasets. Some of these datasets can be used in conjunction with ours and include MoNuSAC, CoNSep, PanNuke, and Lizard [29, 30, 32–38]. Lizard, in particular, is a highly related dataset that was recently published after we released NuCLS but focuses on colon cancer instead [37]. Additionally, the US Food and Drug Administration is leading an ongoing study to collect regulatory-grade annotations of stromal tumor-infiltrating lymphocytes (sTILs) [39]. Unfortunately, with few exceptions, most public computational pathology datasets either are limited in scale, were generated through exhaustive annotation efforts by practicing pathologists, or do not disclose or discuss data generation [2, 26, 30, 40]. Additionally, to the best of our knowledge, most other works do not explore crowdsourcing as a data generation approach or systematically explore interrater agreement for experts vs non-experts.

A few studies are of particular relevance to this article. A study by Irshad et al. showed that non-experts, recruited through the Figure Eight platform, can produce accurate nucleus detections and segmentations in renal clear cell cancer but was limited to 10 whole-slide images [20]. Hou et al. explored the use of synthetic data to produce nuclear segmentations [41]. While a significant contribution, their work did not address classification, relied on qualitative slide-level evaluations of results, and did not explore how algorithmic bias affects data quality [22, 42]. The approach we used involves click-based approval of annotations generated by a deep-learning algorithm. This methodological aspect is not the central focus of this article; it is only one of many approaches for interactive segmentation and classification of nuclei explored in past studies such as HistomicsML and NuClick [22, 42].

### Our contributions

This work describes a scalable crowdsourcing approach that systematically engaged NPs and produced annotations for localization, segmentation, and classification of nuclei in breast cancer. Our workflow required minimal effort from pathologists and used algorithmic suggestions to scale the annotation process and obtain hybrid annotation datasets containing numerous segmentation boundaries without laborious manual tracing. We show that algorithmic suggestions can improve the accuracy of NP annotations and that NPs are reliable annotators of common cell types. In addition, we discuss a new constrained clustering method that we developed for reliable truth inference in multi-rater datasets. We also show how multi-rater data can ensure the quality of NP annotations or replace expert supervision in some contexts. Finally, we note that downstream deep-learning modeling using the NuCLS dataset is discussed in a related publication and is not the focus of this article [43].

## Data description

NuCLS is a large-scale multi-class dataset generated by engaging crowds of medical students and pathologists. NuCLS is sourced from the same images as the Breast Cancer Semantic Segmentation (BCSS) dataset [28]. Together, these datasets contain region- and cell-level annotations and constitute, to our knowledge, the most extensive resource for multi-scale analysis of breast cancer slides. We obtained a total of 222,396 nucleus annotations, including >125,000 single-rater annotations and 97,000 multi-rater annotations. A detailed description of the dataset creation protocol is presented in the Methods section.

## Analyses and Discussion

### Structured crowdsourcing enables scalable data collection

**Figure 1:**
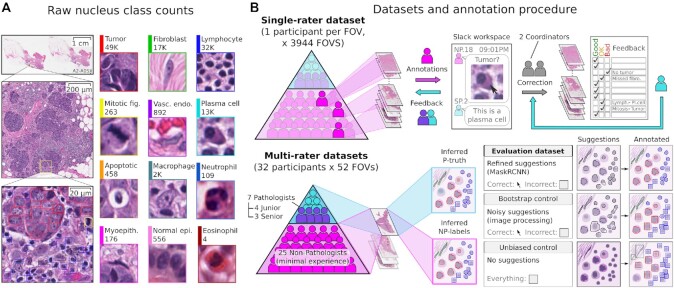
Dataset annotation and quality control procedure. A. Nucleus classes annotated. B. Annotation procedure and resulting datasets. Two approaches were used to obtain nucleus labels from non-pathologists (NPs). (Top) The first approach focused on breadth, collecting single-rater annotations over a large number of FOVs to obtain the majority of data in this study. NPs were given feedback on their annotations, and 2 study coordinators corrected and standardized all single-rater NP annotations on the basis of input from a senior pathologist. (Bottom) The second approach evaluated interrater reliability and agreement, obtaining annotations from multiple NPs for a smaller set of shared FOVs. Annotations were also obtained from pathologists for these FOVs to measure NP reliability. The procedure for inferring a single set of labels from multiple participants is described in Fig. 2. We distinguished between inferred non-pathologist labels (NP-labels) and inferred pathologist truth (P-truth) for clarity. Three multi-rater datasets were obtained: an Evaluation dataset, which is the primary multi-rater dataset, as well as Bootstrap and Unbiased experimental controls to measure the value of algorithmic suggestions. In all datasets except the Unbiased control, participants were shown algorithmic suggestions for nucleus boundaries and classes. They were directed to click nuclei with correct boundary suggestions and annotate other nuclei with bounding boxes. The pipeline to obtain algorithmic suggestions consisted of 2 steps: (i) Using image processing to obtain bootstrapped suggestions (Bootstrap control); (ii) Training a Mask R-CNN deep-learning model to refine the bootstrapped suggestions (single-rater and Evaluation datasets).

Pathologist time is limited and expensive, and relying solely on pathologists for generating annotations can hinder the development of state-of-the-art models based on convolutional neural networks. In this study, we show that NPs can perform most of the time-consuming annotation tasks and that pathologist involvement can be limited to low-effort tasks that include:

- Training NPs and answering their questions (Fig. 1) [44].
- Qualitative scoring of NP annotations (Supplementary Fig. S1).
- Low-power annotation of histologic regions (Supplementary Fig. S2) [28].

We used a web-based annotation platform called HistomicsUI for annotation, feedback, and quality review [45]. HistomicsUI provides a user interface with annotation tools and an API for programmatic querying and manipulating the centralized annotation database. The NuCLS dataset includes annotations from 32 NPs and 7 pathologists in the USA, Egypt, Syria, Australia, and the Maldives. We obtained 128,000 nucleus annotations from 3,944 fields of view (FOVs) and 125 patients with triple-negative breast cancer. The annotations included bounding box placement, classification, and, for a sizable fraction of nuclei, segmentation boundaries. Half of these annotations underwent quality control correction based on feedback by a practicing pathologist.

**Figure 2:**
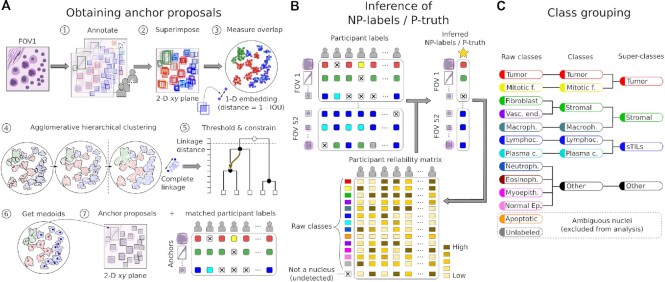
Inference from multi-rater datasets. The purpose of this step was to infer the nucleus locations and classifications from multi-rater data. A. The first step involved agglomerative hierarchical clustering of bounding boxes using intersection-over-union (IOU) as a similarity measure. We imposed a constraint during clustering that prevents merging annotations where a single participant has annotated overlapping nuclei. Participant intention was preserved by demoting annotations from the same participant to the next node (Step 5, arrow). After clustering was complete, a threshold IOU value was used to obtain the final clusters (Step 5, black nodes). Within each cluster, the medoid bounding box was chosen as an anchor proposal. The result was a set of anchors with corresponding clustered annotations. When a participant did not match to an anchor, it was considered a conscious decision not to annotate a nucleus at that location. B. Once anchors were obtained, an expectation-maximization procedure was used to estimate (i) which anchors represent actual nuclei and (ii) which classes to assign these anchors. The expectation-maximization procedure estimates and accounts for the reliability of each participant for each classification. Expectation-maximization was performed separately for NPs and pathologists. C. Grouping of nucleus classes. Consistent with standard practice in object detection, nuclei were grouped, on the basis of clinical reasoning, into 5 classes and 3 super-classes.

Additionally, we obtained 3 multi-rater datasets containing 97,300 annotations, where the same FOV was annotated by multiple participants (Figs 1B and 2). The collection of multi-rater data enables quantitative evaluation of NP reliability, interrater variability, and the effect of algorithmic suggestions on NP accuracy. Multi-rater annotations were *not* corrected by pathologists and enabled an unbiased assessment of NP performance. Pathologist annotations were also collected for a limited set of multi-rater FOVs to evaluate NP accuracy.

### NPs can reliably classify common cell types

**Figure 3:**
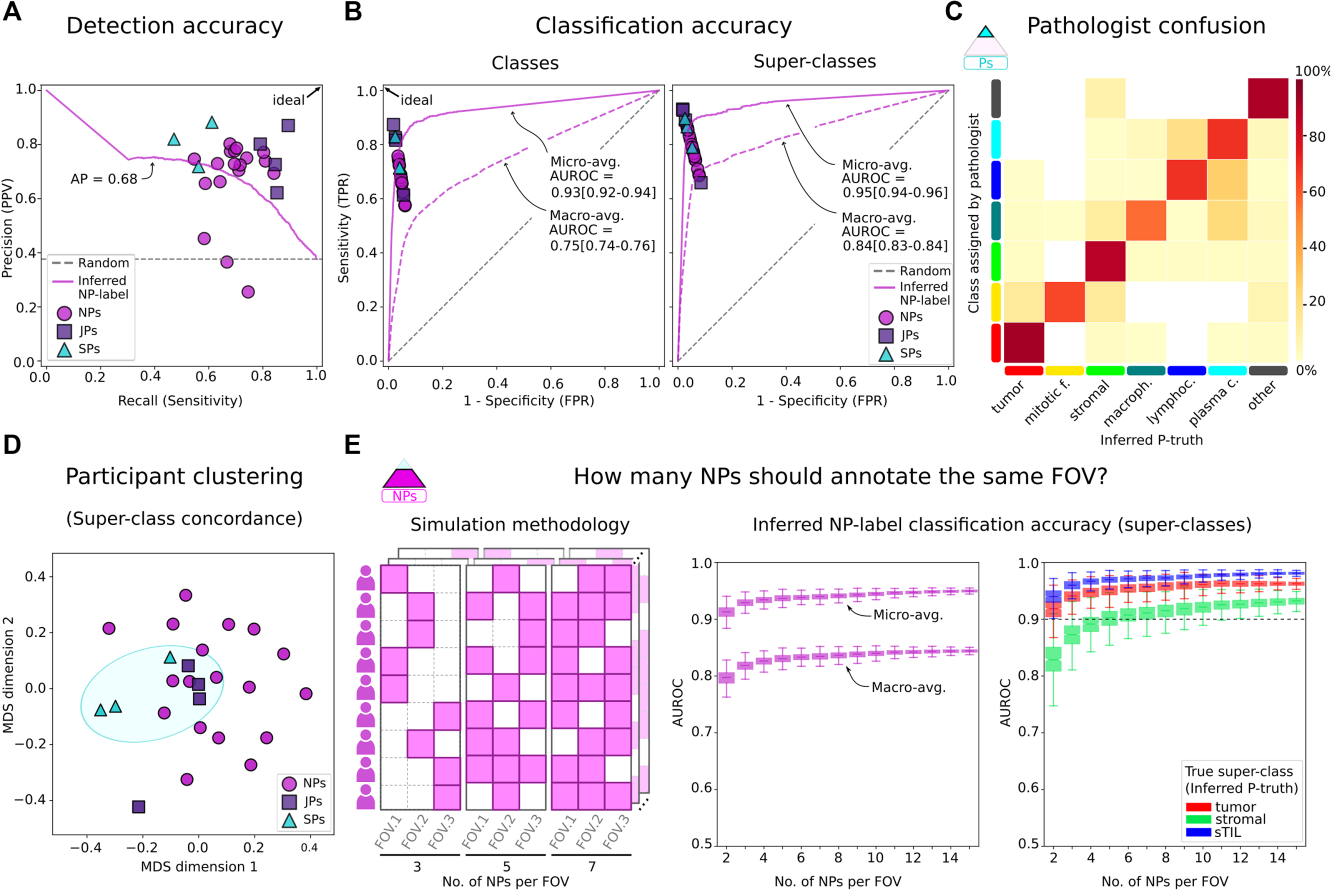
Accuracy of participant annotations. A. Detection precision-recall comparing annotations to inferred P-truth. Junior pathologists tend to have similar precision but higher recall than senior pathologists, possibly reflecting the time constraints of pathologists. PPV: positive predictive value. B. Classification ROC for classes and super-classes. The overall classification accuracy of inferred NP-labels was high. However, class-balanced accuracy (macro-average) is notably lower because NPs are less reliable annotators of uncommon classes. FPR: false-positive rate. C. Confusion between pathologist annotations and inferred P-truth. D. Multidimensional scaling (MDS) analysis of interrater classification agreement. Some clustering by participant experience (blue ellipse) highlights the importance of modeling reliability during label inference. E. A simulation was used to measure how redundancy affects the classification accuracy of inferred NP-labels. While keeping the total number of NPs constant, we randomly kept annotations for a variable number of NPs per FOV. Accuracy in these simulations was class-dependent, with stromal nuclei requiring more redundancy for accurate inference. Each simulation is represented by one notched box plot, where notches correspond to the bootstrapped 95% interval around the median, and the whiskers extend for 1.5x the interquartile range.

The detection accuracy of NPs was moderately high (average precision = 0.68) and was similar to the detection accuracy of pathologists. Classification accuracy of NPs, on the other hand, was only high for common nucleus classes (micro-average area under receiver-operator characteristic curve [AUROC] = 0.93 [95% CI, 0.92–0.94] vs macro-average AUROC = 0.75 [95% CI, 0.74–0.76]) and was higher when grouping by super-class (Fig. 3, Supplementary Fig. S3). We reported the same phenomenon in our previous work on crowdsourcing annotation of tissue regions [28]. In addition, we observed moderate clustering by participant experience (Fig. 3D) and variability in classification accuracy among NPs (Matthews correlation coefficient [MCC] = 60.7–84.2). This observation motivated our quality control procedures. Study coordinators manually corrected missing or misclassified cells for the single-rater dataset, and practicing pathologists supervised and approved annotations. For the multi-rater datasets, we inferred a singular label from pathologists (P-truth) and NPs (NP-label) using an expectation-maximization framework that estimates reliability values for each participant [46, 47].

When pathologist supervision is not an option, multi-rater datasets need to have annotations from a sufficient number of NPs to infer reliable data. We used the annotations we obtained to perform simulations to estimate the accuracy of inferred NP-labels with fewer numbers of participating NPs (Fig. 3E). The inferred NP-label accuracy increased up to 6 NPs per FOV, after which there were diminishing returns. Our simulations also showed that stromal nuclei require more NPs per FOV than tumor nuclei or sTILs.

### Minimal-effort collection of nucleus segmentation data

Many nucleus detection and segmentation algorithms were developed using conventional image analysis methods before the widespread adoption of convolutional neural networks. These algorithms have little or no dependence on annotations, and while they may not be as accurate as convolutional neural networks, they can correctly segment a significant fraction of nuclei. We used simple nucleus segmentation heuristics, combined with low-power region annotations from the BCSS dataset, to obtain bootstrapped annotation suggestions for nuclei (Supplementary Fig. S2) [28]. The suggestions were refined using a well-known deep-learning model (Mask R-CNN) as a function approximator trained on the bootstrapped suggestions. This procedure allowed poor-quality bootstrapped suggestions in 1 FOV to be smoothed by better suggestions in other FOVs (Supplementary Fig. S4, Supplementary Table S2) and is analogous to fitting a regression line to noisy data [18, 48]. This model was applied to the FOVs to generate refined suggestions shown to participants when annotating the single-rater dataset and the Evaluation dataset (the primary multi-rater dataset) [44]. Two additional multi-rater datasets were obtained as controls:

- Bootstrap control: participants were shown unrefined bootstrapped suggestions.
- Unbiased control: participants were not shown any suggestions. This dataset was the first multi-rater dataset to be annotated.

**Figure 4:**
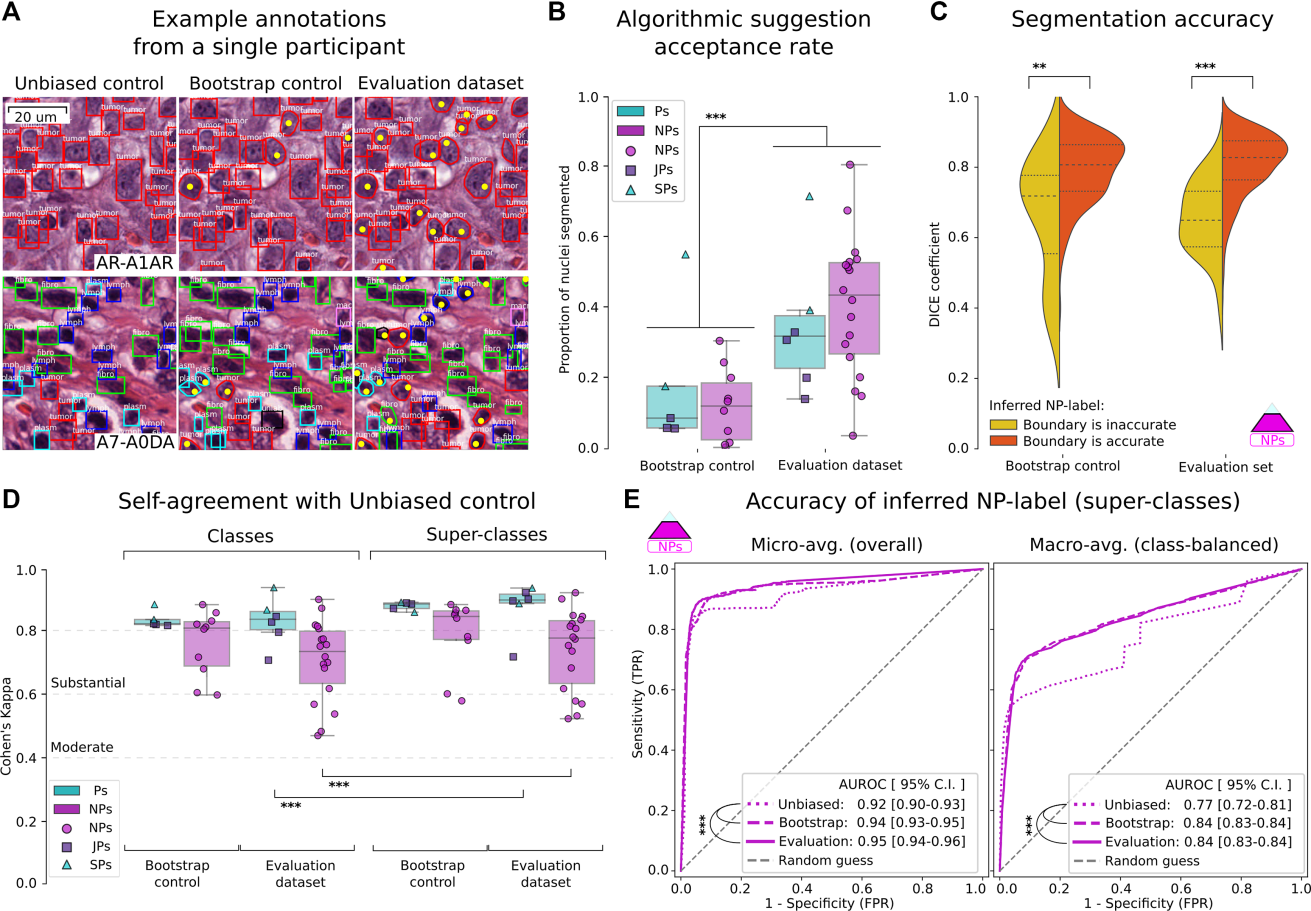
Effect of algorithmic suggestions on annotation abundance and accuracy. We compared annotations from the Evaluation dataset and controls to measure the effect of suggestions and Mask R-CNN refinement on the acquisition of nucleus segmentation data and the accuracy of annotations. A. Example annotations from a single participant. Algorithmic suggestions allow the collection of accurate nucleus segmentations without added effort. Yellow points indicate clicks to approve suggestions. B. The number of segmented nuclei clicked is significantly higher for the Evaluation dataset than for the Bootstrap control, indicating that refinement improves suggestion quality. C. Accuracy of algorithmic segmentation suggestions. The comparison is made against a limited set of manually traced segmentation boundaries obtained from 1 senior pathologist (SP). Suggestions that were determined to be correct by the expectation-maximization procedure had significantly more accurate segmentation boundaries. D. Self-agreement for annotations in the presence or absence of algorithmic suggestions. The agreement is substantial for non-pathologist (NP) and pathologist (P) groups, indicating that algorithmic suggestions do not affect classification decisions adversely. Pathologists have higher self-agreement and are less impressionable than NPs. E. ROC curves for the classification accuracy of inferred NP-label, using inferred P-truth as our reference. **P < 0.01; ***P < 0.001.

Accurate suggestions can be confirmed during annotation with a single click, reducing effort and providing valuable nucleus boundaries that can aid the development of segmentation models. Participants can annotate nuclei that have poor suggestions using bounding boxes. Bounding box annotation requires more effort than clicking a suggestion but less effort than the manual tracing of nuclear boundaries [15]. We obtained a substantial proportion of nucleus boundaries through clicks: 41.7 ± 17.3% for the Evaluation dataset and 36.6% for the single-rater dataset (Fig. 4, Supplementary Fig. S5). The resultant hybrid dataset contained a mixture of bounding boxes and accurate segmentation boundaries (Evaluation dataset Dice coefficient = 85.0 ± 5.9). We argue that it is easier to handle hybrid datasets at the level of algorithm development than to have participants trace missing boundaries or correct imprecise ones. We evaluate the bias of using these suggestions in the following section.

### Algorithmic suggestions improve classification accuracy

There was value in providing the participants with suggestions for nuclear class, which included suggestions directly inherited from BCSS region annotations, as well as high-power refined suggestions produced by Mask R-CNN (Fig. 4). Pathologists had substantial self-agreement when annotating FOVs with or without refined suggestions (κ = 87.4 ± 7.9). NPs also had high self-agreement but were more impressionable when presented with suggestions (κ = 74.0 ± 12.6). This was, however, associated with a reduction in bias in their annotations; refined suggestions improved the classification accuracy of inferred NP-labels (AUROC = 0.95 [95% CI, 0.94–0.96] vs 0.92 [95% CI, 0.90–0.93], *P* < 0.001). This observation is consistent with Marzahl et al., who reported similar findings in a crowdsourcing study using bovine cytology slides [27].

Region-based class suggestions for nuclei were, overall, more concordant with the corrected single-rater annotations compared to Mask R-CNN refined (high-power) nucleus suggestions (MCC = 67.6 vs 52.7) (Supplementary Fig. S4, Supplementary Table S2). Nonetheless, high-power nucleus suggestions were more accurate for 24.8% of FOVs and had a higher recall for sTILs (96.8 vs 76.6) [4, 11]. This result makes sense because stromal regions often contain scattered sTILs and a region-based approach to labeling would incorrectly mark these as stromal nuclei (e.g., see Supplementary Fig. S6) [28, 49]. Hence, the value of low- and high-power classification suggestions is context-dependent.

### Exploring nucleus detection and classification trade-offs

**Figure 5:**
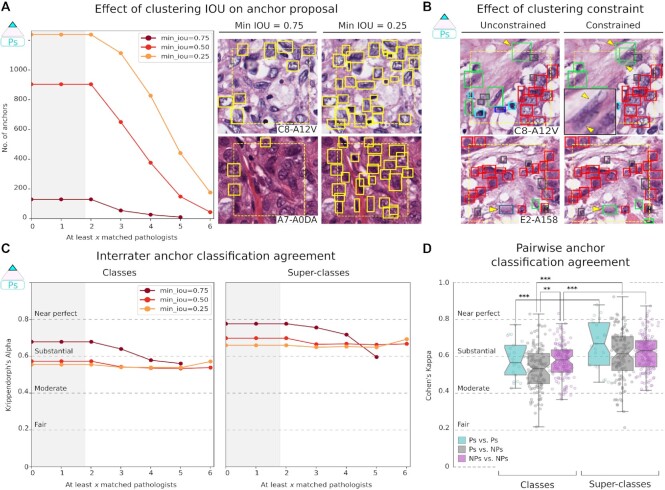
Effect of clustering on detection and interrater agreement. A. Stricter IOU thresholds reduce the number of anchor proposals generated by clustering but increase agreement. A threshold of 0.25 provides more anchor proposals with negligible difference in agreement from the 0.5 threshold. The shaded region indicates that by design, there are no anchor proposals with <2 clustered annotations. B. The clustering constraint prevents annotations from the same participant from being assigned to the same anchor, preserving participant intention when annotating overlapping nuclei. This results in better detection of overlapping nuclei during clustering (upper panel) and also affects the inferred P-truth for anchors (bottom panel). C. Interrater classification agreement among pathologists for tested clustering thresholds. D. Pairwise interrater classification agreement (Cohen κ) at 0.25 IOU threshold. **P < 0.01; ***P < 0.001.

Naturally, there is some variability in the judgments made by participants about nuclear locations and classes and the accuracy of suggested boundaries. We study the process of inferring a single truth from multi-rater datasets and discuss the effect of various parameters. There is a trade-off between the number of nucleus anchor proposals and interrater agreement (Fig. 5). The clustering IOU threshold that defines the minimum acceptable overlap between any 2 annotations substantially affected the number of anchor proposals. We found that an IOU threshold of 0.25 detects most nuclei with adequate pathologist classification agreement (1,238 nuclei, α = 55.5). We imposed a constraint to prevent annotations from the same participant from mapping to the same cluster—this improved detection of touching nuclei when the number of pathologists was limited (Fig. 5B).

Nucleus detection was a more significant source of discordance among participants than nucleus classification (Fig. 3, Supplementary Figs S7 and S8). Some nucleus classes were easier to detect than others. sTILs were the easiest to detect, likely owing to their hyperchromicity and tendency to aggregate; 53.3% of sTILs were detected by ≥16 NPs (Supplementary Fig. S9). Fibroblasts were demonstrably harder to detect (only 21.4% were detected by ≥16 NPs), likely because of their relative sparsity and lighter nuclear staining. Lymphocytes and plasma cells, which often co-aggregate in lymphoplasmacytic clusters, were a source of interrater discordance for pathologists and NPs [4, 50]. This discordance may stem from variable degrees of reliance on low-power vs high-power morphologic features. Interrater agreement for nuclear classification was high and significantly improved when classes were grouped into clinically salient super-classes (α = 66.1 [pathologists] and 60.3 [NPs]; Fig. 5).

## Methods

### Data sources

The scanned diagnostic slides we used were generated by the TCGA Research Network (https://www.cancer.gov/tcga). They were obtained from 125 patients with breast cancer (1 slide per patient). Specifically, we chose to focus on all carcinoma of unspecified type cases that were triple-negative. The designation of histologic and genomic subtypes was based on public TCGA clinical records [28]. All slides were stained with H&E and were formalin-fixed and paraffin-embedded. The scanned slides were accessed using the Digital Slide Archive repository [45].

Region annotations were obtained from BCSS, a previous crowdsourcing study that we conducted [28]. Regions of interest (ROIs),  1 mm^2^ in size, were assigned to participants by difficulty level. All region annotations were corrected and approved by a practicing pathologist. These region annotations were used to obtain nucleus class suggestions as described below. Region classes included tumor, stroma, lymphocytic infiltrate, plasmacytic infiltrate, necrosis/debris, and other uncommon regions.

### Algorithmic suggestions

The process for generating algorithmic suggestions is summarized in Supplementary Fig. S2 and involves the following steps:

#### Heuristic nucleus segmentation

We used simple image processing heuristics to obtain noisy nucleus segmentations [31]. Images were analyzed at scan magnification (40×) with the following steps: (i) H&E stain unmixing using the Macenko method [51]. (ii) Gaussian smoothing followed by global Otsu thresholding to identify foreground nuclei pixels [52]. This step was done for each region class separately to increase robustness. We used a variance of 2 pixels for lymphocyte-rich regions and 5 pixels for other regions. (iii) Connected-component analysis split the nuclei pixel mask using 8-connectivity and a 3 × 3 structuring element [53]. (iv) We computed the Euclidean distance from every nucleus pixel to the nearest background pixel and found the peak local maxima using a minimum distance of 10 [54]. (v) A watershed segmentation algorithm split the connected components from Step 3 into individual nuclei using the local maxima from Step 4 as markers [55, 56]. (vi) Any object <300 pixels in area was removed.

#### Bootstrapping noisy training data

Region annotations were used to assign a noisy class to each segmented nucleus. This decision was based on the observation that although tissue regions usually contain multiple cell types, there is often a single predominant cell type: tumor regions/tumor cells, stromal regions/fibroblasts, lymphocytic infiltrate/lymphocytes, plasmacytic infiltrate/plasma cells, other regions/other cells. One exception to this direct mapping is stromal regions, which contain a large number of sTILs in addition to fibroblasts. Within stromal regions, a nucleus was considered a fibroblast if it had a spindle-like shape with an aspect ratio between 0.4 and 0.55 and circularity between 0.7 and 0.8.

#### Mask R-CNN refinement of bootstrapped suggestions

A Mask R-CNN model with a Resnet50 backbone was used as a function approximator to refine the bootstrapped nucleus suggestions. This model was trained using randomly cropped 128 × 128 tiles where the number of nuclei was limited to 30. Supplementary Table S3 includes other hyperparameters.

#### FOV sampling procedure

ROI locations were carried over from the BCSS dataset. ROIs were manually selected by a physician (M.A.), who served as a study coordinator for both the BCSS and NuCLS projects, and approved by a senior pathologist (H.E.). These ROIs were then tiled into non-overlapping potential FOVs, which were automatically selected for inclusion in our study on the basis of predefined stratified sampling criteria. A total of 16.7% of FOVs were sampled such that the majority of refined suggestions were a single class, e.g., almost all suggestions are tumor. In addition, 16.7% were sampled to favor FOVs with 2 almost equally represented classes, e.g., many tumor and fibroblast suggestions. Finally, 16.7% of FOVs were sampled to favor discordance between the bootstrapped suggestions and Mask R-CNN–refined suggestions, e.g., a stromal region with sTILs. The remaining 50% of FOVs were randomly sampled from the following pool, with the intent of favoring the annotation of difficult nuclei: (i) the bottom 5% of FOVs containing high numbers of nuclei with low Mask R-CNN confidence; (ii) and the top 5% of FOVs containing extreme size detections, presumably clumped nuclei.

### Annotation procedure and data management

The annotation protocol used is provided in the Supplementary Material. We asked the participants to annotate the single-rater dataset first because this also acted as their de facto training. Participants were blinded to the multi-rater dataset name to avoid biasing them. The Unbiased control was annotated first for the same reason. A summary of the data management procedure is provided below.

#### HistomicsUI

We used the Digital Slide Archive, a web-based data management tool, to assign slides and annotation tasks (digitalslidearchive.github.io) [45]. HistomicsUI, the associated annotation interface, was used for creating, correcting, and reviewing annotations. Using a centralized set-up avoids participants installing software and simplifies the dissemination of images, control over view/edit permissions, monitoring progress, and collecting results. The annotation process is illustrated in this video: https://www.youtube.com/watch?v=HTvLMyKYyGs. The process of pathologist review of annotations is illustrated in Supplementary Fig. S1.

#### HistomicsTK Application Programming Interface

The HistomicsTK Restful API was used to manage data, users, and annotations programmatically. This includes uploading algorithmic suggestions, downloading participant annotations, and scalable correction of systematic annotation errors where appropriate.

### Obtaining labels from multi-rater datasets

#### Obtaining anchor proposals

We implemented a constrained agglomerative hierarchical clustering process to obtain anchor proposals (Fig. 2A). The algorithm is summarized in Supplementary Fig. S10. In order to have a single frame of reference for comparison, annotations from all participants and for all multi-rater datasets were clustered. After clustering, we used 2 rules to decide which anchor proposals corresponded to actual nuclei (for each multi-rater dataset independently): (i) ≥2 pathologists must detect a nucleus and (ii) the inferred P-truth must concur that the anchor is a nucleus.

#### Inference of NP-labels and P-truth

We used the expectation-maximization framework described by Dawid and Skene [46, 47, 57]. Each participant was assigned an initial quality score of 0.7, and 70 expectation-maximization iterations were performed. As illustrated in Fig. 2B, undetected was considered a nucleus class for P-truth/NP-label inference. The same process was used to infer whether the boundary of an algorithmic suggestion was accurate. In effect, the segmentation accuracy was modeled as a binary variable (clicked vs not clicked), and the expectation-maximization procedure was applied to infer its value.

### Class grouping

We defined 2 levels of grouping for nuclei classes as illustrated in Fig. 2C. This was done for both the single-rater and multi-rater dataset annotations. Aggregate expectation-maximization probability was calculated by summing probabilities across subsets.

### Participant agreement

Overall interrater agreement was measured using the Krippendorff α-statistic, implemented in Python by Santiago Castro and Thomas Grill [58–60]. This statistic was chosen because of its ability to handle missing values [61]. Pairwise interrater agreement was measured using the Cohen κ-statistic [62]. Likewise, self-agreement was measured using Cohen κ. All of these measures range from −1 (perfect disagreement) to +1 (perfect agreement). A κ (or α) value of zero represents agreement that is expected by random chance. We used thresholds set by Fleiss for defining slight, fair, moderate, substantial, and near-perfect agreement [61].

### Annotation redundancy simulations

We performed simulations to measure the effect of the number of NPs assigned to each FOV on the accuracy of NP-label inference (Fig. 3E). We kept the total number of NPs constant at 18 and randomly removed annotations to obtain a desired number of NPs per FOV. No constraints were placed on how many FOVs any single NP had. This simulated the realistic scenario where participants can annotate as many FOVs as they want, and our decision-making focuses on FOV assignment. For each random realization, we calculated the inferred NP-labels using expectation-maximization and measured accuracy against the static P-truth. This process was repeated for 1,000 random realizations per configuration.

### Software

Data management, machine learning models, and plotting were all implemented using Python 3+. Pytorch and Tensorflow libraries were used for various deep-learning experiments. Scikit-learn, Scikit-image, OpenCV, HistomicsTK, Scipy, Numpy, and Pandas libraries were used for matrix and image-processing operations. Openslide library and HistomicsTK API were used for interaction with whole-slide images.

### Statistical tests

The Mann-Whitney *U* test was used for unpaired comparisons. The Wilcoxon signed-rank test was used for paired comparisons. Confidence bounds for the AUROC values were obtained by bootstrap sampling with replacement using 1,000 trials [63, 64]. AUROC values are presented with 95% CI.

## Conclusion

In summary, we have described a scalable crowdsourcing approach that benefits from the participation of NPs to reduce pathologist effort and enables minimal-effort collection of segmentation boundaries. We systematically examined aspects related to the interrater agreement and truth inference. There are important limitations and opportunities to improve on our work. Our results suggest that the participation of NPs can help address the scarcity of pathologists’ availability, especially for repetitive annotation tasks. This benefit, however, is restricted to annotating predominant and visually distinctive patterns. Naturally, pathologist input—and possibly full-scale annotation effort—would be needed to supplement uncommon and challenging classes that require greater expertise. Some nuclear classes may be challenging to annotate in H&E-stained slides reliably and would be subject to considerable interrater variability even among practicing pathologists. In these settings, and where resources allow, immunohistochemical stains may be used as a more objective form of ground truth [65].

We chose to engage medical students and graduates with the presumption that familiarity with basic histology would help in acquiring higher-quality data. Whether this presumption was warranted or whether it was possible to engage a broader pool of participants was not investigated. On a related note, while we observed differences based on pathologist expertise, this was not our focus. We expect to address related questions such as the value of fellowship specialization in future work. Also, we did not measure the time it took participants to create annotations; we relied on the safe assumption that certain annotation types evidently take less time and effort than others.

Another limitation is that the initial bootstrapped nuclear boundaries were generated using classical image-processing methods, which tend to underperform where nuclei are highly clumped/touching or have very faint staining. This theoretically introduces some bias in our dataset, with an overrepresentation of simpler nuclear boundaries. Future work could investigate the use of transfer learning or unsupervised convolutional neural network approaches to generate more accurate algorithmic suggestions. Similarly, we used Mask R-CNN as a function approximator to refine our algorithmic suggestions. Future research can explore other deep-learning architectures that may improve refinement and result in better algorithmic suggestions.

We focused our annotation efforts on nucleus detection, as opposed to whole cells. Nuclei have distinct staining (H&E) and boundaries, potentially reducing the interrater variability associated with the detection of cell boundaries. Finally, we would point out that dataset curation is context-dependent and likely differs depending on the problem. Nevertheless, we trust that most of our conclusions have broad implications for other histopathology annotation efforts.

## Availability of Source Code and Requirements

Project name: NuCLS

Project home page: github.com/PathologyDataScience/NuCLS

Operating system: Platform independent

Programming language: Python

Other requirements: We used the tensorflow implementation by Matterport Inc. to train the Mask R-CNN tensorflow model used for generating the algorithmic suggestions, along with a set of scripts available on GitHub at: https://github.com/PathologyDataScience/Mask_RCNN/. We used the Digital Slide Archive for whole-slide image and data management (available at: https://github.com/DigitalSlideArchive/digital_slide_archive) and its associated annotation user interface HistomicsUI (available at: https://github.com/DigitalSlideArchive/HistomicsUI), as well as the annotation and image-processing library HistomicsTK (available at: https://github.com/DigitalSlideArchive/HistomicsTK).

License: The NuCLS codebase is licensed with a CC0 1.0 license (dataset) and the MIT license.

Restrictions to use by non-academics: Both the CC0 1.0 license (dataset) and the MIT license (codebase) allow for non-commercial use. License terms can be reviewed for details.

Registration: RRID:SCR_021888; Biotools ID: nucls

## Data Availability

The NuCLS dataset is available at the NuCLS website. The BCSS dataset, which helped contribute to the algorithmic suggestions, is available for download from: https://github.com/PathologyDataScience/BCSS and can be viewed at a demo instance of the Digital Slide Archive at: https://demo.kitware.com/histomicstk/histomicstk#?image=5bbdee62e629140048d01b0d. Both the BCSS and NuCLS datasets are available under a CC0 1.0 license. Snapshots of our code and other data further supporting this work are openly available in the GigaScience repository, GigaDB [66].

## Additional Files

**Supplementary Table S1**: Definitions and abbreviations used. A white paper from the Digital Pathology Association can be consulted for an expanded list of relevant concepts [2].

**Supplementary Table S2**: Accuracy of algorithmic suggestions. The accuracy is measured against the corrected single-rater dataset. Mask R-CNN refinement of the bootstrapped algorithmic suggestions results in better detection suggestions. Low-power region-based classification was more accurate than Mask R-CNN–derived classes. Note, however, that this was FOV-dependent, and there were some FOVs in which the Mask R-CNN prediction was better than relying on low-power regions for classification

**Supplementary Table S3**: Hyperparameters used for Mask R-CNN model training.

**Supplementary Figure S1**: Use of review galleries for scalable review of single-rater annotations. Single-rater annotations were corrected by 2 study coordinators, in consultation with a senior pathologist. The pathologist was provided with a mosaic review gallery showing a bird’s eye view of each FOV, with and without annotations, and at high and low power. The pathologist was asked to assign a per-FOV quality assessment. If the pathologist wanted further context, they could click on the FOV and pan around the full whole-slide image. They were also able to provide brief comments to be addressed by the coordinators, e.g., “change all to tumor.” A demo is provided at the following video: https://youtu.be/Plh39obBg_0.

**Supplementary Figure S2**: Process for obtaining algorithmic suggestions for scalable assisted annotation. Nucleus segmentation boundaries were derived using image-processing heuristics at a high magnification. Low-power region annotations from the BCSS dataset, approved by a practicing pathologist, were then used to assign an initial class to nuclei. This combination of noisy nuclear segmentation boundaries and region-derived classifications are the bootstrapped suggestions. These noisy algorithmic suggestions were the basis for annotating the Bootstrap control multi-rater dataset. A Mask R-CNN model was then used as a function approximator to smooth out some of the noise in the bootstrapped suggestions. Participants were able to view these refined suggestions, along with low-power region annotations, when annotating the single-rater and Evaluation datasets.

**Supplementary Figure S3**: Super-class accuracy of participant annotations and inferred NP-labels (Evaluation dataset). The accuracy is measured against the inferred P-truth.

**Supplementary Figure S4**: Accuracy of algorithmic suggestions (single-rater dataset). The accuracy is measured against the corrected single-rater dataset. A. Per-FOV detection accuracy of algorithmic data at the 2 stages of obtaining algorithmic suggestions; i.e., how well do the suggestions correspond to real nuclei? Mask R-CNN refinement significantly improves suggestion accuracy. B. Number of Mask R-CNN–refined suggestions that correspond to a segmentation (i.e., were clicked) or a bounding box. C. Concordance between suggested classes and classes assigned by participants. Region-based suggestions were, broadly speaking, more concordant with the true classes, but nucleus suggestions had a higher recall for sTILs. D. Comparison of the classification accuracy (MCC) of low-power region class and high-power Mask R-CNN–derived nucleus class. Numbers are normalized column-wise, i.e., represent percentages of true nuclei of a particular class. Note how region-based and nucleus-based suggestions have disparate accuracies for different FOVs and classes. Hence, there was value in providing the participants with both forms of suggestion.

**Supplementary Figure S5**: Abundance and segmentation accuracy of clicked algorithmic suggestions (multi-rater datasets). A. Proportion of nuclei in the FOV that were inferred to have good segmentation. Circle size represents the number of nuclei in that FOV. The proportion is notably higher for the Evaluation dataset than the Bootstrap control. B. Accuracy of algorithmic segmentation boundaries for nuclei that were inferred to have accurate segmentation boundaries in both the Evaluation dataset and Bootstrap control. The comparison is made against manual segmentations obtained for the same nuclei from 1 senior pathologist. Most clicked algorithmic segmentations were very accurate and have a Dice coefficient >0.8. The accuracy was slightly higher for Mask R-CNN–refined suggestions.

**Supplementary Figure S6**: Annotation procedure on HistomicsUI. A, B. Participants were shown suggestions for nucleus segmentation boundaries, as well as 2 types of classification suggestions: low-power region suggestions and high-power nucleus classification suggestions. The FOV shown here is almost entirely present in a stromal region but contains multiple scattered sTILs that were not dense enough to be captured as a sTILs “region.” C. Participants’ annotations were either points/clicks, for accurate segmentations, or bounding boxes. They picked the color/class of their annotations beforehand and were told to simply ignore any inaccurate suggestions. Participants were able to turn the suggestions off for a clear view of the underlying tissue. D. Participant annotations and algorithmic suggestions were ingested into a database and processed to provide cleaned up data, which were then pushed for viewing on HistomicsUI for correction and review.

**Supplementary Figure S7**: Confusion matrix of participant annotations (Evaluation dataset). A. Confusion of annotations placed by the participants, putting aside detection. Here, we ask the question, if a participant places an annotation that they call tumor, and it matches a true nucleus, what is the class of that nucleus? By definition, there are no “ambiguous” true nuclei. B. For each true nucleus, how many of the participants detected it, and if so, what class did they assign? Note that because truth inference takes participant reliability into account, the inferred P-truth does not have to correspond to the most commonly assigned class. Empty entries are values <1.

**Supplementary Figure S8**: Sample poor annotation data excluded during the single-rater dataset correction process. Despite having received initial training and feedback, the NP who generated these annotations was confused about what is a nucleus and frequently considered chromatin clumps or artifacts to be nuclei (arrows). This underlines the need for quality control.

**Supplementary Figure S9**: Ease of detection of various nucleus classes (Evaluation dataset). If we know for a fact that this is, say, a lymphocyte, how many participants detected it, even if they called it something else?. True class is the inferred P-truth. The color coding used is explained in panel B. A. Nuclei counts, broken down by class and the number of matched participants. B. Ease of detection of nuclei by true class. Interpreting, e.g., the blue curve proceeds as follows: 100% of lymphocytes were detected by ≥3 pathologists, ∼80% were detected by 4 pathologists, and so on.

**Supplementary Figure S10**: Algorithm for obtaining anchor proposals through constrained agglomerative clustering. We cluster bounding boxes from participants to get the anchor proposals corresponding to potential nucleus locations. Note that the threshold we use for maximum linkage, t*, is influential in determining how many anchors we get. We make sure that annotations from the same participant do not end up in the same cluster by creating sets of “do-not-link” bounding boxes. The final anchor proposals are the anchor medoids; using medoids ensures that the box anchor proposals correspond to real nucleus boundaries.

## Abbreviations

API: Application Programming Interface; AUROC: area under receiver-operator characteristic curve; BCSS: Breast Cancer Semantic Segmentation dataset; FOV: field of view; H&E: hematoxylin-eosin; IOU: intersection over union; JP: junior pathologist; MCC: Matthews correlation coefficient; NP: non-pathologist; NP-label: inferred label from multi-rater pathologist data; NuCLS: Nucleus Classification, Localization, and Segmentation; Ps: junior or senior pathologists; P-truth: inferred truth from multi-rater pathologist data; ROI: region of interest; SP: senior pathologist; TCGA: The Cancer Genome Atlas.

## Conflicts of Interest

None to disclose.

## Funding

This work was supported by the U.S. National Institutes of Health National Cancer Institute grants U01CA220401 and U24CA19436201 (Principal Investigator, L.A.D.C.). The funding body had no role in the design of the study, data collection, data analysis, or data interpretation, or writing the manuscript.

## Authors' Contributions

M.A. and L.A.D.C. conceived the hypothesis, designed the experiments, performed the analysis, and wrote the manuscript. D.M. and D.A.G. contributed support for the Digital Slide Archive software and database. P.M. provided ideas and support for the computational analysis. B.D. and D.J. provided ideas for the interrater analysis. M.A. and Maha A.T. Elsebaie were the study coordinators and corrected the single-rater dataset. H.E. provided feedback and approved the corrected single-rater dataset. E.H. provided manual nucleus segmentation data. H.E., H.H., and E.H. are senior pathologists and provided multi-rater annotations. L.A.A., K.H.M., P.A.P., and L.E.H. are junior pathologists and provided multi-rater annotations. Maha A.T. Elsebaie, A.M. Alhusseiny, M.A.A., A.M.E., R.A.S., A.R., A.M.S., A.M. Alkashash, I.A.R., A. Alrefai, N.M.E., A. Abdulkarim, A.F., A.E., A.G.E., Y.A., Y.A.A., A.M.R., M.K.N., Mai A.T. Elsebaie, A. Ayad, A.G., and M.E. are non-pathologists and provided single- and multi-rater annotations. All experience designations are based on the time of annotation. All authors reviewed the manuscript draft.

## Supplementary Material

giac037_GIGA-D-21-00352_Original_Submission

giac037_GIGA-D-21-00352_Revision_1

giac037_GIGA-D-21-00352_Revision_2

giac037_GIGA-D-21-00352_Revision_3

giac037_Response_to_Reviewer_Comments_Original_Submission

giac037_Response_to_Reviewer_Comments_Revision_1

giac037_Response_to_Reviewer_Comments_Revision_2

giac037_Reviewer_1_Report_Original_Submission Monjoy Saha, PhD -- 11/21/2021 Reviewed

giac037_Reviewer_1_Report_Revision_1 Monjoy Saha, PhD -- 1/10/2022 Reviewed

giac037_Reviewer_1_Report_Revision_2 Monjoy Saha, PhD -- 2/23/2022 Reviewed

giac037_Reviewer_2_Report_Original_Submission Chris Armit -- 11/24/2021 Reviewed

giac037_Reviewer_3_Report_Original_Submission Jin Tae Kwak -- 12/7/2021 Reviewed

giac037_Reviewer_3_Report_Revision_1 Jin Tae Kwak -- 1/26/2022 Reviewed

giac037_Supplemental_Files
