# Supplementary material for: NuCLS: A scalable crowdsourcing approach and dataset for nucleus classification and segmentation in breast cancer
Source: Gigascience. 2022 May 17;11:giac037. doi: 10.1093/gigascience/giac037 (PMC9112766; doi:10.1093/gigascience/giac037)
Supplement: giac037_GIGA-D-21-00352_Revision_3 [file giac037_giga-d-21-00352_revision_3.pdf]

GigaScience

NuCLS: A scalable crowdsourcing approach & dataset for nucleus classification and segmentation in breast cancer

--Manuscript Draft--

**Manuscript Number:**	GIGA-D-21-00352R3
**Full Title:**	NuCLS: A scalable crowdsourcing approach & dataset for nucleus classification and segmentation in breast cancer
**Article Type:**	Research
**Funding Information:**	Foundation for the National Institutes of Health (U01CA220401) — Dr Lee A.D. Cooper<br>Foundation for the National Institutes of Health (U24CA19436201) — Dr Lee A.D. Cooper
**Abstract:**	Background : Deep learning enables accurate high-resolution mapping of cells and tissue structures that can serve as the foundation of interpretable machine-learning models for computational pathology. However, generating adequate labels for these structures is a critical barrier, given the time and effort required from pathologists. Results : This paper describes a novel collaborative framework for engaging crowds of medical students and pathologists to produce quality labels for cell nuclei. We used this approach to produce the NuCLS dataset, containing over 220,000 annotations of cell nuclei in breast cancers. This builds on prior work labeling tissue regions to produce an integrated tissue region- and cell-level annotation dataset for training that is the largest such resource for multi-scale analysis of breast cancer histology. This paper presents data and analysis results for single and multi-rater annotations from both non-experts and pathologists. We present a novel workflow that uses algorithmic suggestions to collect accurate segmentation data without the need for laborious manual tracing of nuclei. Our results indicate that even noisy algorithmic suggestions do not adversely affect pathologist accuracy, and can help non-experts improve annotation quality. We also present a new approach for inferring truth from multiple raters, and show that non-experts can produce accurate annotations for visually distinctive classes. Conclusions : This study is the most extensive systematic exploration of the large-scale use of wisdom-of-the-crowd approaches to generate data for computational pathology applications.
**Corresponding Author:**	Mohamed Amgad, M.D., M.Sc.<br>Northwestern University Feinberg School of Medicine<br>Chicago, IL UNITED STATES
**Corresponding Author Secondary Information:**	
**Corresponding Author's Institution:**	Northwestern University Feinberg School of Medicine
**Corresponding Author's Secondary Institution:**	
**First Author:**	Mohamed Amgad, M.D., M.Sc.
**First Author Secondary Information:**	
**Order of Authors:**	Mohamed Amgad, M.D., M.Sc.<br>Lamees A. Atteya<br>Hagar Hussein<br>Kareem Hosny Mohammed<br>Ehab Hafiz<br>Maha A.T. Elsebaie<br>Ahmed M. Alhusseiny

Powered by Editorial Manager® and ProduXion Manager® from Aries Systems Corporation

	Mohamed Atef AlMoslemany
	Abdelmagid M. Elmatboly
	Philip A. Pappalardo
	Rokia Adel Sakr
	Pooya Mobadersany
	Ahmad Rachid
	Anas M. Saad
	Ahmad M. Alkashash
	Inas A. Ruhban
	Anas Alrefai
	Nada M. Elgazar
	Ali Abdulkarim
	Abo-Alela Farag
	Amira Etman
	Ahmed G. Elsaeed
	Yahya Alagha
	Yomna A. Amer
	Ahmed M. Raslan
	Menatalla K. Nadim
	Mai A.T. Elsebaie
	Ahmed Ayad
	Liza E. Hanna
	Ahmed Gadallah
	Mohamed Elkady
	Bradley Drumheller
	David Jaye
	David Manthey
	David A. Gutman
	Habiba Elfandy
	Lee A.D. Cooper
**Order of Authors Secondary Information:**	
**Response to Reviewers:**	We would like to thank the editor and reviewers for their support throughout this process. We have cited the GigaDB dataset as requested and submit the updated manuscript. We would also like to thank the GigaDB database curation team for their time and diligence.
**Additional Information:**	
**Question**	**Response**
Are you submitting this manuscript to a special series or article collection?	No
**Experimental design and statistics**	Yes

Full details of the experimental design and statistical methods used should be given in the Methods section, as detailed in our Minimum Standards Reporting Checklist. Information essential to interpreting the data presented should be made available in the figure legends.<br><br>Have you included all the information requested in your manuscript?	
**Resources**<br><br>A description of all resources used, including antibodies, cell lines, animals and software tools, with enough information to allow them to be uniquely identified, should be included in the Methods section. Authors are strongly encouraged to cite Research Resource Identifiers (RRIDs) for antibodies, model organisms and tools, where possible.<br><br>Have you included the information requested as detailed in our Minimum Standards Reporting Checklist?	Yes
**Availability of data and materials**<br><br>All datasets and code on which the conclusions of the paper rely must be either included in your submission or deposited in publicly available repositories (where available and ethically appropriate), referencing such data using a unique identifier in the references and in the "Availability of Data and Materials" section of your manuscript.<br><br>Have you have met the above requirement as detailed in our Minimum Standards Reporting Checklist?	No
If not, please give reasons for any omissions below.	For the time being, the dataset is hosted on Google Drive and is accessible through the official website: https://sites.google.com/view/nucls/home

Powered by Editorial Manager® and ProduXion Manager® from Aries Systems Corporation

as follow-up to "**Availability of data and materials**<br><br>All datasets and code on which the conclusions of the paper rely must be either included in your submission or deposited in publicly available repositories (where available and ethically appropriate), referencing such data using a unique identifier in the references and in the "Availability of Data and Materials" section of your manuscript.<br><br>Have you have met the above requirement as detailed in our Minimum Standards Reporting Checklist?<br><br>"	Once we pass through initial review, we will deposit the data at one of the recommended public repositories.

Powered by Editorial Manager® and ProduXion Manager® from Aries Systems Corporation

LaTeX - Main Document (TeX file)
Click here to access/download;LaTeX - Main Document (TeX file);main.tex

GigaScience, 2017, 1–11

doi: xx.xxxx/xxxx
Manuscript in Preparation
Paper

PAPER

NuCLS: A scalable crowdsourcing approach & dataset for nucleus classification and segmentation in breast cancer

Mohamed Amgad[1], Lamees A. Atteya[2,†], Hagar Hussein[3,†], Kareem Hosny Mohammed[4,†], Ehab Hafiz[5,†], Maha A.T. Elsebaie[6,†], Ahmed M. Alhusseiny[7], Mohamed Atef AlMoslemany[8], Abdelmagid M. Elmatboly[9], Philip A. Pappalardo[10], Rokia Adel Sakr[11], Pooya Mobadersany[1], Ahmad Rachid[12], Anas M. Saad[13], Ahmad M. Alkashash[14], Inas A. Ruhban[15], Anas Alrefai[12], Nada M. Elgazar[16], Ali Abdulkarim[17], Abo-Alela Farag[12], Amira Etman[8], Ahmed G. Elsaeed[16], Yahya Alagha[17], Yomna A. Amer[8], Ahmed M. Raslan[18], Menatalla K. Nadim[19], Mai A.T. Elsebaie[12], Ahmed Ayad[20], Liza E. Hanna[3], Ahmed Gadallah[12], Mohamed Elkady[21], Bradley Drumheller[22], David Jaye[22], David Manthey[23], David A. Gutman[24], Habiba Elfandy[25,26] and Lee A.D. Cooper[1,27,28,*,‡]

[1]Department of Pathology, Northwestern University, Chicago, IL, USA

[*]Address correspondence to: lee.cooper@northwestern.edu
[†]Contributed equally.
[‡]See full list of author affiliations at the end.

Abstract

Background: Deep learning enables accurate high-resolution mapping of cells and tissue structures that can serve as the foundation of interpretable machine-learning models for computational pathology. However, generating adequate labels for these structures is a critical barrier, given the time and effort required from pathologists. **Results**: This paper describes a novel collaborative framework for engaging crowds of medical students and pathologists to produce quality labels for cell nuclei. We used this approach to produce the NuCLS dataset, containing over 220,000 annotations of cell nuclei in breast cancers. This builds on prior work labeling tissue regions to produce an integrated tissue region- and cell-level annotation dataset for training that is the largest such resource for multi-scale analysis of breast cancer histology. This paper presents data and analysis results for single and multi-rater annotations from both non-experts and pathologists. We present a novel workflow that uses algorithmic suggestions to collect accurate segmentation data without the need for laborious manual tracing of nuclei. Our results indicate that even noisy algorithmic suggestions do not adversely affect pathologist accuracy, and can help non-experts improve annotation quality. We also present a new approach for inferring truth from multiple raters, and show that non-experts can produce accurate annotations for visually distinctive classes. **Conclusions**: This study is the most extensive systematic exploration of the large-scale use of wisdom-of-the-crowd approaches to generate data for computational pathology applications.

Key words: Crowdsourcing; Deep learning; Nucleus segmentation; Nucleus classification; Breast cancer.

Background

Motivation

Convolutional neural networks and other deep learning methods have been at the heart of recent advances in medicine (see Table S1 for terminology) [1]. A key challenge in computational pathology is the scarcity of large-scale labeled datasets for model training and validation [2, 3, 4]. Specifically, there is a shortage of annotation data for delineating tissue regions and cellular structures in histopathology. This information is critical for training interpretable deep-learning models, as they allow the detection of entities that are understood by pathologists and map to known diagnostic criteria [4, 5, 6, 7]. These entities can then be used to construct higher-order relational graphs that encode complex spatial and hierarchical relationships within the tumor microenvironment, paving the way for the computationally-driven discovery of histopathologic biomarkers and biological associations [4, 8, 9, 10, 11, 12, 13]. Data shortage is often attributed to the domain expertise required to produce annotation labels, with pathologists spending years in residency and fellowship training [2, 14]. This problem is exacerbated by the time constraints of clinical practice and the repetitive nature of annotation work. Manual tracing of object boundaries is an incredibly demanding task, and there is a pressing need to obtain this data using facilitated or assisted annotation strategies [15]. By comparison, traditional annotation problems like detecting people in natural images require almost no training and typically engage the general public [15]. Moreover, unique problems often require new annotation data, underscoring the need for scalable and reproducible annotation workflows [16].

We address these issues using an assisted annotation method that leverages the participation of non-pathologists (NPs), including medical students and graduates. Medical students typically have strong incentives to participate in annotation studies, with increased reliance on research participation in residency selection [17]. We describe adaptations to the data collection to improve scalability and reduce effort. This work focuses on nucleus classification, localization, and segmentation (NuCLS, for short) in whole-slide scans of Hematoxylin and Eosin-stained slides of breast carcinoma from 18 institutions from The Cancer Genome Atlas (TCGA). Our annotation pipeline enables low-effort collection of nucleus segmentation and classification data, paving the way for systematic discovery of histopathologic-genomic associations and morphological biomarkers of disease progression [4, 5, 8, 10, 11].

Related work

There has been growing interest in addressing data scarcity in histopathology by either 1. scaling data generation or 2. reducing reliance on manually labeled data using data synthesis techniques like Generative Adversarial Networks [18, 19, 20, 21, 22, 23, 24, 25]. While there is a pressing need for both approaches, this work is meant to fit into the broad context of scalable assisted manual data generation when expert annotation is expensive or difficult. Crowdsourcing, the process of engaging a "crowd" of individuals to annotate data, is critical to solving this problem. There exists a large body of relevant work in crowdsourcing for medical image analysis [15, 26, 27]. Previously, we published a study and dataset using crowdsourcing of NPs for annotation of low-power regions in breast cancer [28]. Our approach was structured because we assigned different tasks depending on the level of expertise and leveraged collaborative annotation to obtain data that is large in scale and high in quality. Here, we significantly expand this idea by focusing on the challenging problems of nucleus classification, localization, and segmentation. This computer vision problem is a subject of significant interest in computational pathology [29, 30, 31].

While the public release of data is only one aspect of our study, it is essential to acknowledge related nucleus classification datasets. Some of these datasets can be used in conjunction with ours and include MoNuSAC, CoNSep, PanNuke, and Lizard [29, 30, 32, 33, 34, 35, 36, 37, 38]. Lizard, in particular, is a highly related dataset that was recently published after we released NuCLS but focuses on colon cancer instead [37]. Additionally, the US Food and Drug Administration is leading an ongoing study to collect regulatory-grade annotations of stromal tumor-infiltrating lymphocytes (sTILs) [39]. Unfortunately, with few exceptions, most public computational pathology datasets are either limited in scale, were generated through exhaustive annotation efforts by practicing pathologists, or do not disclose or discuss data generation [2, 26, 30, 40]. Additionally, to the best of our knowledge, most other works do not explore crowdsourcing as a data generation approach or systematically explore interrater agreement for experts vs. non-experts.

A few studies are of particular relevance to this paper. A study by Irshad et al. showed that non-experts, recruited through the Figure Eight platform, can produce accurate nucleus detections and segmentations in renal clear cell cancer but was limited to 10 whole-slide images [20]. Hou et al. explored the use of synthetic data to produce nuclear segmentations [41]. While a significant contribution, their work did not address classification, relied on qualitative slide-level evaluations of results, and did not explore how algorithmic bias affects data quality [42, 22]. The approach we used involves click-based approval of annotations generated by a deep-learning algorithm. This methodological aspect is not the central focus of this paper; it is only one of many approaches for interactive segmentation and classification of nuclei explored in past studies like HistomicsML and NuClick [42, 22].

Our contributions

This work describes a scalable crowdsourcing approach that systematically engaged NPs and produced annotations for localization, segmentation, and classification of nuclei in breast cancer. Our workflow required minimal effort from pathologists and used algorithmic suggestions to scale the annotation process and obtain hybrid annotation datasets containing numerous segmentation boundaries without laborious manual tracing. We show that algorithmic suggestions can improve the accuracy of NP annotations and that NPs are reliable annotators of common cell types. In addition, we discuss a new constrained clustering method that we developed for reliable truth inference in multi-rater datasets. We also show how multi-rater data can ensure the quality of NP annotations or replace expert supervision in some contexts. Finally, we note that downstream deep-learning modeling using the NuCLS dataset is discussed in a related publication and is not the focus of this paper [43].

Compiled on: March 17, 2022.
Draft manuscript prepared by the author.

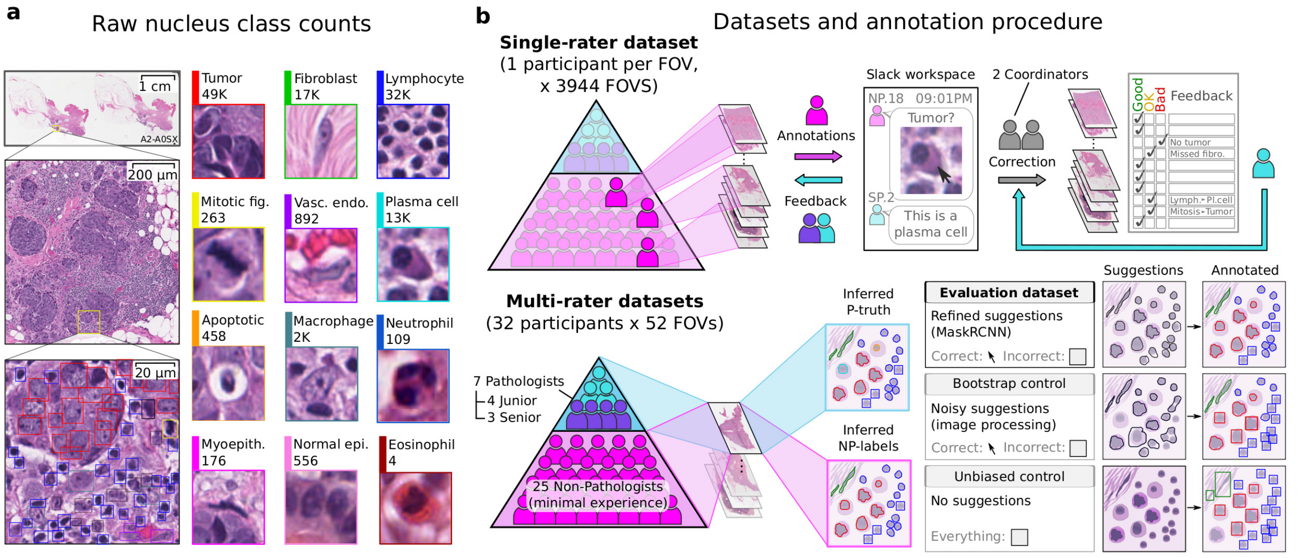

Figure 1. Dataset annotation and quality control procedure. a. Nucleus classes annotated. b. Annotation procedure and resulting datasets. Two approaches were used to obtain nucleus labels from non-pathologists (NPs). (Top) The first approach focused on breadth, collecting single-rater annotations over a large number of FOVs to obtain the majority of data in this study. NPs were given feedback on their annotations, and two study coordinators corrected and standardized all single-rater NP annotations based on input from a senior pathologist. (Bottom) The second approach evaluated interrater reliability and agreement, obtaining annotations from multiple NPs for a smaller set of shared FOVs. Annotations were also obtained from pathologists for these FOVs to measure NP reliability. The procedure for inferring a single set of labels from multiple participants is described in Figure 2. We distinguished between inferred non-pathologist labels (NP-labels) and inferred pathologist truth (P-truth) for clarity. Three multi-rater datasets were obtained: an Evaluation dataset, which is the primary multi-rater dataset, as well as Bootstrap and Unbiased experimental controls to measure the value of algorithmic suggestions. In all datasets except the Unbiased control, participants were shown algorithmic suggestions for nucleus boundaries and classes. They were directed to click nuclei with correct boundary suggestions and annotate other nuclei with bounding boxes. The pipeline to obtain algorithmic suggestions consisted of two steps: 1. Using image processing to obtain bootstrapped suggestions (Bootstrap control); 2. Training a Mask R-CNN deep-learning model to refine the bootstrapped suggestions (single-rater and Evaluation datasets).

Data Description

NuCLS is a large-scale multi-class dataset generated by engaging crowds of medical students and pathologists. NuCLS is sourced from the same images as the Breast Cancer Semantic Segmentation (BCSS) dataset [28]. Together, these datasets contain region- and cell-level annotations and constitute the most extensive resource for multi-scale analysis of breast cancer slides. We obtained a total of 222,396 nucleus annotations, including over 125,000 single-rater annotations and 97,000 multi-rater annotations. A detailed description of the dataset creation protocol is presented in the methods section.

Analyses and Discussion

Structured crowdsourcing enables scalable data collection

Pathologist time is limited and expensive, and relying solely on pathologists for generating annotations can hinder the development of state-of-the-art models based on convolutional neural networks. In this study, we show that NPs can perform most of the time-consuming annotation tasks and that pathologist involvement can be limited to low-effort tasks that include:

- Training NPs and answering their questions (Figure 1) [44].
- Qualitative scoring of NP annotations (Figure S1).
- Low-power annotation of histologic regions (Figure S2) [28].

We used a web-based annotation platform called HistomicsUI for annotation, feedback, and quality review [45]. HistomicsUI provides a user interface with annotation tools and an Application Programming Interface for programmatic querying and manipulating the centralized annotation database. The NuCLS dataset includes annotations from 32 NPs and seven pathologists in the US, Egypt, Syria, Australia, and the Maldives. We obtained 128,000 nucleus annotations from 3,944 fields-of-view (FOV) and 125 triple-negative breast cancer patients. The annotations included bounding box placement, classification, and for a sizable fraction of nuclei, segmentation boundaries. Half of these annotations underwent quality control correction based on feedback by a practicing pathologist.

Additionally, we obtained three multi-rater datasets containing 97,300 annotations, where the same FOV was annotated by multiple participants (Figure 1b, Figure 2). The collection of multi-rater data enables quantitative evaluation of NP reliability, interrater variability, and the impact of algorithmic suggestions on NP accuracy. Multi-rater annotations were *not* corrected by pathologists and enabled an unbiased assessment of NP performance. Pathologist annotations were also collected for a limited set of multi-rater FOVs to evaluate NP accuracy.

NPs can reliably classify common cell types

The detection accuracy of NPs was moderately high (AP=0.68) and was similar to the detection accuracy of pathologists. Classification accuracy of NPs, on the other hand, was only high for common nucleus classes (micro-average AUROC=0.93[0.92,0.94] vs. macro-average AUROC=0.75[0.74,0.76]) and was higher when grouping by super-class (Figure 3, Figure S3). We reported the same phenomenon in our previous work on crowdsourcing annotation of tissue regions [28]. In addition, we observed moderate clustering by participant experience (Figure 3d) and variability in classification accuracy among NPs (MCC=60.7-84.2). This observation motivated our quality control procedures. Study coordinators

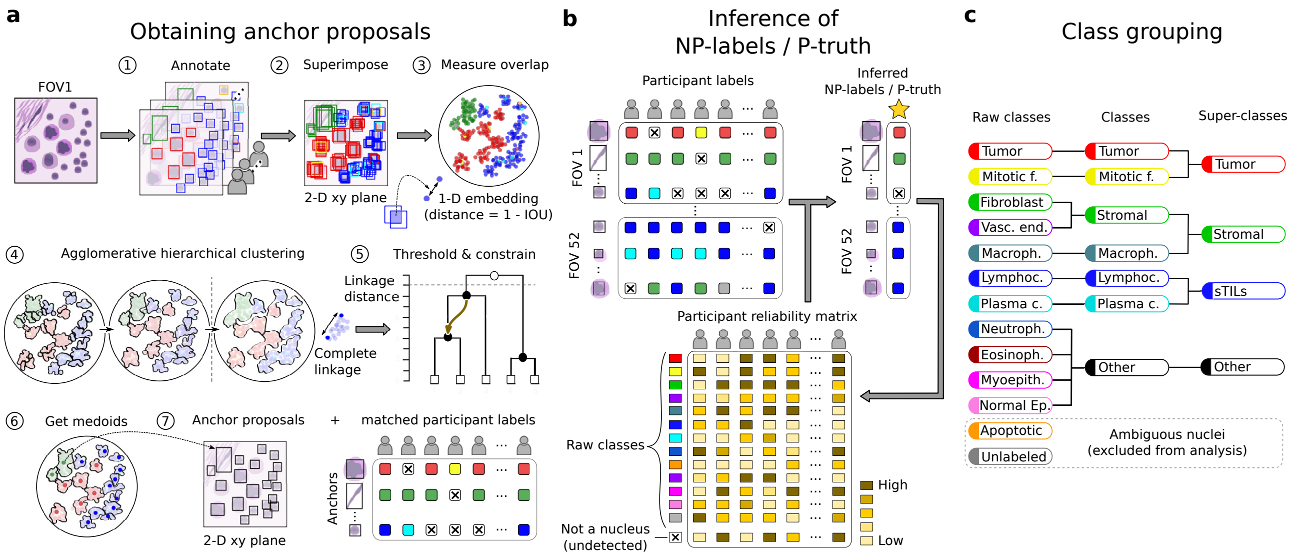

Figure 2. Inference from multi-rater datasets. The purpose of this step was to infer the nucleus locations and classifications from multi-rater data. a. The first step involved agglomerative hierarchical clustering of bounding boxes using Intersection-Over-Union (IOU) as a similarity measure. We imposed a constraint during clustering that prevents merging annotations where a single participant has annotated overlapping nuclei. Participant intention was preserved by demoting annotations from the same participant to the next node (step 5, arrow). After clustering was complete, a threshold IOU value was used to obtain the final clusters (step 5, black nodes). Within each cluster, the medoid bounding box was chosen as an anchor proposal. The result was a set of anchors with corresponding clustered annotations. When a participant did not match to an anchor, it was considered a conscious decision not to annotate a nucleus at that location. b. Once anchors were obtained, an Expectation-Maximization procedure was used to estimate: 1. which anchors represent actual nuclei, and 2. which classes to assign these anchors. The Expectation-Maximization procedure estimates and accounts for the reliability of each participant for each classification. Expectation-Maximization was performed separately for NPs and pathologists. c. Grouping of nucleus classes. Consistent with standard practice in object detection, nuclei were grouped, based on clinical reasoning, into five classes and three super-classes.

manually corrected missing or misclassified cells for the single-rater dataset, and practicing pathologists supervised and approved annotations. For the multi-rater datasets, we inferred a singular label from pathologists (P-truth) and NPs (NP-label) using an Expectation-Maximization framework that estimates reliability values for each participant [46, 47].

When pathologist supervision is not an option, multi-rater datasets need to have annotations from a sufficient number of NPs to infer reliable data. We used the annotations we obtained to perform simulations to estimate the accuracy of inferred NP-labels with fewer numbers of participating NPs (Figure 3e). The inferred NP-label accuracy increased up to six NPs per FOV, after which there were diminishing returns. Our simulations also showed that stromal nuclei require more NPs per FOV than tumor nuclei or sTILs.

Minimal-effort collection of nucleus segmentation data

Many nucleus detection and segmentation algorithms were developed using conventional image analysis methods before the widespread adoption of convolutional neural networks. These algorithms have little or no dependence on annotations, and while they may not be as accurate as convolutional neural networks, they can correctly segment a significant fraction of nuclei. We used simple nucleus segmentation heuristics, combined with low-power region annotations from the BCSS dataset, to obtain bootstrapped annotation suggestions for nuclei (Figure S2) [28]. The suggestions were refined using a well-known deep-learning model (Mask R-CNN) as a function approximator trained on the bootstrapped suggestions. This procedure allowed poor quality bootstrapped suggestions in one FOV to be smoothed by better suggestions in other FOVs (Figure S4, Table S2) and is analogous to fitting a regression line to noisy data [18, 48]. This model was applied to the FOVs to generate refined suggestions shown to participants when annotating the single-rater dataset and the Evaluation dataset (the primary multi-rater dataset) [44]. Two additional multi-rater datasets were obtained as controls:

- *Bootstrap control*: participants were shown unrefined bootstrapped suggestions.
- *Unbiased control*: participants were not shown any suggestions. This dataset was the first multi-rater dataset to be annotated.

Accurate suggestions can be confirmed during annotation with a single click, reducing effort and providing valuable nucleus boundaries that can aid the development of segmentation models. Participants can annotate nuclei that have poor suggestions using bounding boxes. Bounding box annotation requires more effort than clicking a suggestion, but less effort than the manual tracing of nuclear boundaries [15]. We obtained a substantial proportion of nucleus boundaries through clicks: 41.7±17.3% for the Evaluation dataset and 36.6% for the single-rater dataset (Figure 4, Figure S5). The resultant hybrid dataset contained a mixture of bounding boxes and accurate segmentation boundaries (Evaluation dataset DICE=85.0±5.9). We argue that it is easier to handle hybrid datasets at the level of algorithm development than to have participants trace missing boundaries or correct imprecise ones. We evaluate the bias of using these suggestions in the following section.

Algorithmic suggestions improve classification accuracy

There was value in providing the participants with suggestions for nuclear class, which included suggestions directly inherited from BCSS region annotations, as well as high-power refined suggestions produced by Mask R-CNN (Figure 4). Pathologists had substantial self-agreement when annotating FOVs with or without refined suggestions (Kappa=87.4±7.9). NPs also had

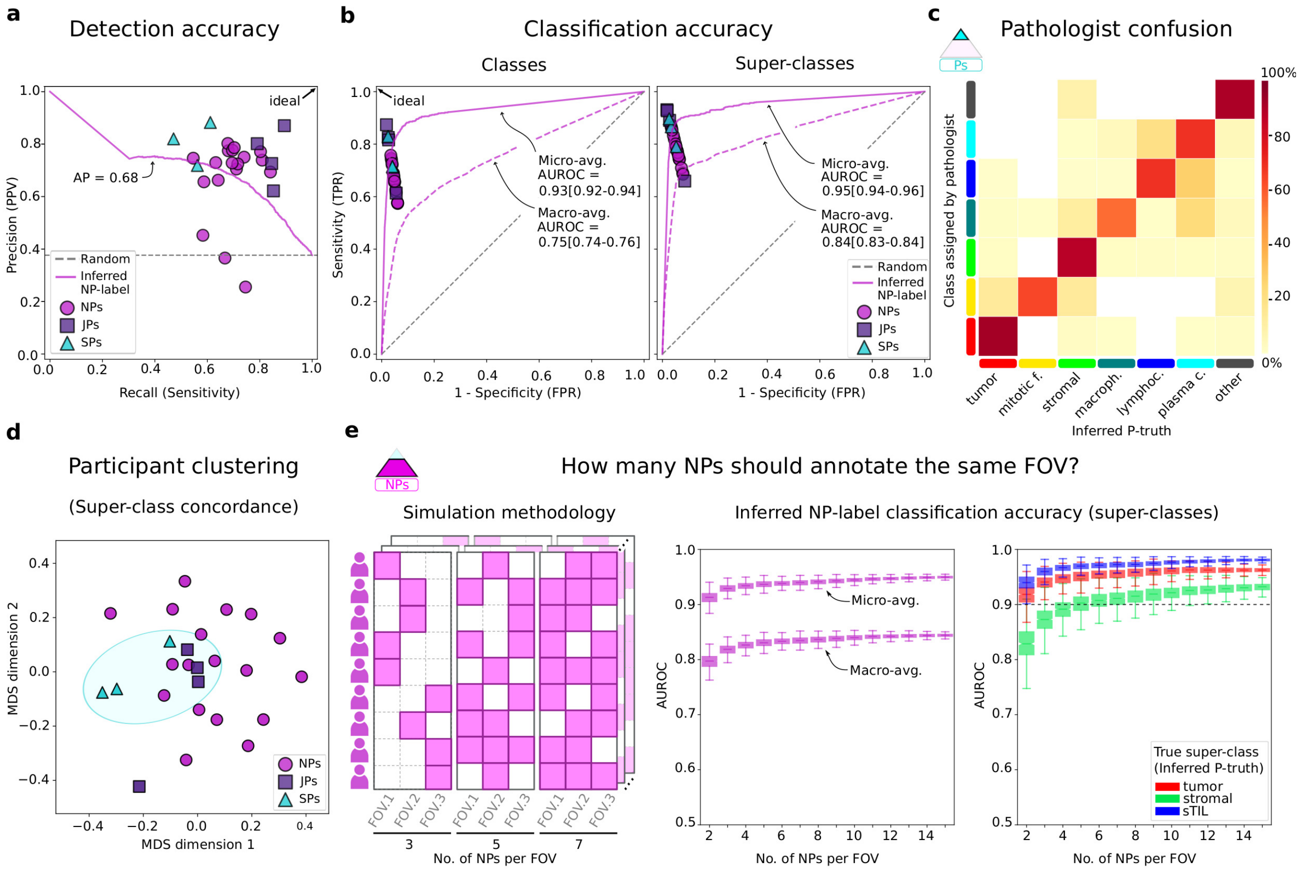

Figure 3. Accuracy of participant annotations. a. Detection precision-recall comparing annotations to inferred P-truth. Junior pathologists tend to have similar precision but higher recall than senior pathologists, possibly reflecting the time constraints of pathologists. b. Classification ROC for classes and super-classes. The overall classification accuracy of inferred NP-labels was high. However, class-balanced accuracy (macro-average) is notably lower since NPs are less reliable annotators of uncommon classes. c. Confusion between pathologist annotations and inferred P-truth. d. Multidimensional scaling (MDS) analysis of interrater classification agreement. Some clustering by participant experience (blue ellipse) highlights the importance of modeling reliability during label inference. e. A simulation was used to measure how redundancy impacts the classification accuracy of inferred NP-labels. While keeping the total number of NPs constant, we randomly kept annotations for a variable number of NPs per FOV. Accuracy in these simulations was class-dependent, with stromal nuclei requiring more redundancy for accurate inference.

high self-agreement but were more impressionable when presented with suggestions (Kappa=74.0±12.6). This was, however, associated with a reduction in bias in their annotations; refined suggestions improved the classification accuracy of inferred NP-labels (AUROC=0.95[0.94,0.96] vs. 0.92[0.90,0.93], p<0.001). This observation is consistent with Marzahl et al., who reported similar findings in a crowdsourcing study using bovine cytology slides [27].

Region-based class suggestions for nuclei were, overall, more concordant with the corrected single-rater annotations compared to Mask R-CNN refined (high-power) nucleus suggestions (MCC=67.6 vs. 52.7) (Figure S4, Table S2). Nonetheless, high-power nucleus suggestions were more accurate for 24.8% of FOVs and had a higher recall for sTILs (96.8 vs. 76.6) [4, 11]. This result makes sense since stromal regions often contain scattered sTILs, and a region-based approach to labeling would incorrectly mark these as stromal nuclei (e.g., see Figure S6) [28, 49]. Hence, the value of low and high-power classification suggestions is context-dependent.

Exploring nucleus detection and classification tradeoffs

Naturally, there is some variability in the judgments made by participants about nuclear locations and classes and the accuracy of suggested boundaries. We study the process of inferring a single truth from multi-rater datasets and discuss the effect of various parameters. There is a tradeoff between the number of nucleus anchor proposals and interrater agreement (Figure 5). The clustering IOU threshold that defines the minimum acceptable overlap between any two annotations substantially impacted the number of anchor proposals. We found that an IOU threshold of 0.25 detects most nuclei with adequate pathologist classification agreement (1,238 nuclei, Alpha=55.5). We imposed a constraint to prevent annotations from the same participant from mapping to the same cluster —this improved detection of touching nuclei when the number of pathologists was limited (Figure 5b).

Nucleus detection was a more significant source of discordance among participants than nucleus classification (Figure 3, Figure S7, Figure S8). Some nucleus classes were easier to detect than others. sTILs were the easiest to detect, likely due to their hyperchromicity and tendency to aggregate; 53.3% of sTILs were detected by 16+ NPs (Figure S9). Fibroblasts were demonstrably harder to detect (only 21.4% were detected by 16+ NPs), likely because of their relative sparsity and lighter nuclear staining. Lymphocytes and plasma cells, which often co-aggregate in lymphoplasmacytic clusters, were a source of interrater discordance for pathologists and NPs [4, 50]. This discordance may stem from variable degrees of reliance on low-power vs. high-power morphologic features. Interrater agreement for nuclear classification was high and significantly improved when classes were grouped into clinically-salient super-

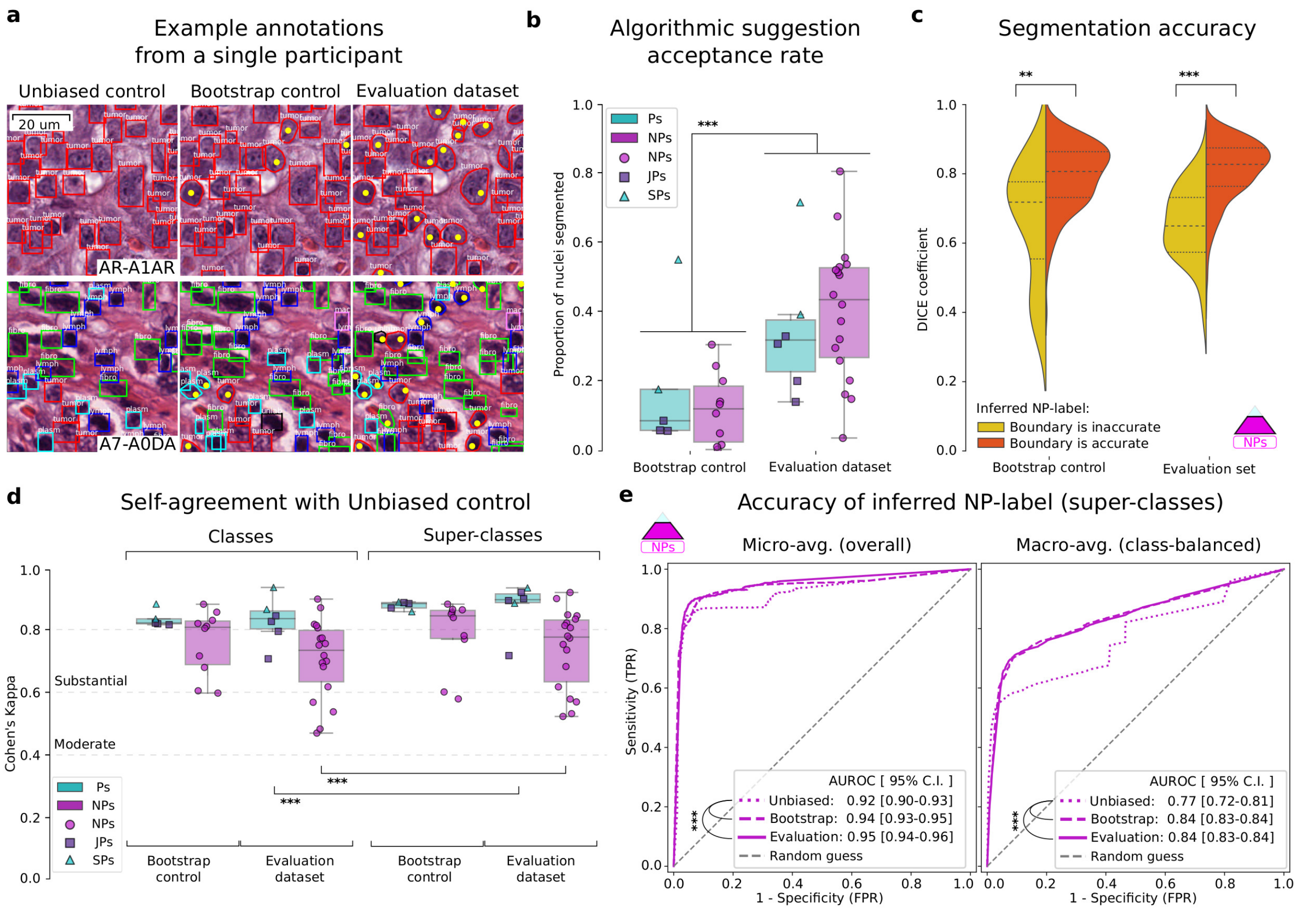

Figure 4. Effect of algorithmic suggestions on annotation abundance and accuracy. We compared annotations from the Evaluation dataset and controls to measure the impact of suggestions and Mask R-CNN refinement on the acquisition of nucleus segmentation data and the accuracy of annotations. a. Example annotations from a single participant. Algorithmic suggestions allow the collection of accurate nucleus segmentations without added effort. Yellow points indicate clicks to approve suggestions. b. The number of segmented nuclei clicked is significantly higher for the Evaluation dataset than for the Bootstrap control, indicating that refinement improves suggestion quality. c. Accuracy of algorithmic segmentation suggestions. The comparison is made against a limited set of manually traced segmentation boundaries obtained from one senior pathologist. Suggestions that were determined to be correct by the Expectation-Maximization procedure had significantly more accurate segmentation boundaries. d. Self-agreement for annotations in the presence or absence of algorithmic suggestions. The agreement is substantial for NP and pathologist groups, indicating that algorithmic suggestions do not impact classification decisions adversely. Pathologists have higher self-agreement and are less impressionable than NPs. e. ROC curves for the classification accuracy of inferred NP-label, using inferred P-truth as our reference. Statistically-significant comparisons are indicated with a star (**, p<0.01; ***, p<0.001).

classes (Alpha=66.1 (pathologists) and 60.3 (NPs); Figure 5).

Methods

Data sources

The scanned diagnostic slides we used were generated by the TCGA Research Network (https://www.cancer.gov/tcga). They were obtained from 125 patients with breast cancer (one slide per patient). Specifically, we chose to focus on all carcinoma of unspecified type cases that were triple-negative. The designation of histologic and genomic subtypes was based on public TCGA clinical records [28]. All slides were stained with Hematoxylin and Eosin and were formalin-fixed and paraffin-embedded. The scanned slides were accessed using the Digital Slide Archive repository [45].

Region annotations were obtained from BCSS, a previous crowdsourcing study that we conducted [28]. Regions of Interest (ROIs), 1 mm2 in size, were assigned to participants by difficulty level. All region annotations were corrected and approved by a practicing pathologist. These region annotations were used to obtain nucleus class suggestions as described below. Region classes included tumor, stroma, lymphocytic infiltrate, plasmacytic infiltrate, necrosis/debris, and other uncommon regions.

Algorithmic suggestions

The process for generating algorithmic suggestions is summarized in Figure S2 and involves the following steps:

Heuristic nucleus segmentation. We used simple image processing heuristics to obtain noisy nucleus segmentations [31]. Images were analyzed at scan magnification (40x) with the following steps: 1. Hematoxylin stain unmixing using the Macenko method [51]. 2. Gaussian smoothing followed by global Otsu thresholding to identify foreground nuclei pixels [52]. This step was done for each region class separately to increase robustness. We used a variance of two pixels for lymphocyte-rich regions and five pixels for other regions. 3. Connected-component analysis split the nuclei pixel mask using 8-connectivity and a 3x3 structuring element [53]. 4. We computed the Euclidean distance from every nucleus pixel to the nearest background pixel and found the peak local maxima using a minimum distance of 10 [54]. 5. A watershed segmentation algorithm split the connected components from step 3

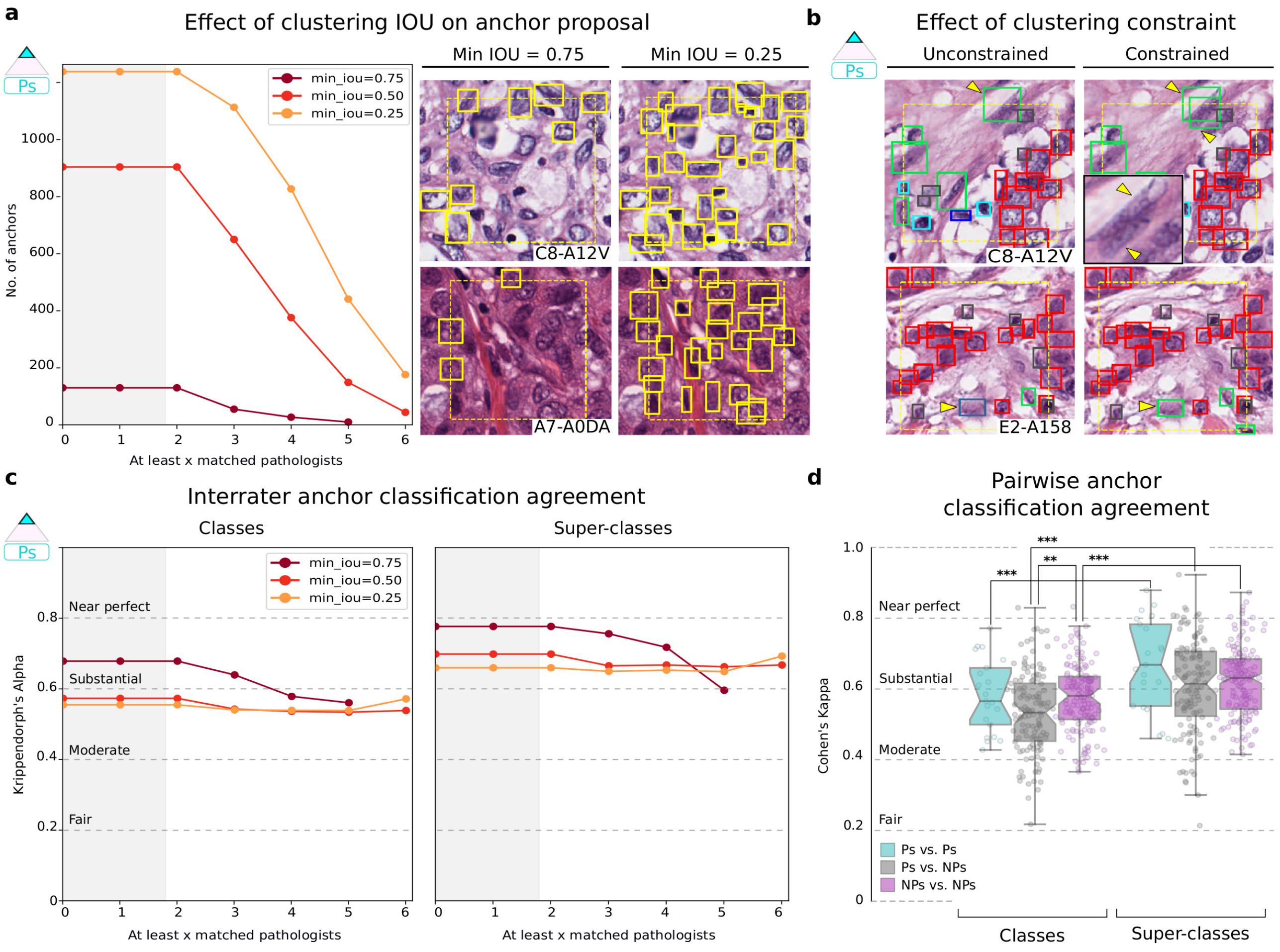

Figure 5. Effect of clustering on detection and interrater agreement. a. Stricter IOU thresholds reduce the number of anchor proposals generated by clustering but increase agreement. A threshold of 0.25 provides more anchor proposals with negligible difference in agreement from the 0.5 threshold. The shaded region indicates that by design, there are no anchor proposals with less than two clustered annotations. b. The clustering constraint prevents annotations from the same participant from being assigned to the same anchor, preserving participant intention when annotating overlapping nuclei. This results in better detection of overlapping nuclei during clustering (upper panel) and also impacts the inferred P-truth for anchors (bottom panel). c. Interrater classification agreement among pathologists for tested clustering thresholds. d. Pairwise interrater classification agreement (Cohen's Kappa) at 0.25 IOU threshold. Statistically-significant comparisons are indicated with a star (**, p<0.01; ***, p<0.001).

into individual nuclei using the local maxima from step 4 as markers [55, 56]. 6. Any object < 300 pixels in area was removed.

Bootstrapping noisy training data. Region annotations were used to assign a noisy class to each segmented nucleus. This decision was based on the observation that although tissue regions usually contain multiple cell types, there is often a single predominant cell type: tumor regions / tumor cells, stromal regions / fibroblasts, lymphocytic infiltrate / lymphocytes, plasmacytic infiltrate / plasma cells, other regions / other cells. One exception to this direct mapping is stromal regions, which contain a large number of sTILs in addition to fibroblasts. Within stromal regions, a nucleus was considered a fibroblast if it had a spindle-like shape with an aspect ratio between 0.4 and 0.55 and circularity between 0.7 and 0.8.

Mask R-CNN refinement of bootstrapped suggestions. A Mask R-CNN model with a Resnet50 backbone was used as a function approximator to refine the bootstrapped nucleus suggestions. This model was trained using randomly cropped 128x128 tiles where the number of nuclei was limited to 30. Table S3 includes other hyperparameters.

FOV sampling procedure. ROI locations were carried over from the BCSS dataset. ROIs were manually selected by a medical doctor (M.A.), who served as a study coordinator for both the BCSS and NuCLS projects, and approved by a senior pathologist (H.E.). These ROIs were then tiled into non-overlapping potential FOVs, which were automatically selected for inclusion in our study based on predefined stratified sampling criteria. 16.7% of FOVs were sampled such that the majority of refined suggestions were a single class, e.g., almost all suggestions are tumor. 16.7% were sampled to favor FOVs with two almost equally-represented classes, e.g., many tumor and fibroblast suggestions. Finally, 16.7% of FOVs were sampled to favor discordance between the bootstrapped suggestions and Mask R-CNN-refined suggestions, e.g., a stromal region with sTILs. The remaining 50% of FOVs were randomly sampled from the following pool, with the intent of favoring the annotation of difficult nuclei: a) the bottom 5% of FOVs containing high numbers of nuclei with low Mask R-CNN confidence; b) and the top 5% of FOVs containing extreme size detections, presumably clumped nuclei.

Annotation procedure and data management

The annotation protocol used is provided in the supplement. We asked the participants to annotate the single-rater dataset first because this also acted as their de-facto training. Participants were blinded to the multi-rater dataset name to avoid biasing them. The Unbiased control was annotated first for the same reason. A summary of the data management procedure is provided below.

HistomicsUI. We used the Digital Slide Archive, a web-based data management tool, to assign slides and annotation tasks (digitalslidearchive.github.io) [45]. HistomicsUI, the associated annotation interface, was used for creating, correcting, and reviewing annotations. Using a centralized setup avoids participants installing software and simplifies the dissemination of images, control over view/edit permissions, monitoring progress, and collecting results. The annotation process is illustrated in this video. The process of pathologist review of annotations is illustrated in Figure S1.

HistomicsTK Application Programming Interface. The HistomicsTK Restful Application Programming Interface (API) was used to manage data, users, and annotations programmatically. This includes uploading algorithmic suggestions, downloading participant annotations, and scalable correction of systematic annotation errors where appropriate.

Obtaining labels from multi-rater datasets

Obtaining anchor proposals. We implemented a constrained agglomerative hierarchical clustering process to obtain anchor proposals (Figure 2a). The algorithm is summarized in Figure S10. In order to have a single frame of reference for comparison, annotations from all participants and for all multi-rater datasets were clustered. After clustering, we used two rules to decide which anchor proposals corresponded to actual nuclei (for each multi-rater dataset independently): 1. At least two pathologists must detect a nucleus. 2. The inferred P-truth must concur that the anchor is a nucleus.

Inference of NP-labels and P-truth. We used the Expectation-Maximization framework described by Dawid and Skene [46, 47, 57]. Each participant was assigned an initial quality score of 0.7, and 70 Expectation-Maximization iterations were performed. As illustrated in Figure 2b, undetected was considered a nucleus class for P-truth/NP-label inference. The same process was used to infer whether the boundary of an algorithmic suggestion was accurate. In effect, the segmentation accuracy was modeled as a binary variable (clicked vs. not clicked), and the Expectation-Maximization procedure was applied to infer its value.

Class grouping

We defined two levels of grouping for nuclei classes as illustrated in Figure 2c. This was done for both the single-rater and multi-rater dataset annotations. Aggregate Expectation-Maximization probability was calculated by summing probabilities across subsets.

Participant agreement

Overall interrater agreement was measured using Krippendorff's alpha statistic, implemented in Python by Santiago Castro and Thomas Grill [58, 59, 60]. This statistic was chosen because of its ability to handle missing values [61]. Pairwise interrater agreement was measured using Cohen's Kappa statistic [62]. Likewise, self-agreement was measured using Cohen's Kappa. All of these measures range from -1 (perfect disagreement) to +1 (perfect agreement). A kappa (or alpha) value of zero represents agreement that is expected by random chance. We used thresholds set by Fleiss for defining slight, fair, moderate, substantial, and near-perfect agreement [61].

Annotation redundancy simulations

We performed simulations to measure the impact of the number of NPs assigned to each FOV on the accuracy of NP-label inference (Figure 3e). We kept the total number of NPs constant at 18 and randomly removed annotations to obtain a desired number of NPs per FOV. No constraints were placed on how many FOVs any single NP had. This simulated the realistic scenario where participants can annotate as many FOVs as they want, and our decision-making focuses on FOV assignment. For each random realization, we calculated the inferred NP-labels using Expectation-Maximization and measured accuracy against the static P-truth. This process was repeated for 1000 random realizations per configuration.

Software

Data management, machine learning models, and plotting were all implemented using Python 3+. Pytorch and Tensorflow libraries were used for various deep-learning experiments. Scikit-learn, Scikit-image, OpenCV, HistomicsTK, Scipy, Numpy, and Pandas libraries were used for matrix and image processing operations. Openslide library and HistomicsTK Application Programming Interface were used for interaction with whole-slide images.

Statistical tests

The Mann-Whitney U test was used for unpaired comparisons. The Wilcoxon signed-rank test was used for paired comparisons. Confidence bounds for the AUROC values were obtained by bootstrap sampling with replacement using 1000 trials [63, 64]. AUROC values are presented in the format: value[5th percentile, 95th percentile].

Conclusion

In summary, we have described a scalable crowdsourcing approach that benefits from the participation of NPs to reduce pathologist effort and enables minimal-effort collection of segmentation boundaries. We systematically examined aspects related to the interrater agreement and truth inference. There are important limitations and opportunities to improve on our work. Our results suggest that the participation of NPs can help address the scarcity of pathologists' availability, especially for repetitive annotation tasks. This benefit, however, is restricted to annotating predominant and visually distinctive patterns. Naturally, pathologist input — and possibly full-scale annotation effort- would be needed to supplement uncommon and challenging classes that require greater expertise. Some nuclear classes may be challenging to annotate in Hematoxylin and Eosin stained slides reliably and would be subject to considerable interrater variability even among practicing pathologists. In these settings, and where resources allow, IHC stains may be used as a more objective form of ground truth [65].

We chose to engage medical students and graduates with

the presumption that familiarity with basic histology would help acquire higher-quality data. Whether this presumption was warranted or whether it was possible to engage a broader pool of participants was not investigated. On a related note, while we observed differences based on pathologist expertise, this was not our focus. We expect to address related questions such as the value of fellowship specialization in future work. Also, we did not measure the time it took participants to create annotations; we relied on the safe assumption that certain annotation types evidently take less time and effort than others.

Another limitation is that the initial bootstrapped nuclear boundaries were generated using classical image processing methods, which tend to underperform where nuclei are highly clumped/touching or have very faint staining. This theoretically introduces some bias in our dataset, with an overrepresentation of simpler nuclear boundaries. Future work could investigate the use of transfer learning or unsupervised convolutional neural network approaches to generate more accurate algorithmic suggestions. Similarly, we used Mask R-CNN as a function approximator to refine our algorithmic suggestions. Future research can explore other deep-learning architectures that may improve refinement and result in better algorithmic suggestions.

We focused our annotation efforts on nucleus detection, as opposed to whole cells. Nuclei have distinct staining (hematoxylin) and boundaries, potentially reducing the interrater variability associated with the detection of cell boundaries. Finally, we would point out that dataset curation is context-dependent and likely differs depending on the problem. Nevertheless, we trust that most of our conclusions have broad implications for other histopathology annotation efforts.

Availability of supporting data and materials

The NuCLS dataset is available at the NuCLS website. The BCSS dataset, which helped contribute to the algorithmic suggestions, is available for download from this Github repository, and can be viewed at this demo instance of the Digital Slide Archive. Both the BCSS and NuCLS datasets are available under a CC0 1.0 license. Snapshots of our code and other data further supporting this work are openly available in the GigaScience repository, GigaDB [66].

Availability of source code and requirements

Project name: NuCLS.

Project home page: github.com/PathologyDataScience/NuCLS.

Operating system(s): Platform independent.

Programming language: Python.

Other requirements: We used this tensorflow implementation by Matterport Inc. to train the Mask R-CNN tensorflow model used for generating the algorithmic suggestions, along with a set of scripts available on Github. We used the Digital Slide Archive for whole-slide image and data management (available here), its associated annotation user interface HistomicsUI (available here), as well as the annotation and image processing library HistomicsTK (here).

License: The NuCLS codebase is licensed with an CC0 1.0 license (dataset) and the MIT license.

Restrictions to use by non-academics: Both the CC0 1.0 license (dataset) and the MIT license (codebase) allow for non-commercial use. Please review license terms for details.

Registration: *RRID*: SCR_021888. *Biotools ID*: nucls.

Declarations

List of abbreviations

AP: Average Precision; **AUROC**: Area under Receiver-Operator Characteristic curve; **BCSS**: Breast Cancer Semantic Segmentation dataset; **FOV**: Field of view; **IOU**: Intersection over union; **JPs**: Junior Pathologists; **MCC**: Matthiew's Correlation Coefficient; **NPs**: Non-pathologists; **NP-label**: Inferred label from multi-rater pathologist data; **NuCLS**: Nucleus classification, localization, and segmentation; **Ps**: Junior or senior pathologists; **P-truth**: Inferred truth from multi-rater pathologist data; **ROI**: Region of Interest; **SPs**: Senior pathologists; **TCGA**: The Cancer Genome Atlas.

Ethical Approval

Not applicable.

Consent for publication

Not applicable.

Competing Interests

The author(s) declare that they have no competing interests.

Funding

This work was supported by the U.S. National Institutes of Health National Cancer Institute grants U01CA220401 and U24CA19436201. Lee A.D. Cooper is the Principal Investigator for the grants. The funding body had no role in the design of the study, data collection, data analysis, or data interpretation, or writing the manuscript.

Author's Contributions

M.A. and L.A.D.C. conceived the hypothesis, designed the experiments, performed the analysis, and wrote the manuscript. D.M. and D.A.G. contributed support for the Digital Slide Archive software and database. B.D. and D.J. provided ideas for the interrater analysis. M.A. and M.A.T.E. were the study coordinators and corrected the single-rater dataset. H.E. provided feedback and approved the corrected single-rater dataset. E.H. provided manual nucleus segmentation data. H.E., H.H., and E.H. are senior pathologists and provided multi-rater annotations. L.A.A., K.H.M., P.A.P., and L.E.H. are junior pathologists and provided multi-rater annotations. M.A.T.E., A.M.A., M.A.A., A.M.E., R.A.S., A.R., A.M.S., A.M.A., I.A.R., A.A., N.M.E., A.A., A.F., A.E., A.G.E., Y.A., Y.A.A., A.M.R., M.K.N., M.A.T.E., A.A., A.G., and M.E. are non-pathologists and provided single- and multi-rater annotations. All experience designations are based on the time of annotation. All authors reviewed the manuscript draft.

Acknowledgements

We would like to acknowledge with gratitude the contributions made by the following participants: Eman Elsayed Sakr (El-Matariya Teaching Hospital, Egypt), Joumana Ahmed (Cairo University, Egypt); Mohamed Zalabia and Ahmed S. Badr (Menoufia University, Egypt); Ahmed M. Afifi (Ain Shams University, Egypt); Esraa B. Ghadban (Damascus University,

Syria); Mahmoud A. Hashim (Baylor College of Medicine, USA). In addition, we are thankful to Uday Kurkure, Jim Martin, Raghavan Venugopal, Joachim Schmidt (Roche Tissue Diagnostics, USA), and Michael Barnes (Roche Diagnostic Information Solutions, USA) for support and discussions. We also thank Brian Finkelman for constructive feedback on the interrater analysis. Finally, we thank Jeff Goldstein and other members of the Cooper research group at Northwestern for constructive feedback and discussion.

Full list of author affiliations

[1]Department of Pathology, Northwestern University, Chicago, IL, USA and [2]Cairo Health Care Administration, Egyptian Ministry of Health, Cairo, Egypt and [3]Department of Pathology, Nasser institute for research and treatment, Cairo, Egypt and [4]Department of Pathology and Laboratory Medicine, University of Pennsylvania, PA, USA and [5]Department of Clinical Laboratory Research, Theodor Bilharz Research Institute, Giza, Egypt and [6]Department of Medicine, Cook County Hospital, Chicago, IL, USA and [7]Department of Pathology, Baystate Medical Center, University of Massachusetts, Springfield, MA, USA and [8]Faculty of Medicine, Menoufia University, Menoufia, Egypt and [9]Faculty of Medicine, Al-Azhar University, Cairo, Egypt and [10]Consultant for The Center for Applied Proteomics and Molecular Medicine (CAPMM), George Mason University, Manassas, VA, USA and [11]Department of Pathology, National Liver Institute, Menoufia University, Menoufia, Egypt [12]Faculty of Medicine, Ain Shams University, Cairo, Egypt and [13]Cleveland Clinic Foundation, Cleveland, OH, USA and [14]Department of Pathology, Indiana University, Indianapolis, IN, USA and [15]Faculty of Medicine, Damascus University, Damascus, Syria and [16]Faculty of Medicine, Mansoura University, Mansoura, Egypt and [17]Faculty of Medicine, Cairo University, Cairo, Egypt and [18]Department of Anaesthesia and Critical Care, Menoufia University Hospital, Menoufia, Egypt and [19]Department of Clinical Pathology, Ain Shams University, Cairo, Egypt and [20]Research Department, Oncology Consultants, PA, Houston, TX, USA and [21]Siparadigm Diagnostic Informatics, Pine Brook, NJ, USA and [22]Department of Pathology and Laboratory Medicine, Emory University School of Medicine, Atlanta, GA, USA and [23]Kitware Inc., Clifton Park, NY, USA and [24]Department of Neurology, Emory University School of Medicine, Atlanta, GA, USA and [25]Department of Pathology, National Cancer Institute, Cairo, Egypt and [26]Department of Pathology, Children's Cancer Hospital Egypt (CCHE 57357), Cairo, Egypt and [27]Lurie Cancer Center, Northwestern University, Chicago, IL, USA and [28]Center for Computational Imaging and Signal Analytics, Northwestern University Feinberg School of Medicine, Chicago, IL, USA

References

1. Litjens G, Kooi T, Bejnordi BE, Setio AAA, Ciompi F, Ghafoorian M, et al. A survey on deep learning in medical image analysis. Med Image Anal 2017 Dec;42:60–88.
2. Abels E, Pantanowitz L, Aeffner F, Zarella MD, Laak J, Bui MM, et al., Computational pathology definitions, best practices, and recommendations for regulatory guidance: a white paper from the Digital Pathology Association; 2019.
3. Hartman DJ, Van Der Laak JAWM, Gurcan MN, Pantanowitz L. Value of Public Challenges for the Development of Pathology Deep Learning Algorithms. J Pathol Inform 2020 Feb;11:7.
4. Amgad M, International Immuno-Oncology Biomarker Working Group, Stovgaard ES, Balslev E, Thagaard J, Chen W, et al., Report on computational assessment of Tumor Infiltrating Lymphocytes from the International Immuno-Oncology Biomarker Working Group; 2020.
5. Beck AH, Sangoi AR, Leung S, Marinelli RJ, Nielsen TO, van de Vijver MJ, et al. Systematic analysis of breast cancer morphology uncovers stromal features associated with survival. Sci Transl Med 2011 Nov;3(108):108ra113.
6. Koh PW, Nguyen T, Tang YS, Mussmann S, Pierson E, Kim B, et al. Concept bottleneck models. In: International Conference on Machine Learning PMLR; 2020. p. 5338–5348.
7. Naik S, Doyle S, Agner S, Madabhushi A, Feldman M, Tomaszewski J, Automated gland and nuclei segmentation for grading of prostate and breast cancer histopathology; 2008.
8. Cooper LAD, Kong J, Gutman DA, Wang F, Gao J, Appin C, et al. Integrated morphologic analysis for the identification and characterization of disease subtypes. J Am Med Inform Assoc 2012 Mar;19(2):317–323.
9. Cooper LAD, Kong J, Gutman DA, Wang F, Cholleti SR, Pan TC, et al., An Integrative Approach for In Silico Glioma Research; 2010.
10. Alexander J Lazar, Michael D McLellan, Matthew H Bailey, Christopher A Miller, Elizabeth L Appelbaum, Matthew G Cordes, Catrina C Fronick, The Cancer Genome Atlas Research Network. Comprehensive and Integrated Genomic Characterization of Adult Soft Tissue Sarcomas. Cell 2017 Nov;171(4):950–965.e28.
11. Saltz J, Gupta R, Hou L, Kurc T, Singh P, Nguyen V, et al. Spatial Organization and Molecular Correlation of Tumor-Infiltrating Lymphocytes Using Deep Learning on Pathology Images. Cell Rep 2018 Apr;23(1):181–193.e7.
12. Diao JA, Wang JK, Chui WF, Mountain V, Gullapally SC, Srinivasan R, et al. Human-interpretable image features derived from densely mapped cancer pathology slides predict diverse molecular phenotypes. Nat Commun 2021 Mar;12(1):1613.
13. Lu W, Graham S, Bilal M, Rajpoot N, Minhas F. Capturing Cellular Topology in Multi-Gigapixel Pathology Images. In: Proceedings of the IEEE/CVF Conference on Computer Vision and Pattern Recognition Workshops; 2020. p. 260–261.
14. Alexander CB, Bruce Alexander C, Pathology graduate medical education (overview from 2006-2010); 2011.
15. Kovashka A, Russakovsky O, Fei-Fei L, Grauman K, Crowdsourcing in Computer Vision; 2016.
16. Ching T, Himmelstein DS, Beaulieu-Jones BK, Kalinin AA, Do BT, Way GP, et al. Opportunities and obstacles for deep learning in biology and medicine. J R Soc Interface 2018 Apr;15(141).
17. Amgad M, Man Kin Tsui M, Liptrott SJ, Shash E. Medical Student Research: An Integrated Mixed-Methods Systematic Review and Meta-Analysis. PLoS One 2015 Jun;10(6):e0127470.
18. Shaw S, Pajak M, Lisowska A, Tsaftaris SA, O'Neil AQ. Teacher-student chain for efficient semi-supervised histology image classification. arXiv preprint arXiv:200308797 2020;.
19. Hou L, Agarwal A, Samaras D, Kurc TM, Gupta RR, Saltz JH, Robust Histopathology Image Analysis: To Label or to Synthesize?; 2019.
20. Irshad H, Montaser-Kouhsari L, Waltz G, Bucur O, Nowak JA, Dong F, et al., Crowdsourcing image annotation for nucleus detection and segmentation in computational pathology: Evaluating experts, automated methods, and the crowd; 2014.
21. Campanella G, Hanna MG, Geneslaw L, Miraflor A, Werneck Krauss Silva V, Busam KJ, et al. Clinical-grade computational pathology using weakly supervised deep learning on whole slide images. Nat Med 2019 Aug;25(8):1301–1309.
22. Alemi Koohbanani N, Jahanifar M, Zamani Tajadin N, Rajpoot N. NuClick: A deep learning framework for interactive segmentation of microscopic images. Med Image Anal 2020 Oct;65:101771.
23. Deshpande S, Minhas F, Graham S, Rajpoot N. SAFRON: Stitching Across the Frontier for Generating Colorectal Cancer Histology Images. arXiv preprint arXiv:200804526 2020;.
24. Mahmood F, Borders D, Chen RJ, Mckay GN, Salimian KJ, Baras A, et al. Deep Adversarial Training for Multi-Organ Nuclei Segmentation in Histopathology Images. IEEE Trans Med Imaging 2020 Nov;39(11):3257–3267.
25. Koohbanani NA, Unnikrishnan B, Khurram SA, Krishnaswamy P, Rajpoot N. Self-Path: Self-supervision for Classification of Pathology Images with Limited Annotations. IEEE Transactions on Medical Imaging 2021;.
26. Ørting S, Doyle A, van Hilten A, Hirth M, Inel O, Madan CR, et al. A survey of crowdsourcing in medical image analysis. arXiv preprint arXiv:190209159 2019;.
27. Marzahl C, Aubreville M, Bertram CA, Gerlach S, Maier J, Voigt J, et al. Fooling the crowd with deep learning-based methods. arXiv preprint arXiv:191200142 2019;.
28. Amgad M, Elfandy H, Hussein H, Atteya LA, Elsebaie MAT,

Abo Elnasr LS, et al. Structured crowdsourcing enables convolutional segmentation of histology images. Bioinformatics 2019 Sep;35(18):3461–3467.
29. Graham S, Vu QD, Raza SEA, Azam A, Tsang YW, Kwak JT, et al. Hover-Net: Simultaneous segmentation and classification of nuclei in multi-tissue histology images. Med Image Anal 2019 Dec;58:101563.
30. Kumar N, Verma R, Anand D, Zhou Y, Onder OF, Tsougenis E, et al. A Multi-Organ Nucleus Segmentation Challenge. IEEE Trans Med Imaging 2020 May;39(5):1380–1391.
31. Xing F, Yang L. Robust Nucleus/Cell Detection and Segmentation in Digital Pathology and Microscopy Images: A Comprehensive Review. IEEE Rev Biomed Eng 2016 Jan;9:234–263.
32. Gamper J, Koohbanani NA, Benet K, Khuram A, Rajpoot N, PanNuke: An Open Pan-Cancer Histology Dataset for Nuclei Instance Segmentation and Classification; 2019.
33. Gamper J, Koohbanani NA, Benes K, Graham S, Jahanifar M, Khurram SA, et al. Pannuke dataset extension, insights and baselines. arXiv preprint arXiv:200310778 2020;.
34. Veta M, Heng YJ, Stathonikos N, Bejnordi BE, Beca F, Wollmann T, et al. Predicting breast tumor proliferation from whole-slide images: The TUPAC16 challenge. Med Image Anal 2019 May;54:111–121.
35. Janowczyk A, Madabhushi A. Deep learning for digital pathology image analysis: A comprehensive tutorial with selected use cases. J Pathol Inform 2016 Jul;7:29.
36. Verma R, Kumar N, Patil A, Kurian NC, Rane S, Sethi A. Multi-organ nuclei segmentation and classification challenge 2020. IEEE Trans Med Imaging 2020;39:1380–1391.
37. Graham S, Jahanifar M, Azam A, Nimir M, Tsang YW, Dodd K, et al. Lizard: A Large-Scale Dataset for Colonic Nuclear Instance Segmentation and Classification. In: Proceedings of the IEEE/CVF International Conference on Computer Vision; 2021. p. 684–693.
38. Verma R, Kumar N, Patil A, Kurian NC, Rane S, Graham S, et al. MoNuSAC2020: A Multi-organ Nuclei Segmentation and Classification Challenge. IEEE Trans Med Imaging 2021 Jun;PP.
39. Dudgeon SN, Wen S, Hanna MG, Gupta R, Amgad M, Sheth M, et al. A pathologist-annotated dataset for validating artificial intelligence: a project description and pilot study. J Pathol Inform 2021;12:45.
40. Litjens G, Bandi P, Ehteshami Bejnordi B, Geessink O, Balkenhol M, Bult P, et al. 1399 H&E-stained sentinel lymph node sections of breast cancer patients: the CAMELYON dataset. Gigascience 2018 Jun;7(6).
41. Hou L, Gupta R, Van Arnam JS, Zhang Y, Sivalenka K, Samaras D, et al. Dataset of segmented nuclei in hematoxylin and eosin stained histopathology images of ten cancer types. Sci Data 2020 Jun;7(1):185.
42. Nalisnik M, Amgad M, Lee S, Halani SH, Velazquez Vega JE, Brat DJ, et al. Interactive phenotyping of large-scale histology imaging data with HistomicsML. Sci Rep 2017 Nov;7(1):14588.
43. Amgad M, Atteya L, Hussein H, Mohammed KH, Hafiz E, Elsebaie MAT, et al. Explainable nucleus classification using Decision Tree Approximation of Learned Embeddings. Bioinformatics 2021 Sep;.
44. He K, Gkioxari G, Dollár P, Girshick R. Mask r-cnn. In: Proceedings of the IEEE international conference on computer vision; 2017. p. 2961–2969.
45. Gutman DA, Khalilia M, Lee S, Nalisnik M, Mullen Z, Beezley J, et al. The Digital Slide Archive: A Software Platform for Management, Integration, and Analysis of Histology for Cancer Research. Cancer Res 2017 Nov;77(21):e75–e78.
46. Dawid AP, Skene AM, Maximum Likelihood Estimation of Observer Error-Rates Using the EM Algorithm; 1979.
47. Zheng Y, Li G, Li Y, Shan C, Cheng R, Truth inference in crowdsourcing; 2017.
48. Khoreva A, Benenson R, Hosang J, Hein M, Schiele B, Simple Does It: Weakly Supervised Instance and Semantic Segmentation; 2017.
49. Amgad M, Sarkar A, Srinivas C, Redman R, Ratra S, Bechert CJ, et al. Joint Region and Nucleus Segmentation for Characterization of Tumor Infiltrating Lymphocytes in Breast Cancer. Proc SPIE Int Soc Opt Eng 2019 Feb;10956.
50. Salgado R, Denkert C, Demaria S, Sirtaine N, Klauschen F, Pruneri G, et al., The evaluation of tumor-infiltrating lymphocytes (TILs) in breast cancer: recommendations by an International TILs Working Group 2014; 2015.
51. Macenko M, Niethammer M, Marron JS, Borland D, Woosley JT, Xiaojun Guan, et al. A method for normalizing histology slides for quantitative analysis. In: 2009 IEEE International Symposium on Biomedical Imaging: From Nano to Macro ieeexplore.ieee.org; 2009. p. 1107–1110.
52. Otsu N. A threshold selection method from gray-level histograms. IEEE Trans Syst Man Cybern 1979 Jan;9(1):62–66.
53. Gonzalez R, Woods R, Digital Image Processing, (March 1992). Addison-Wesley Publishing Company; 1992.
54. Maurer CR, Rensheng Qi, Raghavan V. A linear time algorithm for computing exact Euclidean distance transforms of binary images in arbitrary dimensions. IEEE Trans Pattern Anal Mach Intell 2003 Feb;25(2):265–270.
55. Beucher S. Use of watersheds in contour detection. In: Proceedings of the International Workshop on Image Processing; 1979. .
56. Soille PJ, Ansoult MM. Automated basin delineation from digital elevation models using mathematical morphology. Signal Processing 1990 Jun;20(2):171–182.
57. Zheng Y, Li G, Li Y, Shan C, Cheng R, Crowdsourcing truth inference (Github);. Accessed: 2020-12-19. https://github.com/zhydhkcws/crowd_truth_infer.
58. Krippendorff K. Krippendorff, Klaus, Content Analysis: An Introduction to its Methodology. Beverly Hills, CA: Sage, 1980 1980;.
59. Castro S, Fast Krippendorph;. Accessed: 2020-12-19. https://github.com/pln-fing-udelar/fast-krippendorff.
60. Grill T, Krippendorph alpha;. Accessed: 2020-12-19. https://github.com/grrrr/krippendorff-alpha.
61. Fleiss JL. Measuring nominal scale agreement among many raters. Psychol Bull 1971 Nov;76(5):378–382.
62. Cohen J. A Coefficient of Agreement for Nominal Scales. Educ Psychol Meas 1960 Apr;20(1):37–46.
63. Mann HB, Whitney DR. On a Test of Whether one of Two Random Variables is Stochastically Larger than the Other. Ann Math Stat 1947;18(1):50–60.
64. Wilcoxon F. Individual Comparisons by Ranking Methods. In: Kotz S, Johnson NL, editors. Breakthroughs in Statistics: Methodology and Distribution New York, NY: Springer New York; 1992.p. 196–202.
65. Tellez D, Balkenhol M, Otte-Holler I, van de Loo R, Vogels R, Bult P, et al., Whole-Slide Mitosis Detection in H&E Breast Histology Using PHH3 as a Reference to Train Distilled Stain-Invariant Convolutional Networks; 2018.
66. Amgad M, Atteya LA, Hussein H, Mohammed KH, Hafiz E, Elsebaie MA, et al., Supporting data for "NuCLS: A scalable crowdsourcing approach & dataset for nucleus classification and segmentation in breast cancer." GigaScience Database; 2022. http://dx.doi.org/10.5524/102207.

Click here to access/download;LaTeX - Other (clo, cls, sty etc.);example-grid-100x100pt.png

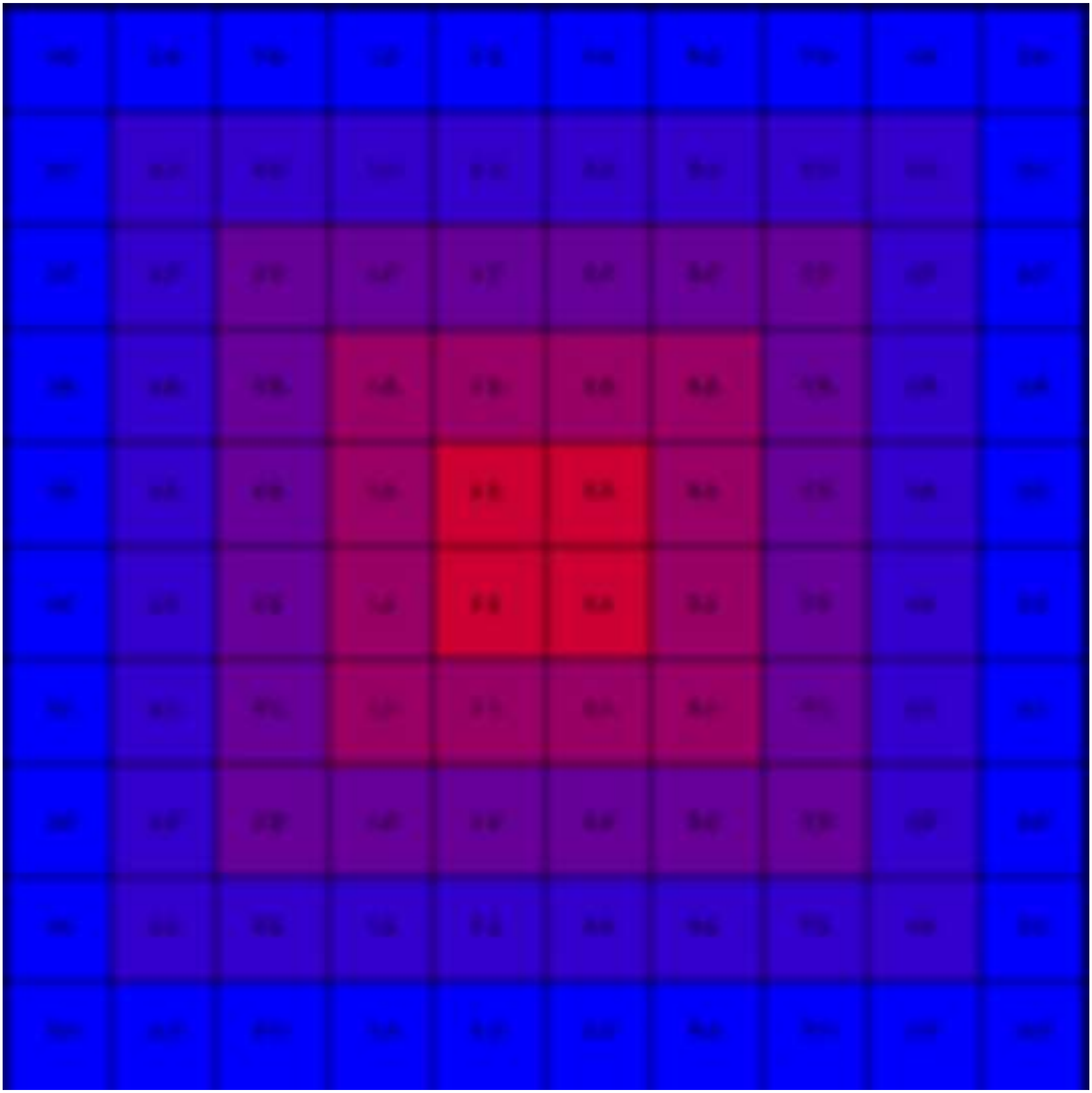

Click here to access/download;LaTeX - Other (clo, cls, sty etc.);example-image.png

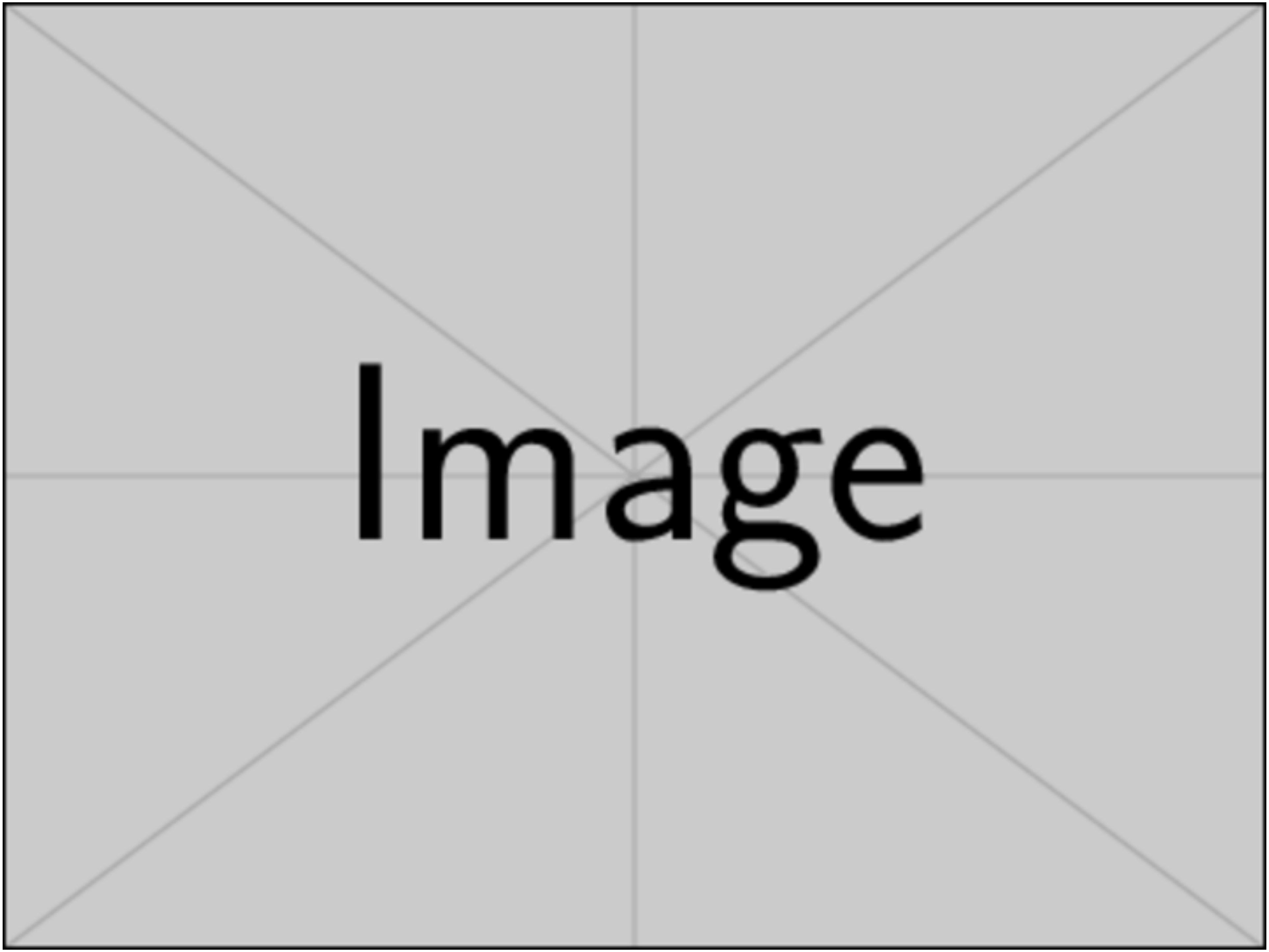

Manuscript

Click here to access/download;Manuscript;NuCLS_manuscript_revision3.pd

OXFORD

(GIGA)^n SCIENCE

GigaScience, 2017, 1–11

doi: xx.xxxx/xxxx
Manuscript in Preparation
Paper

PAPER

NuCLS: A scalable crowdsourcing approach & dataset for nucleus classification and segmentation in breast cancer

Mohamed Amgad[1], Lamees A. Atteya[2,†], Hagar Hussein[3,†], Kareem Hosny Mohammed[4,†], Ehab Hafiz[5,†], Maha A.T. Elsebaie[6,†], Ahmed M. Alhusseiny[7], Mohamed Atef AlMoslemany[8], Abdelmagid M. Elmatboly[9], Philip A. Pappalardo[10], Rokia Adel Sakr[11], Pooya Mobadersany[1], Ahmad Rachid[12], Anas M. Saad[13], Ahmad M. Alkashash[14], Inas A. Ruhban[15], Anas Alrefai[12], Nada M. Elgazar[16], Ali Abdulkarim[17], Abo-Alela Farag[12], Amira Etman[8], Ahmed G. Elsaeed[16], Yahya Alagha[17], Yomna A. Amer[8], Ahmed M. Raslan[18], Menatalla K. Nadim[19], Mai A.T. Elsebaie[12], Ahmed Ayad[20], Liza E. Hanna[3], Ahmed Gadallah[12], Mohamed Elkady[21], Bradley Drumheller[22], David Jaye[22], David Manthey[23], David A. Gutman[24], Habiba Elfandy[25,26] and Lee A.D. Cooper[1,27,28,*,‡]

[1]Department of Pathology, Northwestern University, Chicago, IL, USA

[*]Address correspondence to: lee.cooper@northwestern.edu
[†]Contributed equally.
[‡]See full list of author affiliations at the end.

Abstract

Background: Deep learning enables accurate high-resolution mapping of cells and tissue structures that can serve as the foundation of interpretable machine-learning models for computational pathology. However, generating adequate labels for these structures is a critical barrier, given the time and effort required from pathologists. **Results**: This paper describes a novel collaborative framework for engaging crowds of medical students and pathologists to produce quality labels for cell nuclei. We used this approach to produce the NuCLS dataset, containing over 220,000 annotations of cell nuclei in breast cancers. This builds on prior work labeling tissue regions to produce an integrated tissue region- and cell-level annotation dataset for training that is the largest such resource for multi-scale analysis of breast cancer histology. This paper presents data and analysis results for single and multi-rater annotations from both non-experts and pathologists. We present a novel workflow that uses algorithmic suggestions to collect accurate segmentation data without the need for laborious manual tracing of nuclei. Our results indicate that even noisy algorithmic suggestions do not adversely affect pathologist accuracy, and can help non-experts improve annotation quality. We also present a new approach for inferring truth from multiple raters, and show that non-experts can produce accurate annotations for visually distinctive classes. **Conclusions**: This study is the most extensive systematic exploration of the large-scale use of wisdom-of-the-crowd approaches to generate data for computational pathology applications.

Key words: Crowdsourcing; Deep learning; Nucleus segmentation; Nucleus classification; Breast cancer.

Background

Motivation

Convolutional neural networks and other deep learning methods have been at the heart of recent advances in medicine (see Table S1 for terminology) [1]. A key challenge in computational pathology is the scarcity of large-scale labeled datasets for model training and validation [2, 3, 4]. Specifically, there is a shortage of annotation data for delineating tissue regions and cellular structures in histopathology. This information is critical for training interpretable deep-learning models, as they allow the detection of entities that are understood by pathologists and map to known diagnostic criteria [4, 5, 6, 7]. These entities can then be used to construct higher-order relational graphs that encode complex spatial and hierarchical relationships within the tumor microenvironment, paving the way for the computationally-driven discovery of histopathologic biomarkers and biological associations [4, 8, 9, 10, 11, 12, 13]. Data shortage is often attributed to the domain expertise required to produce annotation labels, with pathologists spending years in residency and fellowship training [2, 14]. This problem is exacerbated by the time constraints of clinical practice and the repetitive nature of annotation work. Manual tracing of object boundaries is an incredibly demanding task, and there is a pressing need to obtain this data using facilitated or assisted annotation strategies [15]. By comparison, traditional annotation problems like detecting people in natural images require almost no training and typically engage the general public [15]. Moreover, unique problems often require new annotation data, underscoring the need for scalable and reproducible annotation workflows [16].

We address these issues using an assisted annotation method that leverages the participation of non-pathologists (NPs), including medical students and graduates. Medical students typically have strong incentives to participate in annotation studies, with increased reliance on research participation in residency selection [17]. We describe adaptations to the data collection to improve scalability and reduce effort. This work focuses on nucleus classification, localization, and segmentation (NuCLS, for short) in whole-slide scans of Hematoxylin and Eosin-stained slides of breast carcinoma from 18 institutions from The Cancer Genome Atlas (TCGA). Our annotation pipeline enables low-effort collection of nucleus segmentation and classification data, paving the way for systematic discovery of histopathologic-genomic associations and morphological biomarkers of disease progression [4, 5, 8, 10, 11].

Related work

There has been growing interest in addressing data scarcity in histopathology by either 1. scaling data generation or 2. reducing reliance on manually labeled data using data synthesis techniques like Generative Adversarial Networks [18, 19, 20, 21, 22, 23, 24, 25]. While there is a pressing need for both approaches, this work is meant to fit into the broad context of scalable assisted manual data generation when expert annotation is expensive or difficult. Crowdsourcing, the process of engaging a "crowd" of individuals to annotate data, is critical to solving this problem. There exists a large body of relevant work in crowdsourcing for medical image analysis [15, 26, 27]. Previously, we published a study and dataset using crowdsourcing of NPs for annotation of low-power regions in breast cancer [28]. Our approach was structured because we assigned different tasks depending on the level of expertise and leveraged collaborative annotation to obtain data that is large in scale and high in quality. Here, we significantly expand this idea by focusing on the challenging problems of nucleus classification, localization, and segmentation. This computer vision problem is a subject of significant interest in computational pathology [29, 30, 31].

While the public release of data is only one aspect of our study, it is essential to acknowledge related nucleus classification datasets. Some of these datasets can be used in conjunction with ours and include MoNuSAC, CoNSep, PanNuke, and Lizard [29, 30, 32, 33, 34, 35, 36, 37, 38]. Lizard, in particular, is a highly related dataset that was recently published after we released NuCLS but focuses on colon cancer instead [37]. Additionally, the US Food and Drug Administration is leading an ongoing study to collect regulatory-grade annotations of stromal tumor-infiltrating lymphocytes (sTILs) [39]. Unfortunately, with few exceptions, most public computational pathology datasets are either limited in scale, were generated through exhaustive annotation efforts by practicing pathologists, or do not disclose or discuss data generation [2, 26, 30, 40]. Additionally, to the best of our knowledge, most other works do not explore crowdsourcing as a data generation approach or systematically explore interrater agreement for experts vs. non-experts.

A few studies are of particular relevance to this paper. A study by Irshad et al. showed that non-experts, recruited through the Figure Eight platform, can produce accurate nucleus detections and segmentations in renal clear cell cancer but was limited to 10 whole-slide images [20]. Hou et al. explored the use of synthetic data to produce nuclear segmentations [41]. While a significant contribution, their work did not address classification, relied on qualitative slide-level evaluations of results, and did not explore how algorithmic bias affects data quality [42, 22]. The approach we used involves click-based approval of annotations generated by a deep-learning algorithm. This methodological aspect is not the central focus of this paper; it is only one of many approaches for interactive segmentation and classification of nuclei explored in past studies like HistomicsML and NuClick [42, 22].

Our contributions

This work describes a scalable crowdsourcing approach that systematically engaged NPs and produced annotations for localization, segmentation, and classification of nuclei in breast cancer. Our workflow required minimal effort from pathologists and used algorithmic suggestions to scale the annotation process and obtain hybrid annotation datasets containing numerous segmentation boundaries without laborious manual tracing. We show that algorithmic suggestions can improve the accuracy of NP annotations and that NPs are reliable annotators of common cell types. In addition, we discuss a new constrained clustering method that we developed for reliable truth inference in multi-rater datasets. We also show how multi-rater data can ensure the quality of NP annotations or replace expert supervision in some contexts. Finally, we note that downstream deep-learning modeling using the NuCLS dataset is discussed in a related publication and is not the focus of this paper [43].

Data Description

NuCLS is a large-scale multi-class dataset generated by engaging crowds of medical students and pathologists. NuCLS is sourced from the same images as the Breast Cancer Semantic Segmentation (BCSS) dataset [28]. Together, these datasets contain region- and cell-level annotations and constitute the most extensive resource for multi-scale analysis of breast cancer slides. We obtained a total of 222,396 nucleus annotations, including over 125,000 single-rater

Compiled on: March 17, 2022.
Draft manuscript prepared by the author.

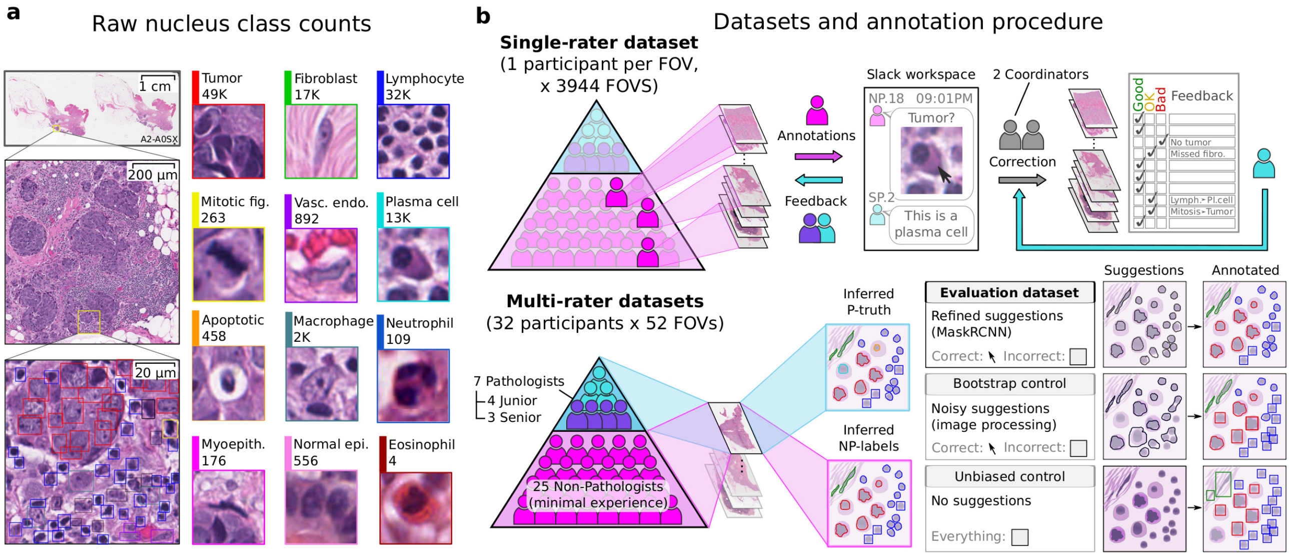

Figure 1. Dataset annotation and quality control procedure. a. Nucleus classes annotated. b. Annotation procedure and resulting datasets. Two approaches were used to obtain nucleus labels from non-pathologists (NPs). (Top) The first approach focused on breadth, collecting single-rater annotations over a large number of FOVs to obtain the majority of data in this study. NPs were given feedback on their annotations, and two study coordinators corrected and standardized all single-rater NP annotations based on input from a senior pathologist. (Bottom) The second approach evaluated interrater reliability and agreement, obtaining annotations from multiple NPs for a smaller set of shared FOVs. Annotations were also obtained from pathologists for these FOVs to measure NP reliability. The procedure for inferring a single set of labels from multiple participants is described in Figure 2. We distinguished between inferred non-pathologist labels (NP-labels) and inferred pathologist truth (P-truth) for clarity. Three multi-rater datasets were obtained: an Evaluation dataset, which is the primary multi-rater dataset, as well as Bootstrap and Unbiased experimental controls to measure the value of algorithmic suggestions. In all datasets except the Unbiased control, participants were shown algorithmic suggestions for nucleus boundaries and classes. They were directed to click nuclei with correct boundary suggestions and annotate other nuclei with bounding boxes. The pipeline to obtain algorithmic suggestions consisted of two steps: 1. Using image processing to obtain bootstrapped suggestions (Bootstrap control); 2. Training a Mask R-CNN deep-learning model to refine the bootstrapped suggestions (single-rater and Evaluation datasets).

annotations and 97,000 multi-rater annotations. A detailed description of the dataset creation protocol is presented in the methods section.

Analyses and Discussion

Structured crowdsourcing enables scalable data collection

Pathologist time is limited and expensive, and relying solely on pathologists for generating annotations can hinder the development of state-of-the-art models based on convolutional neural networks. In this study, we show that NPs can perform most of the time-consuming annotation tasks and that pathologist involvement can be limited to low-effort tasks that include:

- Training NPs and answering their questions (Figure 1) [44].
- Qualitative scoring of NP annotations (Figure S1).
- Low-power annotation of histologic regions (Figure S2) [28].

We used a web-based annotation platform called HistomicsUI for annotation, feedback, and quality review [45]. HistomicsUI provides a user interface with annotation tools and an Application Programming Interface for programmatic querying and manipulating the centralized annotation database. The NuCLS dataset includes annotations from 32 NPs and seven pathologists in the US, Egypt, Syria, Australia, and the Maldives. We obtained 128,000 nucleus annotations from 3,944 fields-of-view (FOV) and 125 triple-negative breast cancer patients. The annotations included bounding box placement, classification, and for a sizable fraction of nuclei, segmentation boundaries. Half of these annotations underwent quality control correction based on feedback by a practicing pathologist.

Additionally, we obtained three multi-rater datasets containing 97,300 annotations, where the same FOV was annotated by multiple participants (Figure 1b, Figure 2). The collection of multi-rater data enables quantitative evaluation of NP reliability, interrater variability, and the impact of algorithmic suggestions on NP accuracy. Multi-rater annotations were *not* corrected by pathologists and enabled an unbiased assessment of NP performance. Pathologist annotations were also collected for a limited set of multi-rater FOVs to evaluate NP accuracy.

NPs can reliably classify common cell types

The detection accuracy of NPs was moderately high (AP=0.68) and was similar to the detection accuracy of pathologists. Classification accuracy of NPs, on the other hand, was only high for common nucleus classes (micro-average AUROC=0.93[0.92,0.94] vs. macro-average AUROC=0.75[0.74,0.76]) and was higher when grouping by super-class (Figure 3, Figure S3). We reported the same phenomenon in our previous work on crowdsourcing annotation of tissue regions [28]. In addition, we observed moderate clustering by participant experience (Figure 3d) and variability in classification accuracy among NPs (MCC=60.7-84.2). This observation motivated our quality control procedures. Study coordinators manually corrected missing or misclassified cells for the single-rater dataset, and practicing pathologists supervised and approved annotations. For the multi-rater datasets, we inferred a singular label from pathologists (P-truth) and NPs (NP-label) using an Expectation-Maximization framework that estimates reliability values for each participant [46, 47].

When pathologist supervision is not an option, multi-rater datasets need to have annotations from a sufficient number of NPs to infer reliable data. We used the annotations we obtained to perform simulations to estimate the accuracy of inferred NP-labels with fewer numbers of participating NPs (Figure 3e). The inferred NP-label accuracy increased up to six NPs per FOV, after which there were diminishing returns. Our simulations also showed that stromal nuclei require more NPs per FOV than tumor nuclei or sTILs.

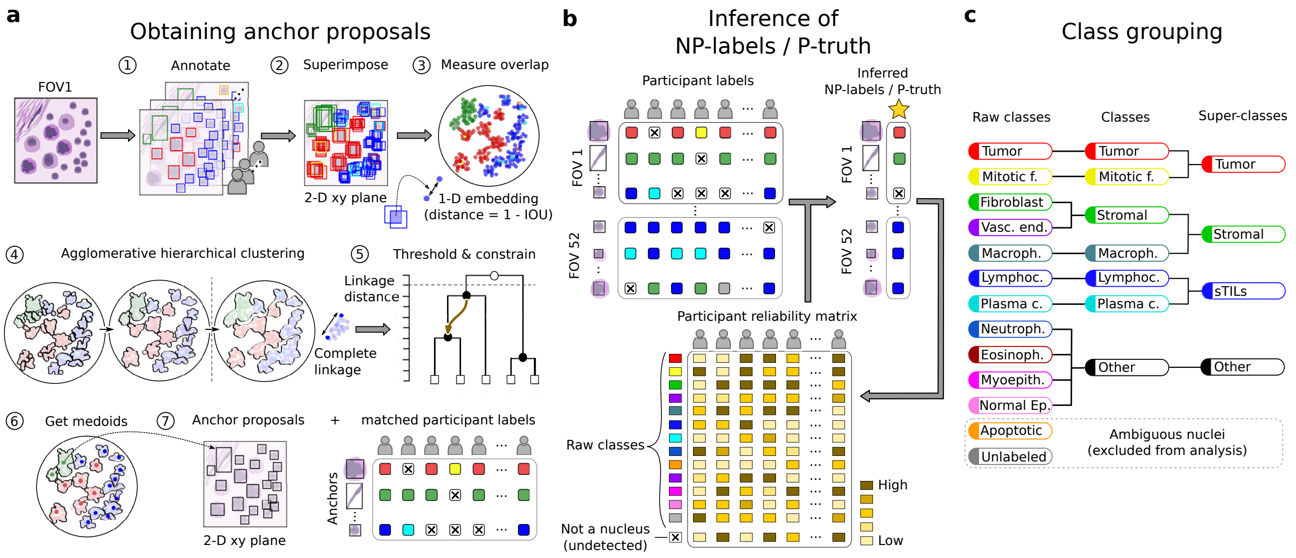

Figure 2. Inference from multi-rater datasets. The purpose of this step was to infer the nucleus locations and classifications from multi-rater data. a. The first step involved agglomerative hierarchical clustering of bounding boxes using Intersection-Over-Union (IOU) as a similarity measure. We imposed a constraint during clustering that prevents merging annotations where a single participant has annotated overlapping nuclei. Participant intention was preserved by demoting annotations from the same participant to the next node (step 5, arrow). After clustering was complete, a threshold IOU value was used to obtain the final clusters (step 5, black nodes). Within each cluster, the medoid bounding box was chosen as an anchor proposal. The result was a set of anchors with corresponding clustered annotations. When a participant did not match to an anchor, it was considered a conscious decision not to annotate a nucleus at that location. b. Once anchors were obtained, an Expectation-Maximization procedure was used to estimate: 1. which anchors represent actual nuclei, and 2. which classes to assign these anchors. The Expectation-Maximization procedure estimates and accounts for the reliability of each participant for each classification. Expectation-Maximization was performed separately for NPs and pathologists. c. Grouping of nucleus classes. Consistent with standard practice in object detection, nuclei were grouped, based on clinical reasoning, into five classes and three super-classes.

Minimal-effort collection of nucleus segmentation data

Many nucleus detection and segmentation algorithms were developed using conventional image analysis methods before the widespread adoption of convolutional neural networks. These algorithms have little or no dependence on annotations, and while they may not be as accurate as convolutional neural networks, they can correctly segment a significant fraction of nuclei. We used simple nucleus segmentation heuristics, combined with low-power region annotations from the BCSS dataset, to obtain bootstrapped annotation suggestions for nuclei (Figure S2) [28]. The suggestions were refined using a well-known deep-learning model (Mask R-CNN) as a function approximator trained on the bootstrapped suggestions. This procedure allowed poor quality bootstrapped suggestions in one FOV to be smoothed by better suggestions in other FOVs (Figure S4, Table S2) and is analogous to fitting a regression line to noisy data [18, 48]. This model was applied to the FOVs to generate refined suggestions shown to participants when annotating the single-rater dataset and the Evaluation dataset (the primary multi-rater dataset) [44]. Two additional multi-rater datasets were obtained as controls:

- *Bootstrap control*: participants were shown unrefined bootstrapped suggestions.
- *Unbiased control*: participants were not shown any suggestions. This dataset was the first multi-rater dataset to be annotated.

Accurate suggestions can be confirmed during annotation with a single click, reducing effort and providing valuable nucleus boundaries that can aid the development of segmentation models. Participants can annotate nuclei that have poor suggestions using bounding boxes. Bounding box annotation requires more effort than clicking a suggestion, but less effort than the manual tracing of nuclear boundaries [15]. We obtained a substantial proportion of nucleus boundaries through clicks: 41.7±17.3% for the Evaluation dataset and 36.6% for the single-rater dataset (Figure 4, Figure S5). The resultant hybrid dataset contained a mixture of bounding boxes and accurate segmentation boundaries (Evaluation dataset DICE=85.0±5.9). We argue that it is easier to handle hybrid datasets at the level of algorithm development than to have participants trace missing boundaries or correct imprecise ones. We evaluate the bias of using these suggestions in the following section.

Algorithmic suggestions improve classification accuracy

There was value in providing the participants with suggestions for nuclear class, which included suggestions directly inherited from BCSS region annotations, as well as high-power refined suggestions produced by Mask R-CNN (Figure 4). Pathologists had substantial self-agreement when annotating FOVs with or without refined suggestions (Kappa=87.4±7.9). NPs also had high self-agreement but were more impressionable when presented with suggestions (Kappa=74.0±12.6). This was, however, associated with a reduction in bias in their annotations; refined suggestions improved the classification accuracy of inferred NP-labels (AUROC=0.95[0.94,0.96] vs. 0.92[0.90,0.93], p<0.001). This observation is consistent with Marzahl et al., who reported similar findings in a crowdsourcing study using bovine cytology slides [27].

Region-based class suggestions for nuclei were, overall, more concordant with the corrected single-rater annotations compared to Mask R-CNN refined (high-power) nucleus suggestions (MCC=67.6 vs. 52.7) (Figure S4, Table S2). Nonetheless, high-power nucleus suggestions were more accurate for 24.8% of FOVs and had a higher recall for sTILs (96.8 vs. 76.6) [4, 11]. This result makes sense since stromal regions often contain scattered sTILs, and a region-based approach to labeling would incorrectly mark these as stromal nuclei (e.g., see Figure S6) [28, 49]. Hence, the value of low and high-power classification suggestions is context-dependent.

Exploring nucleus detection and classification tradeoffs

Naturally, there is some variability in the judgments made by participants about nuclear locations and classes and the accuracy of suggested boundaries. We study the process of inferring a single

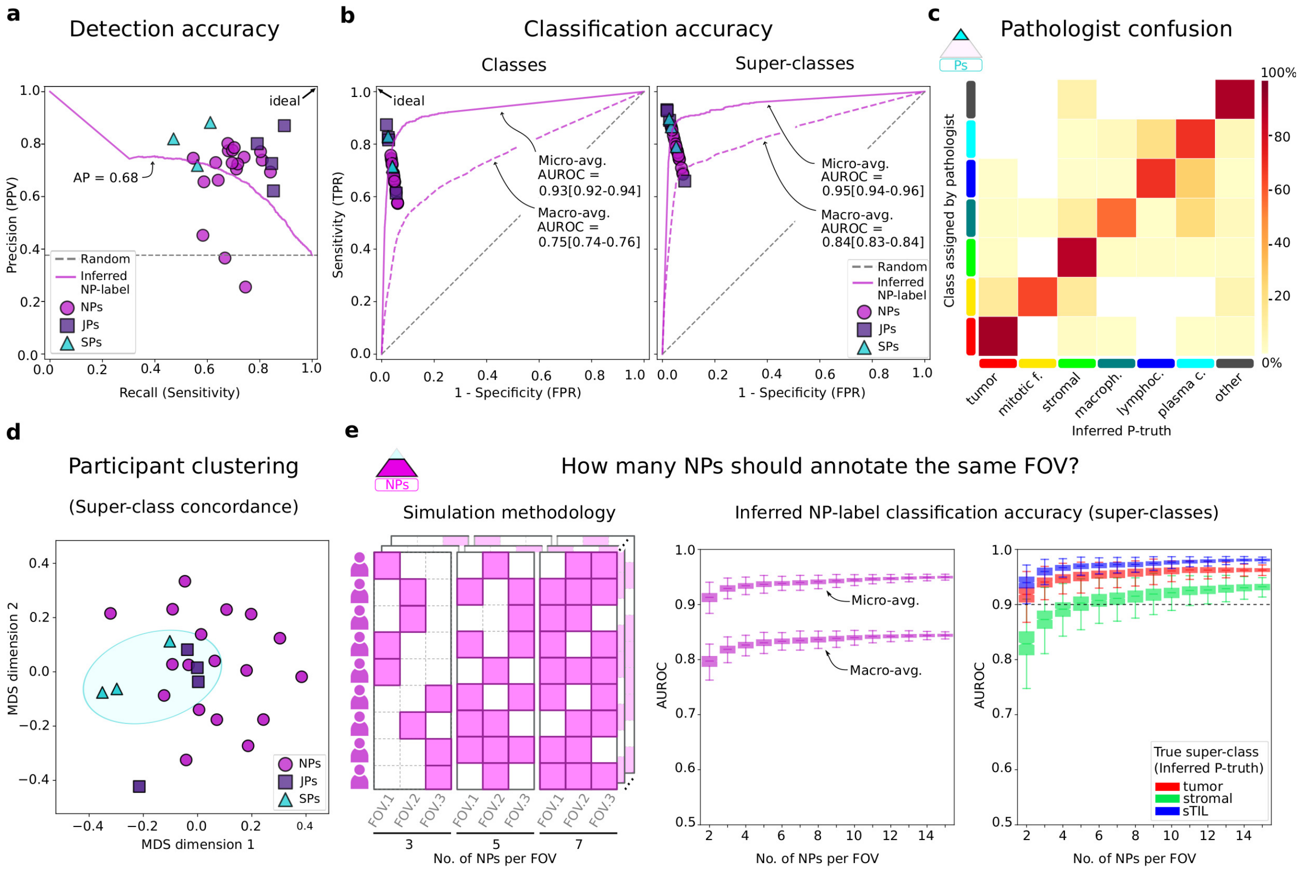

Figure 3. Accuracy of participant annotations. a. Detection precision-recall comparing annotations to inferred P-truth. Junior pathologists tend to have similar precision but higher recall than senior pathologists, possibly reflecting the time constraints of pathologists. b. Classification ROC for classes and super-classes. The overall classification accuracy of inferred NP-labels was high. However, class-balanced accuracy (macro-average) is notably lower since NPs are less reliable annotators of uncommon classes. c. Confusion between pathologist annotations and inferred P-truth. d. Multidimensional scaling (MDS) analysis of interrater classification agreement. Some clustering by participant experience (blue ellipse) highlights the importance of modeling reliability during label inference. e. A simulation was used to measure how redundancy impacts the classification accuracy of inferred NP-labels. While keeping the total number of NPs constant, we randomly kept annotations for a variable number of NPs per FOV. Accuracy in these simulations was class-dependent, with stromal nuclei requiring more redundancy for accurate inference.

truth from multi-rater datasets and discuss the effect of various parameters. There is a tradeoff between the number of nucleus anchor proposals and interrater agreement (Figure 5). The clustering IOU threshold that defines the minimum acceptable overlap between any two annotations substantially impacted the number of anchor proposals. We found that an IOU threshold of 0.25 detects most nuclei with adequate pathologist classification agreement (1,238 nuclei, Alpha=55.5). We imposed a constraint to prevent annotations from the same participant from mapping to the same cluster —this improved detection of touching nuclei when the number of pathologists was limited (Figure 5b).

Nucleus detection was a more significant source of discordance among participants than nucleus classification (Figure 3, Figure S7, Figure S8). Some nucleus classes were easier to detect than others. sTILs were the easiest to detect, likely due to their hyperchromicity and tendency to aggregate; 53.3% of sTILs were detected by 16+ NPs (Figure S9). Fibroblasts were demonstrably harder to detect (only 21.4% were detected by 16+ NPs), likely because of their relative sparsity and lighter nuclear staining. Lymphocytes and plasma cells, which often co-aggregate in lymphoplasmacytic clusters, were a source of interrater discordance for pathologists and NPs [4, 50]. This discordance may stem from variable degrees of reliance on low-power vs. high-power morphologic features. Interrater agreement for nuclear classification was high and significantly improved when classes were grouped into clinically-salient super-classes (Alpha=66.1 (pathologists) and 60.3 (NPs); Figure 5).

Methods

Data sources

The scanned diagnostic slides we used were generated by the TCGA Research Network (https://www.cancer.gov/tcga). They were obtained from 125 patients with breast cancer (one slide per patient). Specifically, we chose to focus on all carcinoma of unspecified type cases that were triple-negative. The designation of histologic and genomic subtypes was based on public TCGA clinical records [28]. All slides were stained with Hematoxylin and Eosin and were formalin-fixed and paraffin-embedded. The scanned slides were accessed using the Digital Slide Archive repository [45].

Region annotations were obtained from BCSS, a previous crowdsourcing study that we conducted [28]. Regions of Interest (ROIs), 1 mm2 in size, were assigned to participants by difficulty level. All region annotations were corrected and approved by a practicing pathologist. These region annotations were used to obtain nucleus class suggestions as described below. Region classes included tumor, stroma, lymphocytic infiltrate, plasmacytic infiltrate, necrosis/debris, and other uncommon regions.

Algorithmic suggestions

The process for generating algorithmic suggestions is summarized in Figure S2 and involves the following steps:

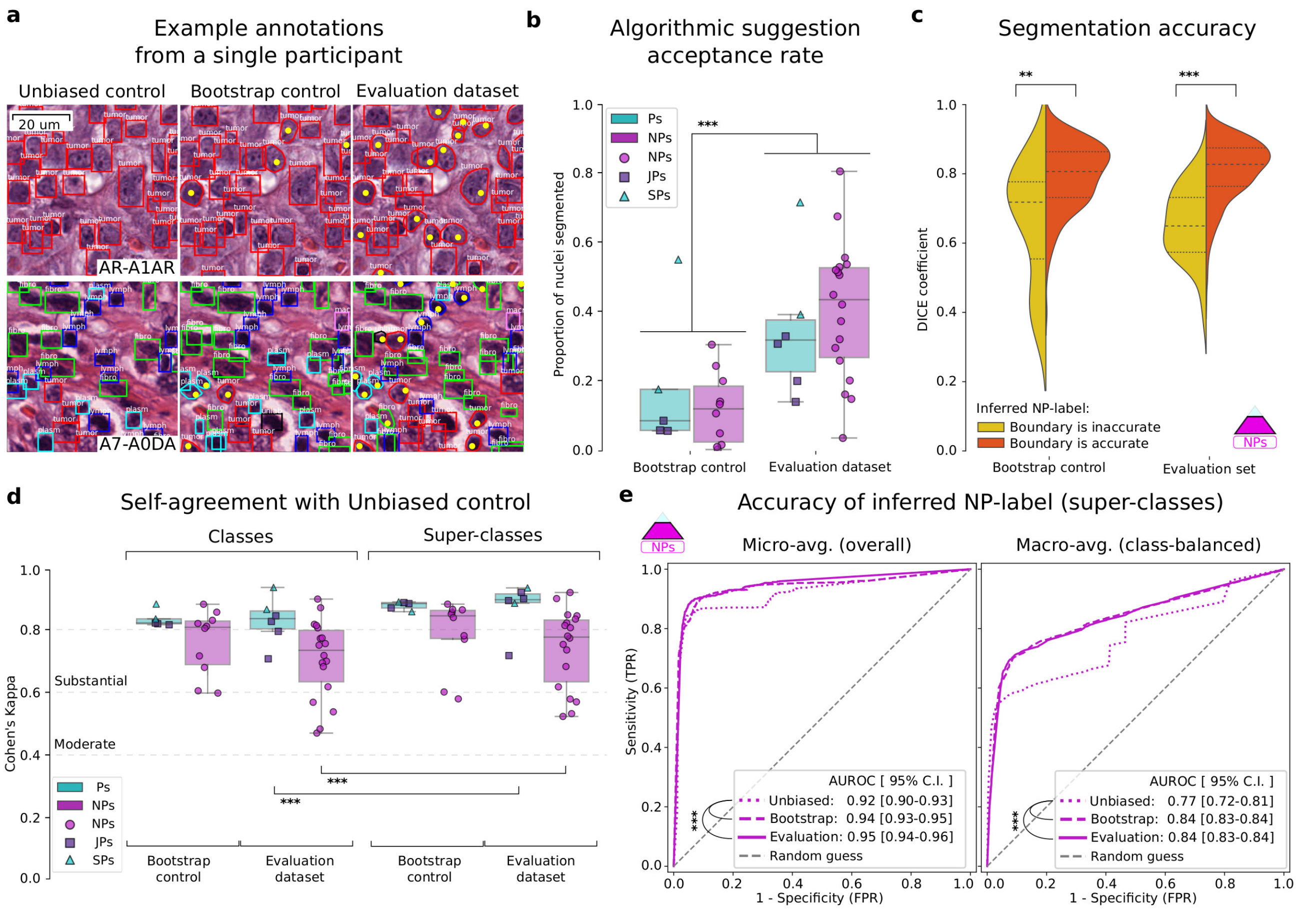

Figure 4. Effect of algorithmic suggestions on annotation abundance and accuracy. We compared annotations from the Evaluation dataset and controls to measure the impact of suggestions and Mask R-CNN refinement on the acquisition of nucleus segmentation data and the accuracy of annotations. a. Example annotations from a single participant. Algorithmic suggestions allow the collection of accurate nucleus segmentations without added effort. Yellow points indicate clicks to approve suggestions. b. The number of segmented nuclei clicked is significantly higher for the Evaluation dataset than for the Bootstrap control, indicating that refinement improves suggestion quality. c. Accuracy of algorithmic segmentation suggestions. The comparison is made against a limited set of manually traced segmentation boundaries obtained from one senior pathologist. Suggestions that were determined to be correct by the Expectation-Maximization procedure had significantly more accurate segmentation boundaries. d. Self-agreement for annotations in the presence or absence of algorithmic suggestions. The agreement is substantial for NP and pathologist groups, indicating that algorithmic suggestions do not impact classification decisions adversely. Pathologists have higher self-agreement and are less impressionable than NPs. e. ROC curves for the classification accuracy of inferred NP-label, using inferred P-truth as our reference. Statistically-significant comparisons are indicated with a star (**, p<0.01; ***, p<0.001).

Heuristic nucleus segmentation. We used simple image processing heuristics to obtain noisy nucleus segmentations [31]. Images were analyzed at scan magnification (40x) with the following steps: 1. Hematoxylin stain unmixing using the Macenko method [51]. 2. Gaussian smoothing followed by global Otsu thresholding to identify foreground nuclei pixels [52]. This step was done for each region class separately to increase robustness. We used a variance of two pixels for lymphocyte-rich regions and five pixels for other regions. 3. Connected-component analysis split the nuclei pixel mask using 8-connectivity and a 3x3 structuring element [53]. 4. We computed the Euclidean distance from every nucleus pixel to the nearest background pixel and found the peak local maxima using a minimum distance of 10 [54]. 5. A watershed segmentation algorithm split the connected components from step 3 into individual nuclei using the local maxima from step 4 as markers [55, 56]. 6. Any object < 300 pixels in area was removed.

Bootstrapping noisy training data. Region annotations were used to assign a noisy class to each segmented nucleus. This decision was based on the observation that although tissue regions usually contain multiple cell types, there is often a single predominant cell type: tumor regions / tumor cells, stromal regions / fibroblasts, lymphocytic infiltrate / lymphocytes, plasmacytic infiltrate / plasma cells, other regions / other cells. One exception to this direct mapping is stromal regions, which contain a large number of sTILs in addition to fibroblasts. Within stromal regions, a nucleus was considered a fibroblast if it had a spindle-like shape with an aspect ratio between 0.4 and 0.55 and circularity between 0.7 and 0.8.

Mask R-CNN refinement of bootstrapped suggestions. A Mask R-CNN model with a Resnet50 backbone was used as a function approximator to refine the bootstrapped nucleus suggestions. This model was trained using randomly cropped 128x128 tiles where the number of nuclei was limited to 30. Table S3 includes other hyperparameters.

FOV sampling procedure. ROI locations were carried over from the BCSS dataset. ROIs were manually selected by a medical doctor (M.A.), who served as a study coordinator for both the BCSS and NuCLS projects, and approved by a senior pathologist (H.E.). These ROIs were then tiled into non-overlapping potential FOVs, which were automatically selected for inclusion in our study based on predefined stratified sampling criteria. 16.7% of FOVs were sampled such that the majority of refined suggestions were a single class, e.g., almost all suggestions are tumor. 16.7% were sampled to favor FOVs with two almost equally-represented classes, e.g., many tumor and fibroblast suggestions. Finally, 16.7% of FOVs were sampled to favor discordance between the bootstrapped suggestions and Mask R-CNN-refined suggestions, e.g., a stromal region with sTILs. The remaining 50% of FOVs were randomly sampled from the following pool, with the intent of favoring the annotation of difficult nuclei:

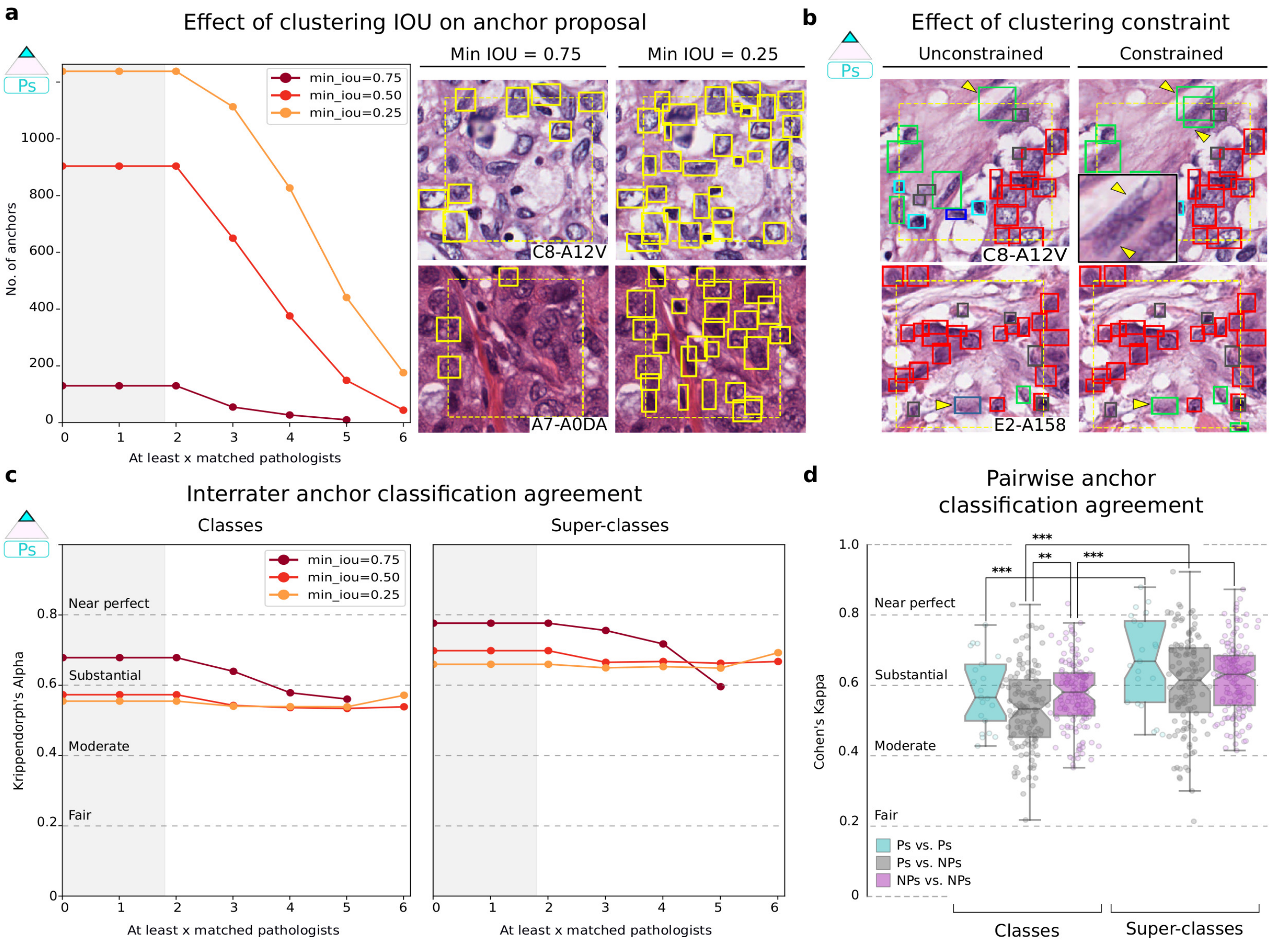

Figure 5. Effect of clustering on detection and interrater agreement. a. Stricter IOU thresholds reduce the number of anchor proposals generated by clustering but increase agreement. A threshold of 0.25 provides more anchor proposals with negligible difference in agreement from the 0.5 threshold. The shaded region indicates that by design, there are no anchor proposals with less than two clustered annotations. b. The clustering constraint prevents annotations from the same participant from being assigned to the same anchor, preserving participant intention when annotating overlapping nuclei. This results in better detection of overlapping nuclei during clustering (upper panel) and also impacts the inferred P-truth for anchors (bottom panel). c. Interrater classification agreement among pathologists for tested clustering thresholds. d. Pairwise interrater classification agreement (Cohen's Kappa) at 0.25 IOU threshold. Statistically-significant comparisons are indicated with a star (**, p<0.01; ***, p<0.001).

a) the bottom 5% of FOVs containing high numbers of nuclei with low Mask R-CNN confidence; b) and the top 5% of FOVs containing extreme size detections, presumably clumped nuclei.

Annotation procedure and data management

The annotation protocol used is provided in the supplement. We asked the participants to annotate the single-rater dataset first because this also acted as their de-facto training. Participants were blinded to the multi-rater dataset name to avoid biasing them. The Unbiased control was annotated first for the same reason. A summary of the data management procedure is provided below.

HistomicsUI. We used the Digital Slide Archive, a web-based data management tool, to assign slides and annotation tasks (digitalslidearchive.github.io) [45]. HistomicsUI, the associated annotation interface, was used for creating, correcting, and reviewing annotations. Using a centralized setup avoids participants installing software and simplifies the dissemination of images, control over view/edit permissions, monitoring progress, and collecting results. The annotation process is illustrated in this video. The process of pathologist review of annotations is illustrated in Figure S1.

HistomicsTK Application Programming Interface. The HistomicsTK Restful Application Programming Interface (API) was used to manage data, users, and annotations programmatically. This includes uploading algorithmic suggestions, downloading participant annotations, and scalable correction of systematic annotation errors where appropriate.

Obtaining labels from multi-rater datasets

Obtaining anchor proposals. We implemented a constrained agglomerative hierarchical clustering process to obtain anchor proposals (Figure 2a). The algorithm is summarized in Figure S10. In order to have a single frame of reference for comparison, annotations from all participants and for all multi-rater datasets were clustered. After clustering, we used two rules to decide which anchor proposals corresponded to actual nuclei (for each multi-rater dataset independently): 1. At least two pathologists must detect a nucleus. 2. The inferred P-truth must concur that the anchor is a nucleus.

Inference of NP-labels and P-truth. We used the Expectation-Maximization framework described by Dawid and Skene [46, 47, 57]. Each participant was assigned an initial quality score of 0.7, and 70 Expectation-Maximization iterations were performed. As illustrated in Figure 2b, undetected was considered a nucleus class for P-truth/NP-label inference. The same process was used to infer whether the boundary of an algorithmic suggestion was accurate. In effect, the segmentation accuracy was modeled as a binary vari-

able (clicked vs. not clicked), and the Expectation-Maximization procedure was applied to infer its value.

Class grouping

We defined two levels of grouping for nuclei classes as illustrated in Figure 2c. This was done for both the single-rater and multi-rater dataset annotations. Aggregate Expectation-Maximization probability was calculated by summing probabilities across subsets.

Participant agreement

Overall interrater agreement was measured using Krippendorff's alpha statistic, implemented in Python by Santiago Castro and Thomas Grill [58, 59, 60]. This statistic was chosen because of its ability to handle missing values [61]. Pairwise interrater agreement was measured using Cohen's Kappa statistic [62]. Likewise, self-agreement was measured using Cohen's Kappa. All of these measures range from -1 (perfect disagreement) to +1 (perfect agreement). A kappa (or alpha) value of zero represents agreement that is expected by random chance. We used thresholds set by Fleiss for defining slight, fair, moderate, substantial, and near-perfect agreement [61].

Annotation redundancy simulations

We performed simulations to measure the impact of the number of NPs assigned to each FOV on the accuracy of NP-label inference (Figure 3e). We kept the total number of NPs constant at 18 and randomly removed annotations to obtain a desired number of NPs per FOV. No constraints were placed on how many FOVs any single NP had. This simulated the realistic scenario where participants can annotate as many FOVs as they want, and our decision-making focuses on FOV assignment. For each random realization, we calculated the inferred NP-labels using Expectation-Maximization and measured accuracy against the static P-truth. This process was repeated for 1000 random realizations per configuration.

Software

Data management, machine learning models, and plotting were all implemented using Python 3+. Pytorch and Tensorflow libraries were used for various deep-learning experiments. Scikit-learn, Scikit-image, OpenCV, HistomicsTK, Scipy, Numpy, and Pandas libraries were used for matrix and image processing operations. Openslide library and HistomicsTK Application Programming Interface were used for interaction with whole-slide images.

Statistical tests

The Mann-Whitney U test was used for unpaired comparisons. The Wilcoxon signed-rank test was used for paired comparisons. Confidence bounds for the AUROC values were obtained by bootstrap sampling with replacement using 1000 trials [63, 64]. AUROC values are presented in the format: value[5th percentile, 95th percentile].

Conclusion

In summary, we have described a scalable crowdsourcing approach that benefits from the participation of NPs to reduce pathologist effort and enables minimal-effort collection of segmentation boundaries. We systematically examined aspects related to the interrater agreement and truth inference. There are important limitations and opportunities to improve on our work. Our results suggest that the participation of NPs can help address the scarcity of pathologists' availability, especially for repetitive annotation tasks. This benefit, however, is restricted to annotating predominant and visually distinctive patterns. Naturally, pathologist input — and possibly full-scale annotation effort- would be needed to supplement uncommon and challenging classes that require greater expertise. Some nuclear classes may be challenging to annotate in Hematoxylin and Eosin stained slides reliably and would be subject to considerable interrater variability even among practicing pathologists. In these settings, and where resources allow, IHC stains may be used as a more objective form of ground truth [65].

We chose to engage medical students and graduates with the presumption that familiarity with basic histology would help acquire higher-quality data. Whether this presumption was warranted or whether it was possible to engage a broader pool of participants was not investigated. On a related note, while we observed differences based on pathologist expertise, this was not our focus. We expect to address related questions such as the value of fellowship specialization in future work. Also, we did not measure the time it took participants to create annotations; we relied on the safe assumption that certain annotation types evidently take less time and effort than others.

Another limitation is that the initial bootstrapped nuclear boundaries were generated using classical image processing methods, which tend to underperform where nuclei are highly clumped/touching or have very faint staining. This theoretically introduces some bias in our dataset, with an overrepresentation of simpler nuclear boundaries. Future work could investigate the use of transfer learning or unsupervised convolutional neural network approaches to generate more accurate algorithmic suggestions. Similarly, we used Mask R-CNN as a function approximator to refine our algorithmic suggestions. Future research can explore other deep-learning architectures that may improve refinement and result in better algorithmic suggestions.

We focused our annotation efforts on nucleus detection, as opposed to whole cells. Nuclei have distinct staining (hematoxylin) and boundaries, potentially reducing the interrater variability associated with the detection of cell boundaries. Finally, we would point out that dataset curation is context-dependent and likely differs depending on the problem. Nevertheless, we trust that most of our conclusions have broad implications for other histopathology annotation efforts.

Availability of supporting data and materials

The NuCLS dataset is available at the NuCLS website. The BCSS dataset, which helped contribute to the algorithmic suggestions, is available for download from this Github repository, and can be viewed at this demo instance of the Digital Slide Archive. Both the BCSS and NuCLS datasets are available under a CC0 1.0 license. Snapshots of our code and other data further supporting this work are openly available in the GigaScience repository, GigaDB [66].

Availability of source code and requirements

Project name: NuCLS.
Project home page: github.com/PathologyDataScience/NuCLS.
Operating system(s): Platform independent.
Programming language: Python.
Other requirements: We used this tensorflow implementation by Matterport Inc. to train the Mask R-CNN tensorflow model used for generating the algorithmic suggestions, along with a set of scripts available on Github. We used the Digital Slide Archive for whole-slide image and data management (available here), its associated annotation user interface HistomicsUI (available here), as well as the annotation and image processing library HistomicsTK (here).

License: The NuCLS codebase is licensed with an CC0 1.0 license (dataset) and the MIT license.
Restrictions to use by non-academics: Both the CC0 1.0 license (dataset) and the MIT license (codebase) allow for non-commercial use. Please review license terms for details.
Registration: *RRID*: SCR_021888. *Biotools ID*: nucls.

Declarations

List of abbreviations

AP: Average Precision; **AUROC**: Area under Receiver-Operator Characteristic curve; **BCSS**: Breast Cancer Semantic Segmentation dataset; **FOV**: Field of view; **IOU**: Intersection over union; **JPs**: Junior Pathologists; **MCC**: Matthiew's Correlation Coefficient; **NPs**: Non-pathologists; **NP-label**: Inferred label from multi-rater pathologist data; **NuCLS**: Nucleus classification, localization, and segmentation; **Ps**: Junior or senior pathologists; **P-truth**: Inferred truth from multi-rater pathologist data; **ROI**: Region of Interest; **SPs**: Senior pathologists; **TCGA**: The Cancer Genome Atlas.

Ethical Approval

Not applicable.

Consent for publication

Not applicable.

Competing Interests

The author(s) declare that they have no competing interests.

Funding

This work was supported by the U.S. National Institutes of Health National Cancer Institute grants U01CA220401 and U24CA19436201. Lee A.D. Cooper is the Principal Investigator for the grants. The funding body had no role in the design of the study, data collection, data analysis, or data interpretation, or writing the manuscript.

Author's Contributions

M.A. and L.A.D.C. conceived the hypothesis, designed the experiments, performed the analysis, and wrote the manuscript. D.M. and D.A.G. contributed support for the Digital Slide Archive software and database. B.D. and D.J. provided ideas for the interrater analysis. M.A. and M.A.T.E. were the study coordinators and corrected the single-rater dataset. H.E. provided feedback and approved the corrected single-rater dataset. E.H. provided manual nucleus segmentation data. H.E., H.H., and E.H. are senior pathologists and provided multi-rater annotations. L.A.A., K.H.M., P.A.P., and L.E.H. are junior pathologists and provided multi-rater annotations. M.A.T.E., A.M.A., M.A.A., A.M.E., R.A.S., A.R., A.M.S., A.M.A., I.A.R., A.A., N.M.E., A.A., A.F., A.E., A.G.E., Y.A., Y.A.A., A.M.R., M.K.N., M.A.T.E., A.A., A.G., and M.E. are non-pathologists and provided single- and multi-rater annotations. All experience designations are based on the time of annotation. All authors reviewed the manuscript draft.

Acknowledgements

We would like to acknowledge with gratitude the contributions made by the following participants: Eman Elsayed Sakr (El-Matariya Teaching Hospital, Egypt), Joumana Ahmed (Cairo University, Egypt); Mohamed Zalabia and Ahmed S. Badr (Menoufia University, Egypt); Ahmed M. Afifi (Ain Shams University, Egypt); Esraa B. Ghadban (Damascus University, Syria); Mahmoud A. Hashim (Baylor College of Medicine, USA). In addition, we are thankful to Uday Kurkure, Jim Martin, Raghavan Venugopal, Joachim Schmidt (Roche Tissue Diagnostics, USA), and Michael Barnes (Roche Diagnostic Information Solutions, USA) for support and discussions. We also thank Brian Finkelman for constructive feedback on the interrater analysis. Finally, we thank Jeff Goldstein and other members of the Cooper research group at Northwestern for constructive feedback and discussion.

Full list of author affiliations

[1]Department of Pathology, Northwestern University, Chicago, IL, USA and [2]Cairo Health Care Administration, Egyptian Ministry of Health, Cairo, Egypt and [3]Department of Pathology, Nasser institute for research and treatment, Cairo, Egypt and [4]Department of Pathology and Laboratory Medicine, University of Pennsylvania, PA, USA and [5]Department of Clinical Laboratory Research, Theodor Bilharz Research Institute, Giza, Egypt and [6]Department of Medicine, Cook County Hospital, Chicago, IL, USA and [7]Department of Pathology, Baystate Medical Center, University of Massachusetts, Springfield, MA, USA and [8]Faculty of Medicine, Menoufia University, Menoufia, Egypt and [9]Faculty of Medicine, Al-Azhar University, Cairo, Egypt and [10]Consultant for The Center for Applied Proteomics and Molecular Medicine (CAPMM), George Mason University, Manassas, VA, USA and [11]Department of Pathology, National Liver Institute, Menoufia University, Menoufia, Egypt and [12]Faculty of Medicine, Ain Shams University, Cairo, Egypt and [13]Cleveland Clinic Foundation, Cleveland, OH, USA and [14]Department of Pathology, Indiana University, Indianapolis, IN, USA and [15]Faculty of Medicine, Damascus University, Damascus, Syria and [16]Faculty of Medicine, Mansoura University, Mansoura, Egypt and [17]Faculty of Medicine, Cairo University, Cairo, Egypt and [18]Department of Anaesthesia and Critical Care, Menoufia University Hospital, Menoufia, Egypt and [19]Department of Clinical Pathology, Ain Shams University, Cairo, Egypt and [20]Research Department, Oncology Consultants, PA, Houston, TX, USA and [21]Siparadigm Diagnostic Informatics, Pine Brook, NJ, USA and [22]Department of Pathology and Laboratory Medicine, Emory University School of Medicine, Atlanta, GA, USA and [23]Kitware Inc., Clifton Park, NY, USA and [24]Department of Neurology, Emory University School of Medicine, Atlanta, GA, USA and [25]Department of Pathology, National Cancer Institute, Cairo, Egypt and [26]Department of Pathology, Children's Cancer Hospital Egypt (CCHE 57357), Cairo, Egypt and [27]Lurie Cancer Center, Northwestern University, Chicago, IL, USA and [28]Center for Computational Imaging and Signal Analytics, Northwestern University Feinberg School of Medicine, Chicago, IL, USA

References

1. Litjens G, Kooi T, Bejnordi BE, Setio AAA, Ciompi F, Ghafoorian M, et al. A survey on deep learning in medical image analysis. Med Image Anal 2017 Dec;42:60–88.
2. Abels E, Pantanowitz L, Aeffner F, Zarella MD, Laak J, Bui MM, et al., Computational pathology definitions, best practices, and recommendations for regulatory guidance: a white paper from the Digital Pathology Association; 2019.
3. Hartman DJ, Van Der Laak JAWM, Gurcan MN, Pantanowitz L. Value of Public Challenges for the Development of Pathology Deep Learning Algorithms. J Pathol Inform 2020 Feb;11:7.
4. Amgad M, International Immuno-Oncology Biomarker Working Group, Stovgaard ES, Balslev E, Thagaard J, Chen W, et al., Report on computational assessment of Tumor Infiltrating Lymphocytes from the International Immuno-Oncology Biomarker Working Group; 2020.
5. Beck AH, Sangoi AR, Leung S, Marinelli RJ, Nielsen TO, van de Vijver MJ, et al. Systematic analysis of breast cancer morphology uncovers stromal features associated with survival. Sci Transl Med 2011 Nov;3(108):108ra113.
6. Koh PW, Nguyen T, Tang YS, Mussmann S, Pierson E, Kim B, et al.

Concept bottleneck models. In: International Conference on Machine Learning PMLR; 2020. p. 5338–5348.
7. Naik S, Doyle S, Agner S, Madabhushi A, Feldman M, Tomaszewski J, Automated gland and nuclei segmentation for grading of prostate and breast cancer histopathology; 2008.
8. Cooper LAD, Kong J, Gutman DA, Wang F, Gao J, Appin C, et al. Integrated morphologic analysis for the identification and characterization of disease subtypes. J Am Med Inform Assoc 2012 Mar;19(2):317–323.
9. Cooper LAD, Kong J, Gutman DA, Wang F, Cholleti SR, Pan TC, et al., An Integrative Approach for In Silico Glioma Research; 2010.
10. Alexander J Lazar, Michael D McLellan, Matthew H Bailey, Christopher A Miller, Elizabeth L Appelbaum, Matthew G Cordes, Catrina C Fronick, The Cancer Genome Atlas Research Network. Comprehensive and Integrated Genomic Characterization of Adult Soft Tissue Sarcomas. Cell 2017 Nov;171(4):950–965.e28.
11. Saltz J, Gupta R, Hou L, Kurc T, Singh P, Nguyen V, et al. Spatial Organization and Molecular Correlation of Tumor-Infiltrating Lymphocytes Using Deep Learning on Pathology Images. Cell Rep 2018 Apr;23(1):181–193.e7.
12. Diao JA, Wang JK, Chui WF, Mountain V, Gullapally SC, Srinivasan R, et al. Human-interpretable image features derived from densely mapped cancer pathology slides predict diverse molecular phenotypes. Nat Commun 2021 Mar;12(1):1613.
13. Lu W, Graham S, Bilal M, Rajpoot N, Minhas F. Capturing Cellular Topology in Multi-Gigapixel Pathology Images. In: Proceedings of the IEEE/CVF Conference on Computer Vision and Pattern Recognition Workshops; 2020. p. 260–261.
14. Alexander CB, Bruce Alexander C, Pathology graduate medical education (overview from 2006-2010); 2011.
15. Kovashka A, Russakovsky O, Fei-Fei L, Grauman K, Crowdsourcing in Computer Vision; 2016.
16. Ching T, Himmelstein DS, Beaulieu-Jones BK, Kalinin AA, Do BT, Way GP, et al. Opportunities and obstacles for deep learning in biology and medicine. J R Soc Interface 2018 Apr;15(141).
17. Amgad M, Man Kin Tsui M, Liptrott SJ, Shash E. Medical Student Research: An Integrated Mixed-Methods Systematic Review and Meta-Analysis. PLoS One 2015 Jun;10(6):e0127470.
18. Shaw S, Pajak M, Lisowska A, Tsaftaris SA, O'Neil AQ. Teacher-student chain for efficient semi-supervised histology image classification. arXiv preprint arXiv:200308797 2020;.
19. Hou L, Agarwal A, Samaras D, Kurc TM, Gupta RR, Saltz JH, Robust Histopathology Image Analysis: To Label or to Synthesize?; 2019.
20. Irshad H, Montaser-Kouhsari L, Waltz G, Bucur O, Nowak JA, Dong F, et al., Crowdsourcing image annotation for nucleus detection and segmentation in computational pathology: Evaluating experts, automated methods, and the crowd; 2014.
21. Campanella G, Hanna MG, Geneslaw L, Miraflor A, Werneck Krauss Silva V, Busam KJ, et al. Clinical-grade computational pathology using weakly supervised deep learning on whole slide images. Nat Med 2019 Aug;25(8):1301–1309.
22. Alemi Koohbanani N, Jahanifar M, Zamani Tajadin N, Rajpoot N. NuClick: A deep learning framework for interactive segmentation of microscopic images. Med Image Anal 2020 Oct;65:101771.
23. Deshpande S, Minhas F, Graham S, Rajpoot N. SAFRON: Stitching Across the Frontier for Generating Colorectal Cancer Histology Images. arXiv preprint arXiv:200804526 2020;.
24. Mahmood F, Borders D, Chen RJ, Mckay GN, Salimian KJ, Baras A, et al. Deep Adversarial Training for Multi-Organ Nuclei Segmentation in Histopathology Images. IEEE Trans Med Imaging 2020 Nov;39(11):3257–3267.
25. Koohbanani NA, Unnikrishnan B, Khurram SA, Krishnaswamy P, Rajpoot N. Self-Path: Self-supervision for Classification of Pathology Images with Limited Annotations. IEEE Transactions on Medical Imaging 2021;.
26. Ørting S, Doyle A, van Hilten A, Hirth M, Inel O, Madan CR, et al. A survey of crowdsourcing in medical image analysis. arXiv preprint arXiv:190209159 2019;.
27. Marzahl C, Aubreville M, Bertram CA, Gerlach S, Maier J, Voigt J, et al. Fooling the crowd with deep learning-based methods. arXiv preprint arXiv:191200142 2019;.
28. Amgad M, Elfandy H, Hussein H, Atteya LA, Elsebaie MAT, Abo Elnasr LS, et al. Structured crowdsourcing enables convolutional segmentation of histology images. Bioinformatics 2019 Sep;35(18):3461–3467.
29. Graham S, Vu QD, Raza SEA, Azam A, Tsang YW, Kwak JT, et al. Hover-Net: Simultaneous segmentation and classification of nuclei in multi-tissue histology images. Med Image Anal 2019 Dec;58:101563.
30. Kumar N, Verma R, Anand D, Zhou Y, Onder OF, Tsougenis E, et al. A Multi-Organ Nucleus Segmentation Challenge. IEEE Trans Med Imaging 2020 May;39(5):1380–1391.
31. Xing F, Yang L. Robust Nucleus/Cell Detection and Segmentation in Digital Pathology and Microscopy Images: A Comprehensive Review. IEEE Rev Biomed Eng 2016 Jan;9:234–263.
32. Gamper J, Koohbanani NA, Benet K, Khuram A, Rajpoot N, PanNuke: An Open Pan-Cancer Histology Dataset for Nuclei Instance Segmentation and Classification; 2019.
33. Gamper J, Koohbanani NA, Benes K, Graham S, Jahanifar M, Khurram SA, et al. Pannuke dataset extension, insights and baselines. arXiv preprint arXiv:200310778 2020;.
34. Veta M, Heng YJ, Stathonikos N, Bejnordi BE, Beca F, Wollmann T, et al. Predicting breast tumor proliferation from whole-slide images: The TUPAC16 challenge. Med Image Anal 2019 May;54:111–121.
35. Janowczyk A, Madabhushi A. Deep learning for digital pathology image analysis: A comprehensive tutorial with selected use cases. J Pathol Inform 2016 Jul;7:29.
36. Verma R, Kumar N, Patil A, Kurian NC, Rane S, Sethi A. Multi-organ nuclei segmentation and classification challenge 2020. IEEE Trans Med Imaging 2020;39:1380–1391.
37. Graham S, Jahanifar M, Azam A, Nimir M, Tsang YW, Dodd K, et al. Lizard: A Large-Scale Dataset for Colonic Nuclear Instance Segmentation and Classification. In: Proceedings of the IEEE/CVF International Conference on Computer Vision; 2021. p. 684–693.
38. Verma R, Kumar N, Patil A, Kurian NC, Rane S, Graham S, et al. MoNuSAC2020: A Multi-organ Nuclei Segmentation and Classification Challenge. IEEE Trans Med Imaging 2021 Jun;PP.
39. Dudgeon SN, Wen S, Hanna MG, Gupta R, Amgad M, Sheth M, et al. A pathologist-annotated dataset for validating artificial intelligence: a project description and pilot study. J Pathol Inform 2021;12:45.
40. Litjens G, Bandi P, Ehteshami Bejnordi B, Geessink O, Balkenhol M, Bult P, et al. 1399 H&E-stained sentinel lymph node sections of breast cancer patients: the CAMELYON dataset. Gigascience 2018 Jun;7(6).
41. Hou L, Gupta R, Van Arnam JS, Zhang Y, Sivalenka K, Samaras D, et al. Dataset of segmented nuclei in hematoxylin and eosin stained histopathology images of ten cancer types. Sci Data 2020 Jun;7(1):185.
42. Nalisnik M, Amgad M, Lee S, Halani SH, Velazquez Vega JE, Brat DJ, et al. Interactive phenotyping of large-scale histology imaging data with HistomicsML. Sci Rep 2017 Nov;7(1):14588.
43. Amgad M, Atteya L, Hussein H, Mohammed KH, Hafiz E, Elsebaie MAT, et al. Explainable nucleus classification using Decision Tree Approximation of Learned Embeddings. Bioinformatics 2021 Sep;.
44. He K, Gkioxari G, Dollár P, Girshick R. Mask r-cnn. In: Proceedings of the IEEE international conference on computer vision; 2017. p. 2961–2969.
45. Gutman DA, Khalilia M, Lee S, Nalisnik M, Mullen Z, Beezley J, et al. The Digital Slide Archive: A Software Platform for Management, Integration, and Analysis of Histology for Cancer Research. Cancer Res 2017 Nov;77(21):e75–e78.
46. Dawid AP, Skene AM, Maximum Likelihood Estimation of Observer Error-Rates Using the EM Algorithm; 1979.
47. Zheng Y, Li G, Li Y, Shan C, Cheng R, Truth inference in crowdsourcing; 2017.
48. Khoreva A, Benenson R, Hosang J, Hein M, Schiele B, Simple Does It: Weakly Supervised Instance and Semantic Segmentation; 2017.
49. Amgad M, Sarkar A, Srinivas C, Redman R, Ratra S, Bechert CJ, et al. Joint Region and Nucleus Segmentation for Characterization of Tumor Infiltrating Lymphocytes in Breast Cancer. Proc SPIE Int Soc Opt Eng 2019 Feb;10956.
50. Salgado R, Denkert C, Demaria S, Sirtaine N, Klauschen F, Pruneri G, et al., The evaluation of tumor-infiltrating lymphocytes (TILs) in breast cancer: recommendations by an International TILs Working Group 2014; 2015.
51. Macenko M, Niethammer M, Marron JS, Borland D, Woosley JT, Xiaojun Guan, et al. A method for normalizing histology slides for quantitative analysis. In: 2009 IEEE International Symposium on Biomedical Imaging: From Nano to Macro ieeexplore.ieee.org; 2009. p. 1107–1110.
52. Otsu N. A threshold selection method from gray-level histograms. IEEE Trans Syst Man Cybern 1979 Jan;9(1):62–66.

53. Gonzalez R, Woods R, Digital Image Processing, (March 1992). Addison-Wesley Publishing Company; 1992.
54. Maurer CR, Rensheng Qi, Raghavan V. A linear time algorithm for computing exact Euclidean distance transforms of binary images in arbitrary dimensions. IEEE Trans Pattern Anal Mach Intell 2003 Feb;25(2):265–270.
55. Beucher S. Use of watersheds in contour detection. In: Proceedings of the International Workshop on Image Processing; 1979. .
56. Soille PJ, Ansoult MM. Automated basin delineation from digital elevation models using mathematical morphology. Signal Processing 1990 Jun;20(2):171–182.
57. Zheng Y, Li G, Li Y, Shan C, Cheng R, Crowdsourcing truth inference (Github);. Accessed: 2020-12-19. https://github.com/zhydhkcws/crowd_truth_infer.
58. Krippendorff K. Krippendorff, Klaus, Content Analysis: An Introduction to its Methodology. Beverly Hills, CA: Sage, 1980 1980;.
59. Castro S, Fast Krippendorph;. Accessed: 2020-12-19. https://github.com/pln-fing-udelar/fast-krippendorff.
60. Grill T, Krippendorph alpha;. Accessed: 2020-12-19. https://github.com/grrrr/krippendorff-alpha.
61. Fleiss JL. Measuring nominal scale agreement among many raters. Psychol Bull 1971 Nov;76(5):378–382.
62. Cohen J. A Coefficient of Agreement for Nominal Scales. Educ Psychol Meas 1960 Apr;20(1):37–46.
63. Mann HB, Whitney DR. On a Test of Whether one of Two Random Variables is Stochastically Larger than the Other. Ann Math Stat 1947;18(1):50–60.
64. Wilcoxon F. Individual Comparisons by Ranking Methods. In: Kotz S, Johnson NL, editors. Breakthroughs in Statistics: Methodology and Distribution New York, NY: Springer New York; 1992.p. 196–202.
65. Tellez D, Balkenhol M, Otte-Holler I, van de Loo R, Vogels R, Bult P, et al., Whole-Slide Mitosis Detection in H&E Breast Histology Using PHH3 as a Reference to Train Distilled Stain-Invariant Convolutional Networks; 2018.
66. Amgad M, Atteya LA, Hussein H, Mohammed KH, Hafiz E, Elsebaie MA, et al., Supporting data for "NuCLS: A scalable crowdsourcing approach & dataset for nucleus classification and segmentation in breast cancer." GigaScience Database; 2022. http://dx.doi.org/10.5524/102207.

Click here to access/download
Supplementary Material
Supplementary
Material_Figure_AlgorithmicSuggestionAccuracy.eps

Click here to access/download
Supplementary Material
Supplementary Material_Figure_AnchorSummary.eps

Click here to access/download
Supplementary Material
Supplementary
Material_Figure_BootstrappingWorkflow.eps

Click here to access/download
Supplementary Material
Supplementary
Material_Figure_ClusteringAlgorithmBlock.eps

Click here to access/download
Supplementary Material
Supplementary
Material_Figure_HistomicsUIWorkflow.eps

Click here to access/download
Supplementary Material
Supplementary Material_Figure_ParticipAccuracy.eps

Click here to access/download
Supplementary Material
Supplementary Material_Figure_ParticipantConfusions.eps

Click here to access/download
Supplementary Material
Supplementary Material_Figure_PathologistReview.eps

Click here to access/download
Supplementary Material
Supplementary Material_Figure_PoorNPAnnotations.eps

Click here to access/download
Supplementary Material
Supplementary Material_Figure_SegmAccuracy.eps

Click here to access/download
Supplementary Material
Supplementary Material_IntegratedText.pdf
