# Supplementary material for: NuCLS: A scalable crowdsourcing approach and dataset for nucleus classification and segmentation in breast cancer
Source: Gigascience. 2022 May 17;11:giac037. doi: 10.1093/gigascience/giac037 (PMC9112766; doi:10.1093/gigascience/giac037)
Supplement: giac037_Supplemental_Files [file giac037_supplemental_files.zip › Supplementary Material_IntegratedText.pdf]

Supplementary material

NuCLS: A scalable crowdsourced approach & dataset for nucleus classification and segmentation in breast cancer

Mohamed Amgad[1], Lamees A. Atteya[2]*, Hagar Hussein[3]*, Kareem Hosny Mohammed[4]*, Ehab Hafiz[5]*, Maha A.T. Elsebaie[6]*, Ahmed M. Alhusseiny[7], Mohamed Atef AlMoslemany[8], Abdelmagid M. Elmatboly[9], Philip A. Pappalardo[10], Rokia Adel Sakr[11], Pooya Mobadersany[1], Ahmad Rachid[12], Anas M. Saad[13], Ahmad M. Alkashash[14], Inas A. Ruhban[15], Anas Alrefai[12], Nada M. Elgazar[16], Ali Abdulkarim[17], Abo-Alela Farag[12], Amira Etman[8], Ahmed G. Elsaeed[16], Yahya Alagha[17], Yomna A. Amer[8], Ahmed M. Raslan[18], Menatalla K. Nadim[19], Mai A.T. Elsebaie[12], Ahmed Ayad[20], Liza E. Hanna[3], Ahmed Gadallah[12], Mohamed Elkady[21], Bradley Drumheller[22], David Jaye[22], David Manthey[23], David A. Gutman[24], Habiba Elfandy[25, 26], Lee A.D. Cooper[1, 27, 28] **

* These authors contributed equally

**** The full list of affiliations can be found in the main manuscript text**

Supplementary tables

Table S1. Definitions and abbreviations used. The following paper from the Digital Pathology Association can be consulted for an expanded list of relevant concepts: *Abels, E. et al., The Journal of Pathology. 2019. 249: 286–294.*

Term	Abbr.	Definition
**Basic definitions**		
Whole slide image	WSI	High-resolution scanned image of a histopathology slide. Most WSIs of solid tumors are scanned at a 20-40x magnification and are extremely large (~80k pixels side)
Annotation	-	Manual markup of the image to indicate the location, boundary, or class of an anatomical structure. Examples include a point at the centroid of a nucleus, a bounding box indicating the extent of a nucleus, or tracing the nucleus boundary.
Segmentation	-	A boundary delineating the edge of a structure like a histologic region or a nucleus.
Ground truth	-	The true location/boundary/class of a particular nucleus. This term is used loosely in this paper to refer to the truth against which the deep-learning models are evaluated. This truth will be different under different circumstances, depending on the experiment being discussed.
Region of interest	ROI	A ~1 mm^2 region of a WSI from which FOVs are selected. Each ROI is accompanied by low-power annotations of tissue regions used for generating suggestions.
Field of view	FOV	A ~65 x 65 μm field selected from within an ROI. FOVs were annotated at high power to indicate the location and class of all nuclei contained in the FOV.
Application Programming Interface	API	A set of functions that allow developers to interact with a database or other software programmatically.
**Participant groups**		
Non-pathologists	NPs	Medical students/graduates who did not receive pathology residency training.
Junior pathologists	JPs	Pathology residents with < 2 years of anatomical pathology training.
Senior pathologists	SPs	Attendings or pathology residents with > 2 years of anatomical pathology training.
Pathologists	Ps	Junior or senior pathologists.
**Datasets**		
Hybrid dataset	-	A dataset where participants click accurate segmentation boundary suggestions and draw bounding boxes around all other nuclei. The resultant dataset contains a mixture of segmentation boundaries and bounding boxes.
Single-rater dataset	-	A collection of FOVs that NPs annotated in a single-rater manner. NPs received pathologist feedback during the annotation process. NPs were shown both region (low-power) and nucleus (high-power) suggestions while annotating.
Corrected single-rater dataset	-	A subset of single-rater dataset FOVs (approximately half) whose annotations have been manually corrected by study coordinators based on feedback from a senior pathologist. A senior pathologist approved all corrected single-rater dataset annotations.
Uncorrected single-rater dataset	-	Single-rater dataset FOVs whose annotations were not manually corrected. The quality of these annotations is participant-dependent.
Multi-rater datasets	-	A collection of FOVs that were annotated by multiple participants under different experimental conditions. NPs were not given feedback on these FOVs. These are used for interrater comparisons.

Evaluation dataset	-	A multi-rater dataset where Mask R-CNN refined algorithmic suggestions were shown to the participants. Refinement was applied to bootstrap suggestions to improve quality. These suggestions were the same type used in single-rater dataset annotation.
Bootstrap control	-	A multi-rater dataset where noisy bootstrapped algorithmic suggestions were shown to the participants. These suggestions were generated using a heuristic segmentation algorithm and processing of low power region annotations and shape data from segmentation.
Unbiased control	-	A multi-rater dataset where no annotation suggestions were shown to the participants. This was the first multi-rater dataset annotated to obtain annotations not biased by algorithmic suggestions.
**Nucleus suggestions and labels**		
Bootstrapped suggestions	-	A set of noisy nuclear boundary suggestions using simple image processing heuristics. Each boundary also had an associated classification suggestion, inherited from the histologic region where the presumed nucleus resides. Thus, for example, a suggested boundary in a tumor region would be associated with a tumor classification suggestion. These were an intermediate step in producing refined suggestions (see below) and were only shown to participants for the Bootstrap control dataset.
Mask R-CNN refined suggestions	-	The result of fitting a Mask R-CNN model to the bootstrap suggestion. Mask R-CNN acts as a function approximator to smooth out noise. These were shown to participants for the single-rater and Evaluation datasets.
Label	-	This term is used in the broad sense, as in *labeled data* used for supervised machine learning. A label is a tag associated with a potential nucleus location (anchor proposal, defined below). Labels include assessing whether an anchor proposal corresponds to a nucleus (i.e., detection), what class to assign (e.g., tumor) and whether or not the suggested segmentation boundary is correct.
Anchor proposal	-	A *potential* bounding box location of a nucleus. Anchor proposals are generated by clustering annotations from multi-rater datasets.
Class	-	A type of label that assigns a nucleus to a set of predefined biological categories (e.g., tumor, fibroblast, and TILs).
Raw nucleus classes	-	The set of 12 nucleus classes that were directly obtained from the participants, without class grouping.
Nucleus classes	-	A set of 7 nucleus classes, obtained by grouping related raw classes together.
Nucleus super-classes	-	Three clinically salient nucleus classes (tumor, stroma, sTILs), obtained by grouping nucleus classes.
Uncommon nucleus classes	-	Any raw nucleus classes other than tumor, fibroblasts, and lymphocytes.
Inferred pathologist truth	P-truth	A single label is generated from the analysis of multi-rater datasets using pathologist annotations. For each anchor proposal from clustering, we use EM to infer whether the proposal is an actual nucleus, the class, and the correctness of the suggested boundary. This was used to measure the accuracy of NP annotations and NP-label.
Inferred non-pathologist label	NP-label	A single label is generated from the analysis of multi-rater datasets using NP annotations (see inferred P-truth for comparison).
**Machine learning and image processing**		
Convolutional neural network	CNN	A deep-learning model that operates on image data.
Mask R-CNN	-	A CNN model that learns to jointly predict nucleus bounding-box localization, segmentation, and class.

Agglomerative hierarchical clustering	-	A bottom-up clustering approach that builds a hierarchy of clusters starting with each data point as its own cluster and grouping data points and clusters by similarity.
Expectation-Maximization	EM	An iterative method for estimating the parameters of a statistical model by maximizing a *likelihood* measure. It was used to simultaneously estimate participant reliability and nucleus locations, class, and correctness of boundaries.
Heuristic nucleus segmentation	-	Delineation of nuclear boundaries using simple image processing operations that have no dependence on annotation data (unlike machine learning models). This was used to generate bootstrapped algorithmic suggestions or segmentation boundaries.
**Measures of accuracy and agreement**		
Intersection over union	IOU	A quantitative measure of overlap of prediction and truth.
DICE coefficient	-	Similar to IOU, it is a measure of overlap of prediction and truth.
Area under Receiver-Operator Characteristic (ROC) curve	AUROC	It is a measure of accuracy, where a value of 0.5 corresponds to random chance, and a value of 1.0 is the maximum. There are two ways of obtaining this value:<br>- *Micro-average:* This is the overall accuracy, where different nucleus classes contribute to the result in proportion to their abundance in the dataset<br>- *Macro-average:* Is the class-balanced accuracy, where different nucleus classes are equally weighted, such that an uncommon class like macrophages will have the same contribution as a common class like sTILs.
Average precision	AP	The area under the precision-recall curve is used to measure detection performance. AP@.5 refers to the area measured with a minimum IOU of 0.5 for defining correct detections. mAP@.5:.95 is a more stringent measure that averages areas for a range of IOU thresholds from 0.5 to 0.95.
F1 score	-	The harmonic mean of precision and recall values.
Matthiew's Correlation Coefficient	MCC	A balanced measure of classification accuracy considers all components of the confusion matrix, including true negatives (unlike the F1 score).
Cohen's Kappa statistic	-	A measure of agreement between two participants, ranging from -1 (perfect disagreement) to +1 (perfect agreement).
Krippendorff's Alpha statistic	-	A multi-rater generalization of Cohen's Kappa, which handles missing values.

Table S2. Accuracy of algorithmic suggestions. The accuracy is measured against the corrected single-rater dataset. Mask R-CNN refinement of the bootstrapped algorithmic suggestions results in better detection suggestions. Low-power region-based classification was more accurate than Mask R-CNN-derived classes. Note, however, that this was FOV-dependent, and there were some FOVs in which the Mask R-CNN prediction was better than relying on low-power regions for classification.

Stage	Class		N	Accuracy	MCC	F1	Precision	Sensitivity	Specificity
Bootstrap suggestions	Detection		58598	**18.8**	-	**31.7**	**40.4**	**26.1**	-
	Classification (region-inherited)	**Overall**	11029	**86.8**	**77.9**	-	-	-	-
		Tumor		95.6	91.2	95.2	93.6	96.8	94.7
		Stromal		90.3	21.5	12.4	80.0	6.7	99.8
		sTILs		89.6	80.3	89.2	82.6	97.0	83.7
		Other		98.2	16.8	17.7	16.9	18.6	99.0
Suggestions after Mask R-CNN refinement	Detection		75908	**31.5**	-	**47.9**	**47.6**	**48.1**	-
	Classification (region-inherited)	**Overall**	23874	**78.9**	**67.6**	-	-	-	-
		Tumor		93.5	85.9	91.1	90.1	92.1	94.2
		Stromal		82.0	46.1	57.2	53.4	61.6	87.0
		sTILs		83.6	66.4	80.2	84.2	76.6	88.9
		Other		99.3	30.5	25.9	56.0	16.9	99.9
	Classification (Mask R-CNN prediction)	**Overall**		**69.1**	**52.7**	-	-	-	-
		Tumor		83.2	63.0	74.9	82.1	68.8	91.4
		Stromal		82.2	26.3	17.5	91.3	9.6	99.8
		sTILs		75.5	58.1	77.4	64.5	96.8	59.0
		Other		97.9	12.0	11.9	8.5	19.9	98.5

Table S3. Hyperparameters used for Mask R-CNN model training.

**Backbone**	Resnet50
**Pretraining**	Imagenet
**Input (cropped) image size**	128 x 128
**Max. ground truth nuclei per image**	30
**Max. detections per image (inference)**	200
**Batch size**	8
**Optimizer**	SGD
**Learning rate**	1.00E-04
**Momentum**	9.00E-01
**Length of anchor sides in pixels**	8,16,32,64,128
**ROIs after NMS (training)**	500
**ROIs after NMS (inference)**	1000
**NMS threshold for RPN proposals**	0.7

Supplementary figures

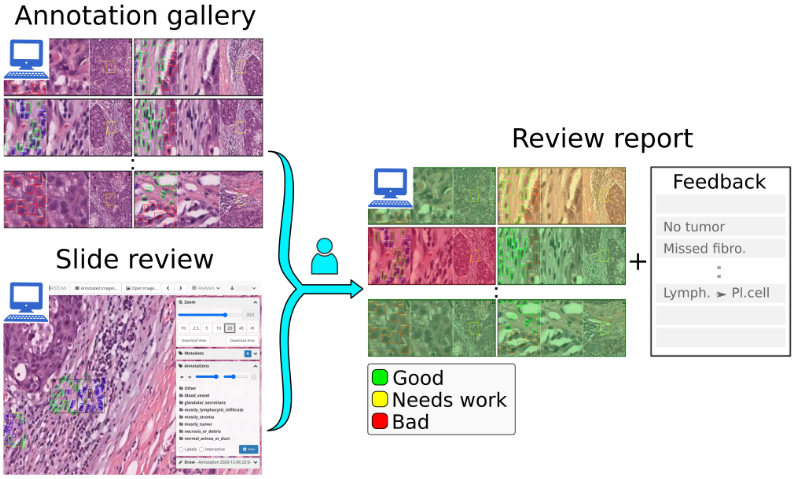

Figure S1. Use of review galleries for scalable review of single-rater annotations. Single-rater annotations were corrected by two study coordinators, in consultation with a senior pathologist. The pathologist was provided with a mosaic review gallery showing a bird's eye view of each FOV, with and without annotations, and at high and low power. The pathologist was asked to assign a per-FOV quality assessment. If the pathologist wanted further context, they were able to click on the FOV and pan around the full whole-slide image. They were also able to provide brief comments to be addressed by the coordinators, for eg. "change all to tumor". A demo is provided at the following video: https://youtu.be/Plh39obBg_0 .

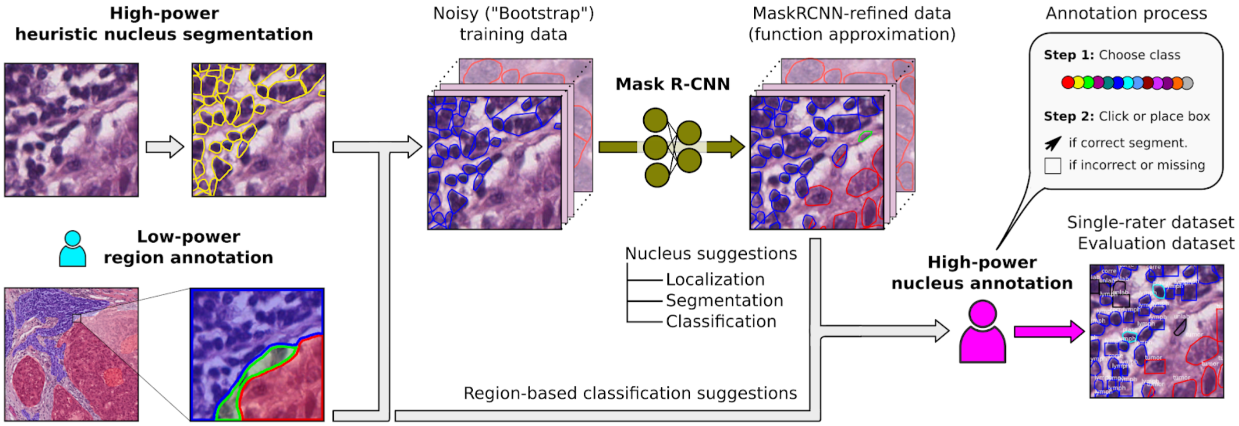

Figure S2. Process for obtaining algorithmic suggestions for scalable assisted annotation. Nucleus segmentation boundaries were derived using image processing heuristics at a high magnification. Low-power region annotations from the BCSS dataset, approved by a practicing pathologist, were then used to assign an initial class to nuclei. This combination of noisy nuclear segmentation boundaries and region-derived classifications are the *bootstrapped* suggestions. These noisy algorithmic suggestions were the basis for annotating the Bootstrap control multi-rater dataset. A Mask R-CNN model was then used as a function approximator to smooth out some of the noise in the bootstrapped suggestions. Participants were able to view these refined suggestions, along with low-power region annotations, when annotating the single-rater and Evaluation datasets.

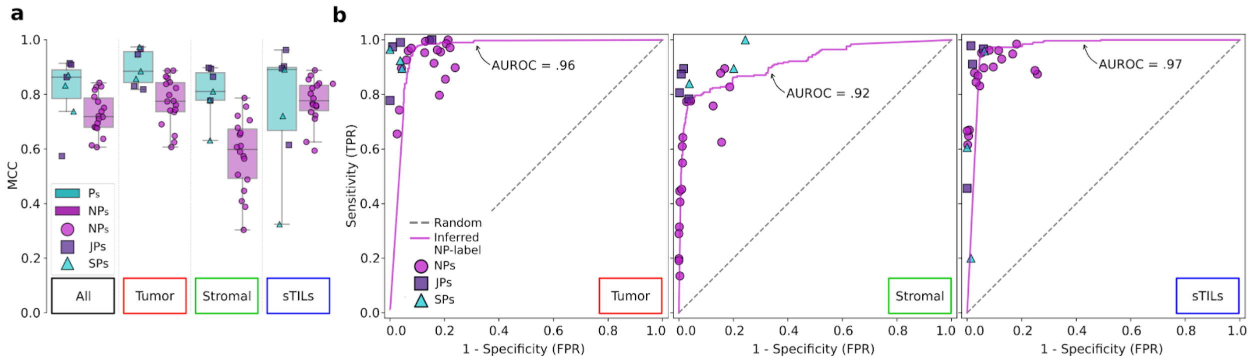

Figure S3. Super-class accuracy of participant annotations and inferred NP-labels (Evaluation dataset). The accuracy is measured against the inferred P-truth.

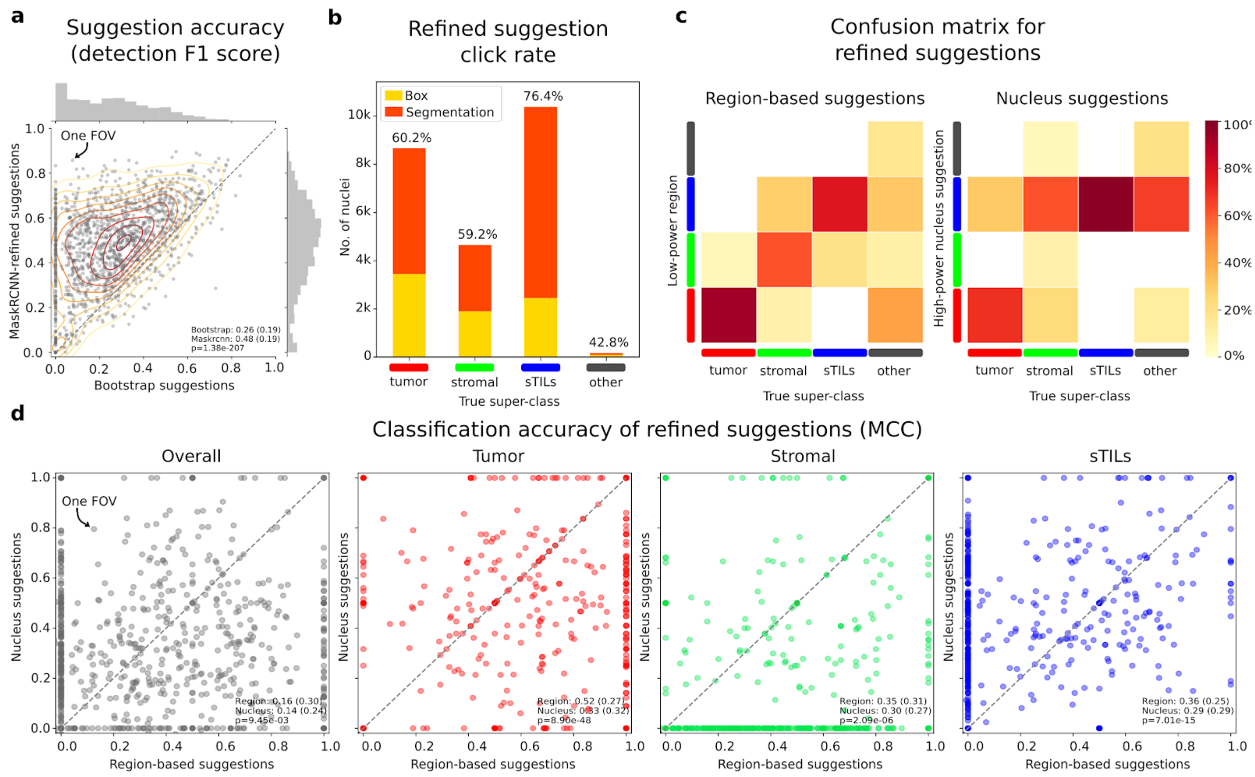

Figure S4. Accuracy of algorithmic suggestions (single-rater dataset). The accuracy is measured against the corrected single-rater dataset. **a.** Per-FOV detection accuracy of algorithmic data at the two stages of obtaining algorithmic suggestions; i.e. how well do the suggestions correspond to real nuclei? Mask R-CNN refinement significantly improves suggestion accuracy. **b**. Number of Mask R-CNN-refined suggestions that correspond to a segmentation (i.e. were clicked) or a bounding box. **c.** Concordance between suggested classes and classes assigned by participants. Region-based suggestions were, broadly-speaking, more concordant with the true classes, but nucleus suggestions had a higher recall for sTILs. **d.** Comparison of the classification accuracy (MCC) of low-power region class and high-power Mask R-CNN-derived nucleus class. Numbers are normalized column-wise, i.e. represent percentages of true nuclei of a particular class. Note how region-based and nucleus-based suggestions have disparate accuracies for different FOVs and classes. Hence, there was value in providing the participants with both forms of suggestion.

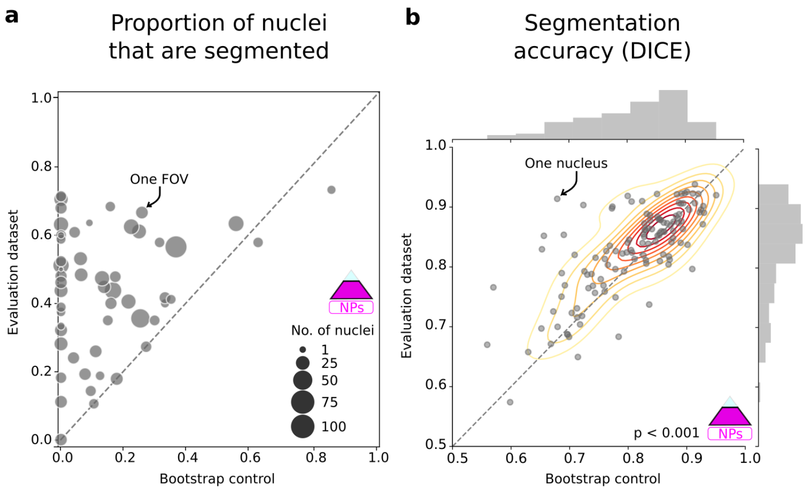

Figure S5. Abundance and segmentation accuracy of clicked algorithmic suggestions (multi-rater datasets). a. Proportion of nuclei in the FOV that were inferred to have good segmentation. Circle size represents the number of nuclei in that FOV. The proportion is notably higher for the Evaluation dataset than the Bootstrap control. **b.** Accuracy of algorithmic segmentation boundaries for nuclei that were inferred to have accurate segmentation boundaries in both the Evaluation dataset and Bootstrap control. The comparison is made against manual segmentations obtained for the same nuclei from one senior pathologist. Most clicked algorithmic segmentations were very accurate, and have a DICE coefficient above 0.8. The accuracy was slightly higher for Mask R-CNN-refined suggestions.

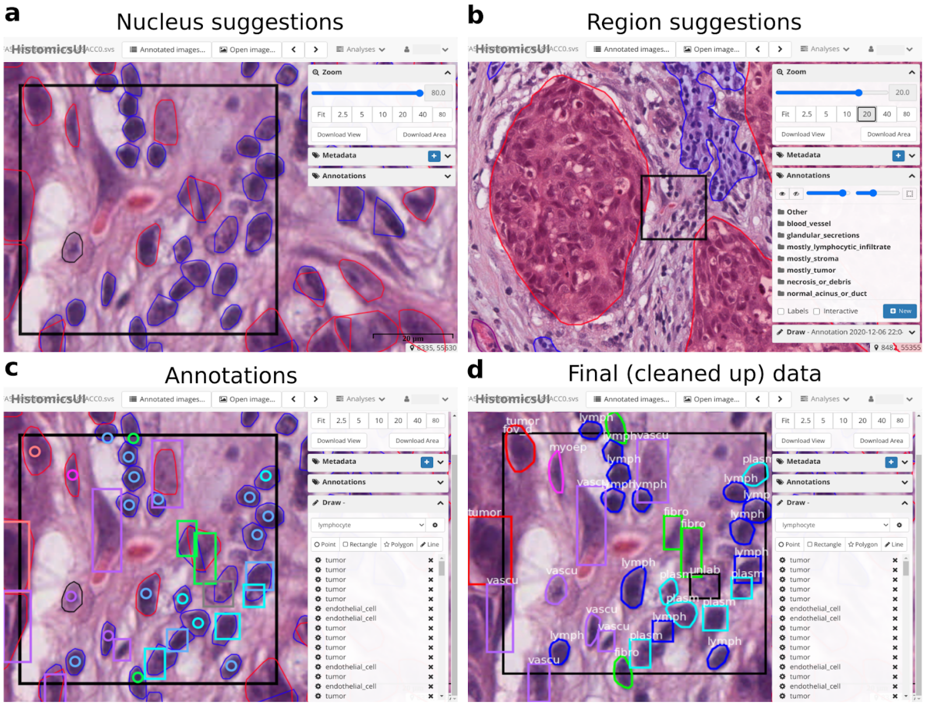

Figure S6. Annotation procedure on HistomicsUI. a-b. Participants were shown suggestions for nucleus segmentation boundaries, as well as two types of classification suggestions: low-power region suggestions and high-power nucleus classification suggestions. The FOV shown here is almost entirely present in a stromal region, but contains multiple scattered sTILs that were not dense enough to be captured as a sTILs “region”. **c.** Participants’ annotations were either points/clicks, for accurate segmentations, or bounding boxes. They picked the color/class of their annotations beforehand, and were told to simply ignore any inaccurate suggestions. Participants were able to turn the suggestions off for a clear view of the underlying tissue. **d.** Participant annotations and algorithmic suggestions were ingested into a database and processed to provide cleaned up data, which was then pushed for viewing on HistomicsUI for correction and review.

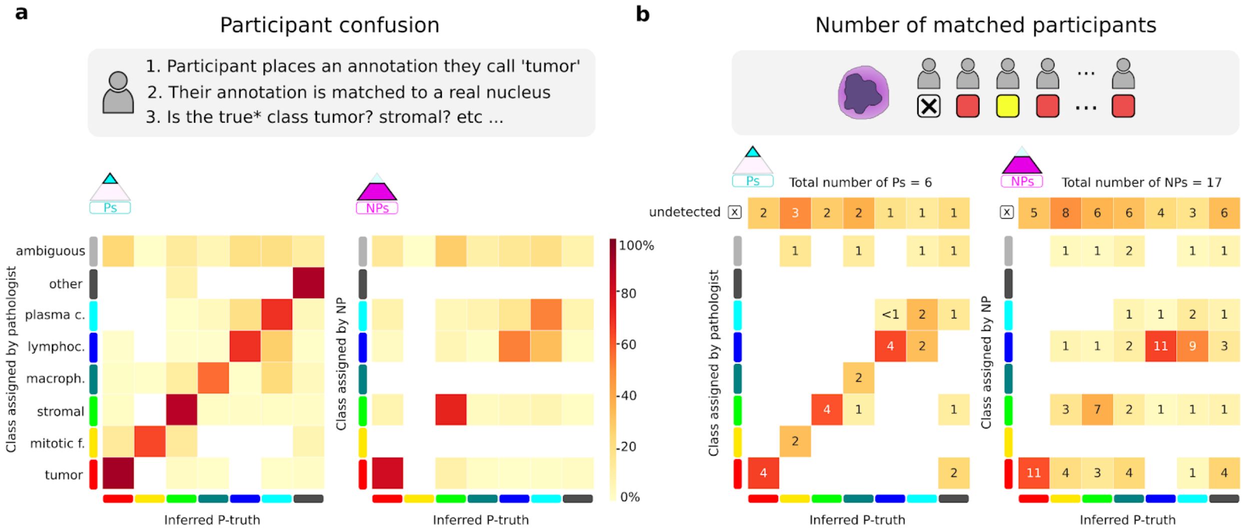

Figure S7. Confusion matrix of participant annotations (Evaluation dataset). a. Confusion of annotations placed by the participants, putting aside detection. Here, we ask the question, if a participant places an annotation they call tumor, and it matches a true nucleus, what is the class of that nucleus? By definition, there are no “ambiguous” true nuclei. **b.** For each true nucleus, how many of the participants detected it, and if so, what class did they assign? Note that since truth inference takes participant reliability into account, the inferred P-truth does not have to correspond to the most commonly assigned class. Empty entries are values <1.

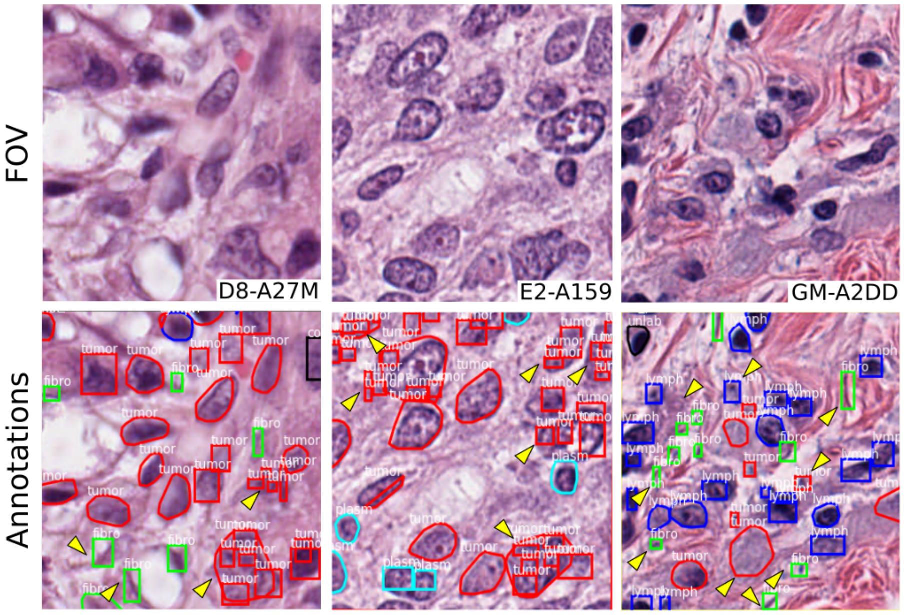

Figure S8. Sample poor annotation data excluded during the single-rater dataset correction process. Despite having received initial training and feedback, the NP who generated these annotations was confused about what is a nucleus, and frequently considered chromatin clumps or artifacts as nuclei (arrows). This underlines the need for quality control.

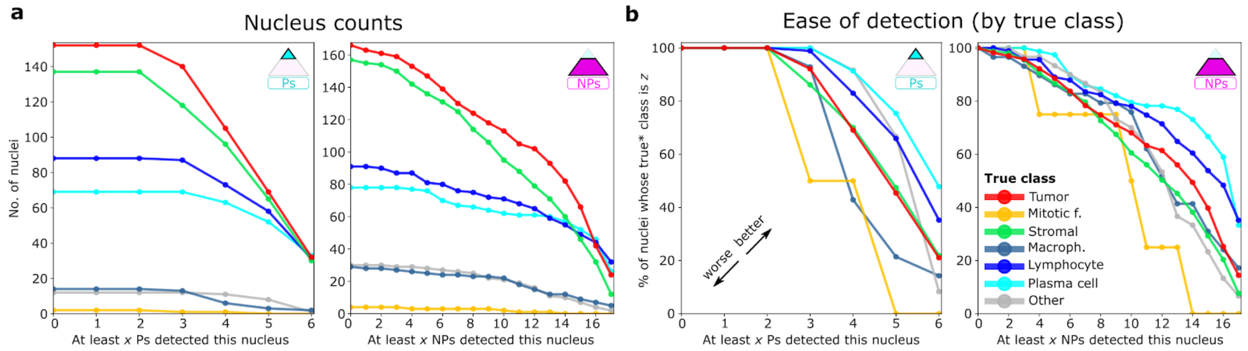

Figure S9. Ease of detection of various nucleus classes (Evaluation dataset). If we know for a fact this is, say, a lymphocyte, how many participants detected it, even if they called it something else?. True class is the inferred P-truth. The color coding used is explained in panel b. **a.** Nuclei counts, broken down by class and the number of matched participants. **b.** Ease of detection of nuclei by true class. Interpreting, say, the blue curve goes like this: 100% of lymphocytes were detected by at least 3 pathologists, ~80% were detected by 4 pathologists, and so on.

1 Do unconstrained agglomerative clustering with maximum linkage using bounding boxes from participants $\{P_1, P_2, \ldots\}$

2 Cut at linkage threshold $1 - t^*$ (where t^* is the threshold IOU)

3 For each cluster C_i (corresponding to top-level node N_i)

3.1 For each "don't-link" set S_j

3.1.1 Check if more than one member in S_j is present in C_i

3.1.2 For each extra member S_{jk} in C_i

3.1.2.1 Check the next low-level node N_{i-1}

> If there are no members from S_j in N_{i-1} :

- If C_{i-1} does not exist :

→ Set N_{i-1} as a new cluster C_{i-1}

- Assign S_{jk} to C_{i-1}

> Else :

- Check the next low-level node N_{i-2} (repeat 3.1.2.1)

3.1.2.2 If no nodes without members from S_j found :

> Assign S_{jk} as a separate one-leaf cluster

4 For each cluster C_i

4.1 Find IOU of bounding boxes of members $\{C_{i1}, C_{i2}, \ldots, C_{iN}\}$

4.2 Assign member $C_{im} =$ argmax(mean IOU) as the medoid

Figure S10. Algorithm for obtaining anchor proposals through constrained agglomerative clustering. We cluster bounding boxes from participants to get the *anchor proposals* corresponding to potential nucleus locations. Note that the threshold we use for maximum linkage, t*, is influential in determining how many anchors we get. We make sure that annotations from the same participant do not end up in the same cluster by creating sets of “don't-link” bounding boxes. The final anchor proposals are the anchor medoids; using medoids ensures that the box anchor proposals correspond to real nucleus boundaries.

Supplementary file: Annotation protocol

Welcome to the breast cancer nucleus annotation project! The purpose of this project is to investigate a scalable data collection and refinement procedure, and to create a large-scale dataset for training and validation of machine learning algorithms.

> Please view the introductory video before diving into this document.
> Please read this document in its entirety before making annotations.

There are three categories of participants:

- **NP (Non-pathologist)** - Did not receive anatomical pathology residency training.
- **JP (Junior pathologist)** - Pathology residents with < 2 years of training.
- **SP (Senior pathologist)** - Attendings or pathology residents with > 2 years of training.

There are two required annotation assignments for each NP:

- **Single-rater dataset:** You can ask questions and receive feedback from pathologists.
- **Multi-rater datasets:** No feedback will be provided. Annotate to the best of your ability.

> General remarks:

- Use a **comfortable mouse, table and monitor**. This greatly impacts comfort and quality.
- When in doubt, take a screenshot and post a question on **Slack** for review & feedback.
- Remember, the algorithm is **learning** what we teach it (Garbage In → Garbage Out).

> Annotation workflow:

- **Step 1:** View the **region-level annotations.** These are the low-power classification suggestions.
- **Step 2:** Go to **medium power** (20x) and **reduce transparency**. Examine the underlying tissue.
- **Step 3:** Zoom on the FOV at **maximum power** (40x or 80x, depending on slide).
- **Step 4:** **Start annotating**. The process is illustrated in the introductory video. Briefly, the steps are:

  > Pick an annotation class/color (feel free to rely on or ignore algorithmic suggestions)

  > If an algorithmic boundary is correct, place a dot.

  > Otherwise, place a bounding box around the nucleus.

> Specific annotation rules:

- Only annotate the Fields-of-View (FOVs) that were picked for you.
- If a nucleus extends beyond the FOV boundary, make sure your bounding box covers its full extent (i.e. extend your rectangle outside the FOV as well).
- Make sure each FOV is complete before moving to the next. Missing annotations may confuse our algorithms and make validation difficult!
- Make sure to annotate **in this order**: **Single-rater dataset → Multi-rater dataset 1 → Multi-rater dataset 2 and/or Multi-rater dataset 3.** SPs and JPs do not have a single-rater dataset (but they *do* have multi-rater datasets). Pathologists are kindly asked to respond to questions on Slack.

Explanatory note: *We asked the participants to annotate the single-rater dataset first because this also acted as their de-facto training, and they received feedback and could ask questions. The multi-rater datasets were blinded to avoid biasing the participants. Multi-rater dataset 1 is the unbiased control dataset (no algorithmic suggestions), and was annotated first for the same reason.*

- After you annotate your **first FOV**, take a screenshot and share it on the **Slack group** to get **approval & feedback before continuing.** This acts as a test of your understanding.
- After every slide in the single-rater dataset, please ask for feedback from the SPs and/or study coordinator. Do not post a screenshot of every single FOV, simply post the slide ID on the group and SPs/coordinator will go to the slide and make suggestions/corrections where necessary.
- Share a screenshot of anything that you are unsure of, making sure to also share the slide name so that the SPs and study coordinators can take a closer look at various magnifications. Nuclei are often vague. If you are unsure about the class of a nucleus, either:
    - Ask what it is on the group and receive feedback from SP (preferred).
    - Assign is the class *unlabeled*.

Make as much effort to classify nuclei as possible; only use the *unlabeled* class in a minority of cases.

- Make sure the **bounding box** is **tight** around the nucleus **NOT** the entire cell.
- Do not trust the computer suggestions too much. If the algorithmic boundaries are just slightly off then it's OK, otherwise use a bounding box instead.
- Never rotate the slide before annotating. All boxes should have the same orientation as the FOV.

> Notes about specific annotation classes:

Notes that address some frequently asked questions on the Slack group.

- **Tumor:** Malignant cells are very heterogeneous in shape. They tend to have hyperchromatic, eccentric nuclei, and tend to be crowded and irregular. See any standard pathology textbook.
- **Fibroblasts:** Stromal nuclei tend to be elongated and shaped like a cigar. May also have a rounder shape. The tell-tale sign is their presence in stroma in alignment with the collagen fibres. Some fibroblasts close to the tumor may be **activated** (i.e. have tumor-like morphology).
- **Lymphocytes:** Small, round, condensed, central nucleus. Tend to be grouped together.
- **Plasma cells:** May confuse with lymphocytes. Plasma cells are less common than lymphocytes; when in doubt, ask on Slack. They tend to have an eccentric, large, textured nucleus (described as *cart-wheel*, but rarely seen as such) with a pale perinuclear halo. Also tend to have eosinophilic cytoplasm
- **Macrophages:** Usually difficult to ascertain. They tend to be larger than lymphocytes, sometimes have vacuolated or frothy cytoplasm, have thin round-to-uniform (bean shaped) nuclei with variable nucleoli.

> Troubleshooting:

#	Situation	How to handle	Examples
**1**	Annotations take a long time to load.	- Close all programs running in the background.<br>- Close all other Google Chrome tabs, especially videos (except this document, which you always have to refer to)<br>- Switch from tablet to computer<br>- Switch to a faster internet connection	N/A
**2**	Algorithm correctly predicts both nucleus boundary and class	Place a dot inside the nucleus with the correct class	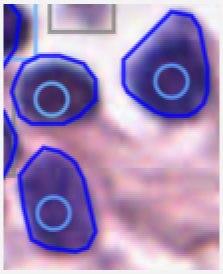
**3a**	Algorithm correctly predicts nucleus boundary but assigns incorrect class, and you know the correct class	- If you know correct class: Place a dot with correct class inside the nucleus	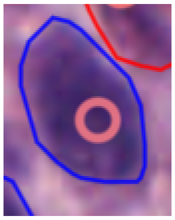

**3b**	Same as 3a, but you do not know the correct class	- If you are new to this or are in doubt, take a snapshot and ask for pathologist feedback.<br>- If you are confident in your ability (eg you have been annotating many FOVs or are a pathologist), i.e. the nucleus is vague and cannot be classified using just H&E: place a dot with the class *unlabeled*.	
**4**	Algorithm incorrectly predicts nucleus boundary or completely misses the nucleus	Place a rectangle with the correct class and color around the nucleus. The rectangle must be tight (i.e. it should be precise, not too large or too small).	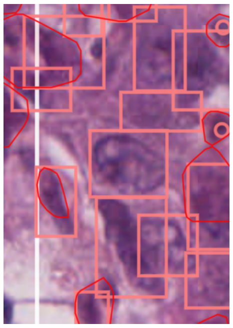
**5**	The algorithm clumps multiple nuclei together	Place a rectangle around each nucleus and ignore the algorithmic suggestion.	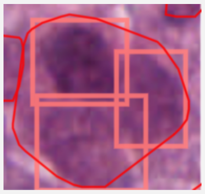
**6a**	A nucleus extends beyond the edge of the FOV and I need to place a <u>dot</u>	Place the dot inside the FOV.	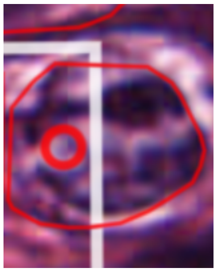
**6b**	A nucleus extends beyond the edge of the FOV and I need to place a <u>rectangle</u>	Extend your rectangle to encompass the full extend of the nucleus	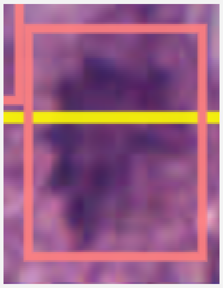
**7**	I cannot see the underlying tissue	Reduce the annotation transparency	
**8**	I know the nucleus class but it is not in the	- If you are an NP: ask on Slack; a pathologist may recommend a class.<br>- If you are a pathologist, create your own	

	standard classes	class, and notify the study coordinator.	
**9**	The algorithm predict two overlapping boundaries for the same nucleus; only one is correct	If it is possible to place the dot inside the inside the correct boundary, but outside the incorrect one, do so.	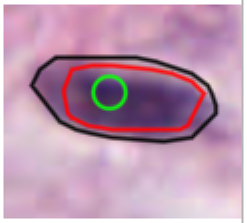
**10**	There is necrotic debris or collagen	Ignore it. Do **not** annotate debris or non-nuclear material.	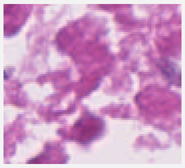
**11**	There are red blood cells	Ignore. Do **not** annotate RBCs	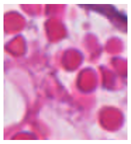
**12**	There is a multinucleated giant cell or a cell-eat-cell phenomenon (cannibalism)	Classify each nucleus independently. We operate at the level of nuclei, not cells, in this project.	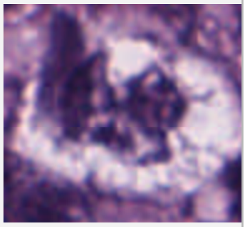
**13**	There are overlapping nuclei and the bounding boxes will have to overlap to capture full extent.	No problem; use overlapping bounding boxes in this case.	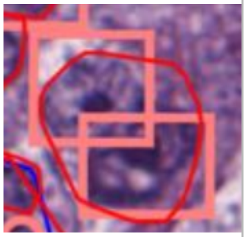
**14**	The nuclei are very textured and have prominent nucleoli	Don’t be fooled!! Malignant nuclei can have a very textured appearance and prominent nucleoli so you may think they are multiple nuclei but are one nucleus!! By the way, in the image below, there are many vacuoles that were mistaken as being nuclei. This is a vacuolated phenotype.	

		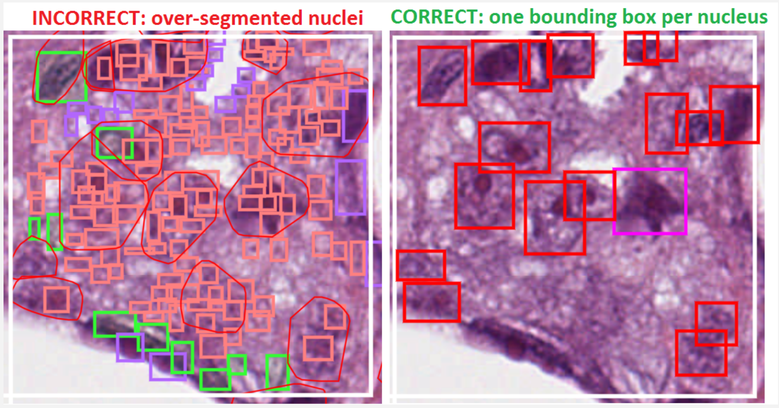	
**15**	The slide is quite difficult; stroma is difficult to distinguish from tumor.	Make sure you follow step 1 in the *Annotation workflow* section. Anything outside tumor regions may still be a tumor nucleus, but is more likely to be a fibroblast, lymphocyte, plasma cell etc.	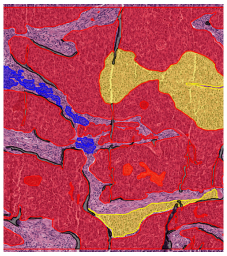
**16**	After finishing many FOVs, I discovered (or was told) that I have a systematic error in classifying nuclei (eg. all plasma cells mistakenly called tumor)	Notify one of the study coordinators and we will run a program (python script) to do this automatically for you.	
