# Supplementary material for: NuCLS: A scalable crowdsourcing approach and dataset for nucleus classification and segmentation in breast cancer
Source: Gigascience. 2022 May 17;11:giac037. doi: 10.1093/gigascience/giac037 (PMC9112766; doi:10.1093/gigascience/giac037)
Supplement: giac037_Response_to_Reviewer_Comments_Original_Submission [file giac037_response_to_reviewer_comments_original_submission.pdf]

Author's Response To Reviewer Comments

Close

** We recommend viewing the comments using the "Personal Cover" PDF file attached, which includes better formatting. Kindly also note that we attached a PDF file where additions are highlighted in yellow. It appears after the untracked file in the integrated PDF file.**

We would like to thank the editor and reviewers for their feedback and constructive suggestions, which helped improve the manuscript. We provide a point-by-point response below.

EDITORIAL COMMENTS

Reviewer #1 highlights the source code and training models should be made openly available.
Please add the following missing section that makes it clearer to readers where the source code, models and training data are:

Availability of source code and requirements [...]

This needs to be under an Open Source Initiative approved license where practicable compiled running software is made available. If the code is not hosted in a repository the GigaScience GitHub repository is also available for this purpose.

Thank you for this suggestion. This information can now be found under the section “Availability of source code and requirements” in the updated manuscript. We also added licensing information for the dataset under the “availability of supporting data and materials” section. The source code is hosted on the NuCLS Github repository (github.com/PathologyDataScience/NuCLS) and is referenced in the updated text. It is licensed under an OSI-approved MIT license. The dataset is licensed under a CC0 1.0, which is the least restrictive type, in accordance with GigaScience requirements.

In addition, please register any new software application in the bio.tools and SciCrunch.org databases to receive RRID (Research Resource Identification Initiative ID) and biotoolsID identifiers, and include these in your manuscript.

We registered the software as requested.

REVIEWER 1 COMMENTS

The goal itself is intriguing. This domain has a large number of articles. The authors should modify the title of this manuscript and it should be aligned with the main objectives.

We would like to thank the reviewer for their time and helpful feedback. We agree that this is a large domain, but we believe the current title correctly highlights the most distinctive aspects of our article, including:
- The scalable use of crowdsourcing - This aspect is novel and has not been explored in depth.
- The fact that this is a breast cancer-specific dataset. Most other works focus on other cancer types or multiple cancer types with less representation of breast cancer.
- The fact that this work includes nucleus classification, not just detection/segmentation. This is a novel aspect not widely explored in the context of breast cancer.

"[...]. This paper presents data and analysis results for single and multi-rater annotations from both non-experts and pathologists. We present a novel method for suggesting annotations that allows us to collect accurate segmentation data without the need for laborious manual tracing of cells" - How the

nucleus detection and classification method is different than the existing semi/fully automated methods? A comparative analysis with the existing approaches will be beneficial.

Our primary novelty in this paper is the workflow, not the nucleus detection/classification method used for obtaining suggestions. In a sister publication, we describe adaptations of Mask R-CNN models to work well with dense nuclei and to work with hybrid box-and-segmentation data, and to improve their explainability. Details can be found here:

Amgad M, Atteya LA, Hussein H, Mohammed KH, Hafiz E, Elsebaie MA, Mobadersany P, Manthey D, Gutman DA, Elfandy H, Cooper LA. Explainable nucleus classification using Decision Tree Approximation of Learned Embeddings. Bioinformatics. 2021.

We agree that the wording of that sentence gave the false impression that the main novelty was in the detection and classification method, and we updated the text accordingly. It now reads:

"We present a novel workflow that uses algorithmic suggestions to collect accurate segmentation data without the need for laborious manual tracing of nuclei."

Other parts of the text also clarify the fact that the focus of this manuscript and its novelty is the experimental workflow used for generating data, the rater agreement analysis methodology and result, and the dataset itself. We explain the key aspects of existing work and our novelty in the "Related work" and "Our contributions" sections. The relevant text includes:

"The approach we used involves click-based approval of annotations generated by a deep-learning algorithm. This methodological aspect is not the central focus of this paper; it is only one of many approaches for interactive segmentation and classification of nuclei explored in past studies like HistomicsML and NuClick [42, 22]."

"Finally, we note that downstream deep-learning modeling using the NuCLS dataset is discussed in a related publication and is not the focus of this paper."

How the non-experts will use this tool? Are there any software packages/web tool is available for non-experts? If yes, the authors should share the details for review, and if not, the authors should work on the development of that web tool/ software GUI. If it is associated with HistomicsUI/HistomicsTK share details for review. It will be interesting to check how the tool works on WSIs.

Thank you for the suggestion. All annotations were performed on WSI's, using the HistomicsUI interface. Our contributions to the user interface were directly incorporated into existing HistomicsUI and HistomicsTK packages. The package documentation includes all installment and usage details. We have added a new section called "Availability of source code and requirements", which states:

"Other requirements: We used this tensorflow implementation by Matterport Inc. to train the Mask R-CNN tensorflow model used for generating the algorithmic suggestions, along with a set of scripts available on Github. We used the Digital Slide Archive for WSI and data management (available here), its associated annotation user interface HistomicsUI (available here), as well as the annotation and image processing library HistomicsTK (available here)."

Additionally, other parts of the manuscript provide explanations of our contributions to the user interface and reference videos from the documentation:

"HistomicsUI. We used the Digital Slide Archive, a web-based data management tool, to assign slides and annotation tasks (digitalslidearchive.github.io) [45]. HistomicsUI, the associated annotation interface, was used for creating, correcting, and reviewing annotations. Using a centralized setup avoids participants installing software and simplifies the dissemination of images, control over view/edit permissions, monitoring progress, and collecting results. The annotation process is illustrated in this video. The process of pathologist review of annotations is illustrated in Figure S1."

"Figure S1. Use of review galleries for scalable review of single-rater annotations. Single-rater annotations were corrected by two study coordinators, in consultation with a senior pathologist. The pathologist was provided with a mosaic review gallery showing a bird's eye view of each FOV, with and

without annotations, and at high and low power. The pathologist was asked to assign a per-FOV quality assessment. If the pathologist wanted further context, they were able to click on the FOV and pan around the full whole-slide image. They were also able to provide brief comments to be addressed by the coordinators, for eg. “change all to tumor”. A demo is provided at the following video: https://youtu.be/Plh39obBg_0 .”

Mask R-CNN has been used in various articles for nucleus detection/segmentation/ classification. What are the technical novelties of the proposed approach? It will be better if the authors focus more on the novel technical contributions and statistical analysis part.

The technical novelty is not in the Mask R-CNN model; as the reviewer correctly points out, this is commonly used in the literature. Our primary novelty in this paper is the workflow, not the nucleus detection/classification method used for obtaining suggestions. In a sister publication, we describe adaptations of Mask R-CNN models to work well with dense nuclei and to work with hybrid box-and-segmentation data, and to improve explainability. Details can be found here:

Amgad M, Atteya LA, Hussein H, Mohammed KH, Hafiz E, Elsebaie MA, Mobadersany P, Manthey D, Gutman DA, Elfandy H, Cooper LA. Explainable nucleus classification using Decision Tree Approximation of Learned Embeddings. Bioinformatics. 2021.
The abstract has been modified to clarify this, and now reads:

“We present a novel workflow that uses algorithmic suggestions to collect accurate segmentation data without the need for laborious manual tracing of nuclei.”

Other parts of the text also clarify the fact that the focus of this manuscript and its novelty is the experimental workflow used for generating data, the rater agreement analysis methodology and result, and the dataset itself. We explain the key aspects of existing work and our novelty in the “Related work” and “Our contributions” sections. The relevant text includes:

“The approach we used involves click-based approval of annotations generated by a deep-learning algorithm. This methodological aspect is not the central focus of this paper; it is only one of many approaches for interactive segmentation and classification of nuclei explored in past studies like HistomicsML and NuClick [42, 22].”

“Finally, we note that downstream deep-learning modeling using the NuCLS dataset is discussed in a related publication and is not the focus of this paper [43].”

We note that the heuristic (image processing-based) methods only produce nucleus segmentation boundaries, not classifications. One of the novel aspects of our workflow is the observation that nuclei can inherit their weak classification labels from the regions in which they reside, provided that we use additional heuristics to refine the suggested classifications:

“Bootstrapping noisy training data
Region annotations were used to assign a noisy class to each segmented nucleus. This decision was based on the observation that although tissue regions usually contain multiple cell types, there is often a single predominant cell type: tumor regions / tumor cells, stromal regions / fibroblasts, lymphocytic infiltrate / lymphocytes, plasmacytic infiltrate / plasma cells, other regions / other cells. One exception to this direct mapping is stromal regions, which contain a large number of sTILs in addition to fibroblasts. Within stromal regions, a nucleus was considered a fibroblast if it had a spindle-like shape with an aspect ratio between 0.4 and 0.55 and circularity between 0.7 and 0.8.”

Also, the use of Mask R-CNN (or deep-learning in general) for function approximation to smooth noise in training data is not commonly done. We found that it resulted in improvements to the quality of algorithmic suggestions. We highlight this in the following text extracts:

“Many nucleus detection and segmentation algorithms were developed using conventional image analysis methods before the widespread adoption of CNNs. These algorithms have little or no dependence on annotations, and while they may not be as accurate as CNNs, they can correctly segment a significant fraction of nuclei. We used simple nucleus segmentation heuristics, combined with low-power region annotations from the BCSS dataset, to obtain bootstrapped annotation suggestions for nuclei (Figure S2) [28]. The suggestions were refined using a deep-learning model (Mask R-CNN) as a

function approximator trained on the bootstrapped suggestions. This procedure allowed poor quality bootstrapped suggestions in one FOV to be smoothed by better suggestions in other FOVs (Figure S4, Table S2) and is analogous to fitting a regression line to noisy data [18, 48].”

“Figure S2. Process for obtaining algorithmic suggestions for scalable assisted annotation. Nucleus segmentation boundaries were derived using image processing heuristics at a high magnification. Low-power region annotations from the BCSS dataset, approved by a practicing pathologist, were then used to assign an initial class to nuclei. This combination of noisy nuclear segmentation boundaries and region-derived classifications are the bootstrapped suggestions. These noisy algorithmic suggestions were the basis for annotating the Bootstrap control multi-rater dataset. A Mask R-CNN model was then used as a function approximator to smooth out some of the noise in the bootstrapped suggestions. Participants were able to view these refined suggestions, along with low-power region annotations, when annotating the single-rater and Evaluation datasets.”

Qualitative results need to be improved. Instead of bounding boxes, the authors should use the actual contour of cells/nuclei.

We would like to clarify the issue of segmentation versus detection here. All of the algorithmic suggestions were composed of nuclear segmentation boundaries. It is the data collection process that resulted in “hybrid” annotation data, composed of a mixture of segmentation boundaries and bounding boxes. The advantage of this approach is that we never asked the participants to delineate any nuclear boundaries, yet ~40% of our data is composed of nuclear segmentation data obtained by clicking accurate algorithmic suggestions. This is highlighted in the following text extracts:

“Accurate suggestions can be confirmed during annotation with a single click, reducing effort and providing valuable nucleus boundaries that can aid the development of segmentation models. Participants can annotate nuclei that have poor suggestions using bounding boxes. Bounding box annotation requires more effort than clicking a suggestion, but less effort than the manual tracing of nuclear boundaries [15]. We obtained a substantial proportion of nucleus boundaries through clicks: 41.7±17.3% for the Evaluation dataset and 36.6% for the single-rater dataset (Figure 4, Figure S5). The resultant hybrid dataset contained a mixture of bounding boxes and accurate segmentation boundaries (Evaluation dataset DICE=85.0±5.9). We argue that it is easier to handle hybrid datasets at the level of algorithm development than to have participants trace missing boundaries or correct imprecise ones. We evaluate the bias of using these suggestions in the following section.”

“Figure 4. Effect of algorithmic suggestions on annotation abundance and accuracy. [...]. a. Example annotations from a single participant. Algorithmic suggestions allow the collection of accurate nucleus segmentations without added effort. Yellow points indicate clicks to approve suggestions. b. The number of segmented nuclei clicked is significantly higher for the Evaluation dataset than for the Bootstrap control, indicating that refinement improves suggestion quality. c. Accuracy of algorithmic segmentation suggestions. The comparison is made against a limited set of manually traced segmentation boundaries obtained from one senior pathologist. Suggestions that were determined to be correct by the EM procedure had significantly more accurate segmentation boundaries. [...]”

“Figure S5. Abundance and segmentation accuracy of clicked algorithmic suggestions (multi-rater datasets). a. Proportion of nuclei in the FOV that were inferred to have good segmentation. Circle size represents the number of nuclei in that FOV. The proportion is notably higher for the Evaluation dataset than the Bootstrap control. b. Accuracy of algorithmic segmentation boundaries for nuclei that were inferred to have accurate segmentation boundaries in both the Evaluation dataset and Bootstrap control. The comparison is made against manual segmentations obtained for the same nuclei from one senior pathologist. Most clicked algorithmic segmentations were very accurate, and have a DICE coefficient above 0.8. The accuracy was slightly higher for Mask R-CNN-refined suggestions.”

In a related publication, we show how Mask R-CNN models can be trained with hybrid box-and-segmentation data; simply discounting bounding boxes from the mask component multi-task loss was enough. Details can be found at:

Amgad M, Atteya LA, Hussein H, Mohammed KH, Hafiz E, Elsebaie MA, Mobadersany P, Manthey D, Gutman DA, Elfandy H, Cooper LA. Explainable nucleus classification using Decision Tree Approximation of Learned Embeddings. Bioinformatics. 2021.

S10. Algorithm-The algorithm needs to be improved. It will be better if the authors share their source codes, trained model, and test data for review and reproduce the result of figure 4a on a whole slide image.

Thank you for the suggestion. This information can now be found under the section “Availability of source code and requirements” in the updated manuscript. The source code is hosted on the NuCLS Github repository (github.com/PathologyDataScience/NuCLS) and is referenced in the updated text. This repository contains the source code we developed for constrained clustering as well as the scripts we used for obtaining algorithmic suggestions. These scripts utilize as input TCGA whole-slide images, the BCSS dataset, and the HistomicsTK package, all of which are publicly available. We used HistomicsUI for whole-slide image viewing, which is also publicly available and referenced in the updated manuscript. We did not retain the trained Mask R-CNN model weights, but all the components needed to reproduce a similar model, including all code and data, are publicly available.

REVIEWER 2 COMMENTS

This important Research Article reports on an imaging-based approach for classifying breast cancer histopathology data. The study referred to in the manuscript utilises The Cancer Genome Atlas (TCGA) histology slides, one slide per breast cancer patient and 125 slides in total. Annotation is accomplished using a low-effort, semi-automated approach, which the authors refer to as "nucleus classification, localization, and segmentation" or NuCLS. This is a known computer vision problem, and the authors are to be commended for providing a software solution to help address this problem and to reduce the need for manual tracing of cells. The crowdsourcing approach referred to in this study obtained a total of 222,396 nucleus annotations, including over 125,000 single-rater annotations and 97,000 multi-rater annotations. The manuscript is well-written and [...].

We would like to thank the reviewer for their kind words and for their summary of our key contributions. We also thank the reviewer for compiling all the relevant sources of data and software. We have expanded the “Availability of supporting data and materials” section, and added a new section, “Availability of source code and requirements” to facilitate replication and access to all relevant resources.

In the Data Sources section, it states the following:
"The scanned diagnostic slides we used were generated by the TCGA Research Network [...]. They were obtained from 125 patients with breast cancer (one slide per patient). Specifically, we chose to focus on all carcinoma of unspecified type cases that were triple-negative."

Whereas I see great value in this study, I do wonder why the authors only used 125 TCGA breast cancer histopathology slides. Does this reflect a small number of unspecified (i.e. triple-negative) breast cancer histopathology slides in the TCGA? Or alternatively, is the small number of slides indicative of the crowdsourcing effort needed for the manual annotation aspect of this study?

Thank you for this question. Indeed, this number is reflective of the small number of cases in the TCGA that are triple-negative and of the unspecified type (a.k.a. Infiltrating ductal carcinoma). We note that this project is built on the BCSS project and, given the methodological design of the bootstrapping process, must use the same slides used for BCSS. We chose to focus on the infiltrating ductal subset since the majority of invasive breast cancers encountered in clinical practice are of the unspecified subtype.

REVIEWER 3 COMMENTS

The authors present a new crowdsourcing approach for nucleus segmentation and classification in pathology images. It is extremely labor-intensive work to prepare the ground truth labels for nuclei segmentation and classification due to the large number, variability in shape, and etc. The proposed work provides an alternative way of generating labels for pathology image analysis. I appreciate the authors for their intense and hard work. The results would be valuable for other related studies in digital and computational pathology.

We would like to thank the reviewer for their time, comments, and helpful feedback.
1) The authors defined and used many terms in the manuscript. Several terms are similar to each other. Even though most of them are given in the supplementary material, it is not entirely clear what each means as reading the manuscript. It makes extremely hard to follow and understand the content of the manuscript.

Thank you for this comment. We have done the following to improve clarity:

We included relevant abbreviations from Table S1 that were missing in the "List of Abbreviations" section.
We expanded out some abbreviations throughout the text to reduce mental effort in reading. Terms like CNN, WSI, H&E, and API have been used in full throughout. The only abbreviations left are those that describe accuracy metrics (AUROC, MCC, AP) and those that are central to the workflow we describe and are repeated throughout the text, such as P-truth and NP-label.
We made sure all key terms are expanded and defined at first occurrence.

2) This work is about nucleus classification and segmentation dataset. But, nucleus segmentation has not been that well studied. Only one experiment between a pathologist and an algorithm is given in the manuscript. The platform per se seems to be better suited for nucleus detection and classification, not segmentation. Hence, the authors may focus on nucleus detection and classification only.

We would like to clarify the issue of segmentation versus detection here. All of the algorithmic suggestions were composed of nuclear segmentation boundaries. It is the data collection process that resulted in "hybrid" annotation data, composed of a mixture of segmentation boundaries and bounding boxes. The advantage of this approach is that we never asked the participants to delineate any nuclear boundaries, yet ~40% of our data is composed of nuclear segmentation data obtained by clicking accurate algorithmic suggestions. This is highlighted in the following text extracts:

"Accurate suggestions can be confirmed during annotation with a single click, reducing effort and providing valuable nucleus boundaries that can aid the development of segmentation models. Participants can annotate nuclei that have poor suggestions using bounding boxes. Bounding box annotation requires more effort than clicking a suggestion, but less effort than the manual tracing of nuclear boundaries [15]. We obtained a substantial proportion of nucleus boundaries through clicks: 41.7±17.3% for the Evaluation dataset and 36.6% for the single-rater dataset (Figure 4, Figure S5). The resultant hybrid dataset contained a mixture of bounding boxes and accurate segmentation boundaries (Evaluation dataset DICE=85.0±5.9). We argue that it is easier to handle hybrid datasets at the level of algorithm development than to have participants trace missing boundaries or correct imprecise ones. We evaluate the bias of using these suggestions in the following section."

"Figure 4. Effect of algorithmic suggestions on annotation abundance and accuracy. [...]. a. Example annotations from a single participant. Algorithmic suggestions allow the collection of accurate nucleus segmentations without added effort. Yellow points indicate clicks to approve suggestions. b. The number of segmented nuclei clicked is significantly higher for the Evaluation dataset than for the Bootstrap control, indicating that refinement improves suggestion quality. c. Accuracy of algorithmic segmentation suggestions. The comparison is made against a limited set of manually traced segmentation boundaries obtained from one senior pathologist. Suggestions that were determined to be correct by the EM procedure had significantly more accurate segmentation boundaries. [...]"

"Figure S5. Abundance and segmentation accuracy of clicked algorithmic suggestions (multi-rater datasets). a. Proportion of nuclei in the FOV that were inferred to have good segmentation. Circle size represents the number of nuclei in that FOV. The proportion is notably higher for the Evaluation dataset than the Bootstrap control. b. Accuracy of algorithmic segmentation boundaries for nuclei that were inferred to have accurate segmentation boundaries in both the Evaluation dataset and Bootstrap control. The comparison is made against manual segmentations obtained for the same nuclei from one senior pathologist. Most clicked algorithmic segmentations were very accurate, and have a DICE coefficient above 0.8. The accuracy was slightly higher for Mask R-CNN-refined suggestions."

In a related publication, we show how Mask R-CNN models can be trained with hybrid box-and-

segmentation data; simply discounting bounding boxes from the mask component multi-task loss was enough. Details can be found at:

Amgad M, Atteya LA, Hussein H, Mohammed KH, Hafiz E, Elsebaie MA, Mobadersany P, Manthey D, Gutman DA, Elfandy H, Cooper LA. Explainable nucleus classification using Decision Tree Approximation of Learned Embeddings. Bioinformatics. 2021.

3) In page 4, "Many nucleus detection and segmentation algorithms were developed using conventional image analysis methods before the widespread adoption of CNNs. These algorithms have little or no dependence on annotations, and while they may not be as accurate as CNNs, they can correctly segment a significant fraction of nuclei.". Perhaps, from the perspective of nucleus detection, this statement is correct. However, in regard with nucleus segmentation, in particular separating touching nuclei, this is no valid, to my understanding. CNN-based methods have already shown its superiority in several literature. Also, the results show that an accurate suggestion by an algorithm could improve the annotations by NPs. So, there is a potential for CNN-based methods could further contribute to the crowdsourding datasets.

Thank you for this comment. We agree, and we've updated the limitations section to highlight this fact, as follows:

"Another limitation is that the initial bootstrapped nuclear boundaries were generated using classical image processing methods, which tend to underperform where nuclei are highly clumped/touching or have very faint staining. This theoretically introduces some bias in our dataset, with an overrepresentation of simpler nuclear boundaries. Future work could investigate the use of transfer learning or unsupervised CNN approaches to generate more accurate algorithmic suggestions."

4) In page 6, FOV sampling procedure was done by pathologists?

ROIs were selected by a medical doctor and approved by a practicing pathologist. These ROIs were then computationally sampled to get small FOVs using prespecified quantitative criteria. We have updated the text to address this question:

"ROI locations were carried over from the BCSS dataset. ROIs were manually selected by a medical doctor (M.A.), who served as a study coordinator for both the BCSS and NuCLS projects, and approved by a senior pathologist (H.E.). These ROIs were then tiled into non-overlapping potential FOVs, which were automatically selected for inclusion in our study based on predefined stratified sampling criteria."

Close
