# Supplementary material for: NuCLS: A scalable crowdsourcing approach and dataset for nucleus classification and segmentation in breast cancer
Source: Gigascience. 2022 May 17;11:giac037. doi: 10.1093/gigascience/giac037 (PMC9112766; doi:10.1093/gigascience/giac037)
Supplement: giac037_Response_to_Reviewer_Comments_Revision_1 [file giac037_response_to_reviewer_comments_revision_1.pdf]

Author's Response To Reviewer Comments

Close

*** IMPORTANT NOTE: Kindly view the PDF version attached for a better formatted version of the reviewer response. Thank you! ***

We would like to thank the editor and reviewers for their support and helpful comments and suggestions. Below is a point-by-point response to the comments raised.

EDITORIAL COMMENTS

Your manuscript "NuCLS: A scalable crowdsourcing approach & dataset for nucleus classification and segmentation in breast cancer" (GIGA-D-21-00352R1) has been assessed by our reviewers. Based on these reports, and my own assessment as Editor, I am pleased to inform you that it is potentially acceptable for publication in GigaScience, once you have carried out some essential revisions suggested by our reviewers.

Reviewer #1 feels that claims on "novelty" is a bit too strong, so we suggest to tone down the novelty aspect or provide evidence to support the claims. Also an improvement in code documentation in the GitHub will be required for reproducibility and reuse.

We would like to thank the editor for their comment and for conditional acceptance of the work. We would like to point out that the revised manuscript only claims novelty with regards to data and workflow, and makes no mentions of contributions to deep-learning methodology, which is not the aim or focus of the paper. The only sentences where claims of novelty are used are included below:

“This paper describes a novel collaborative framework for engaging crowds of medical students and pathologists to produce quality labels for cell nuclei.”

“We present a novel workflow that uses algorithmic suggestions to collect accurate segmentation data without the need for laborious manual tracing of nuclei.”

“In addition, we discuss a new constrained clustering method that we developed for reliable truth inference in multi-rater datasets.” and “In addition, we discuss a new constrained clustering method that we developed for reliable truth inference in multi-rater datasets.”

In each of these instances, the claim is limited to the data collection method, the datasets, and truth inference. We do not make any claims about deep-learning novelty, since this is not the focus or intent of this paper. This is a paper about a new dataset, data collection methodology, and exploration of rater agreement at various levels of expertise in computational pathology.

Also an improvement in code documentation in the GitHub will be required for reproducibility and reuse.

Thank you for the suggestion. We have expanded the documentation of the Github repository accordingly.

REVIEWER 1 COMMENTS

The authors previously claimed that their methodology is novel. After revision, they claimed that their workflow is novel. The statement is confusing. The authors should provide sufficient evidence in support of their claim.

Please allow us to clarify this point. While the first version we submitted to the journal had some statements about novelty in deep-learning algorithms. These statements were removed from the revised submission. As we explained in the editorial response above, there are only four sentences in the manuscript that make claims of novelty, and they are entirely focused on the dataset, the data collection methodology, and the truth inference method.

The authors didn't reply to my question related to the comparative analysis. It will be better if the authors compare the performance of their workflow by replacing Mask R-CNN with other deep neural networks.

We would like to clarify the role of Mask R-CNN in our paper. Mask R-CNN was used only to generate the suggestions shown to participants. The participants then used these suggestions to generate data in a study that lasted over 1 year. Generating suggestions was the very first step in our analysis and Mask R-CNN was deliberately chosen as the state-of-the-art at the time. It is not feasible to evaluate alternatives to Mask R-CNN due to the time it takes reviewers to generate annotations. We have updated the conclusions section to direct future research to explore other architectures as follows, although we do not believe this is a significant factor in the bigger picture of our approach:

“Similarly, we used Mask R-CNN as a function approximator to refine our algorithmic suggestions. Future research can explore other deep-learning architectures that may improve refinement and result in better algorithmic suggestions.”

I checked the Github repository four years old code written by someone else. I found a Github link. Most probably, this is the actual source of the Mask_RCNN code.

This is already mentioned in the manuscript. Under the section “Availability of source code and requirements,” we state:

“Other requirements: We used this TensorFlow implementation by Matterport Inc. to train the Mask R-CNN tensorflow model used for generating the algorithmic suggestions, along with a set of scripts available on Github.”

The authors' codes contain a lack of instructions.

We have expanded the documentation of the Github repository accordingly.

Close
