# Supplementary material for: NuCLS: A scalable crowdsourcing approach and dataset for nucleus classification and segmentation in breast cancer
Source: Gigascience. 2022 May 17;11:giac037. doi: 10.1093/gigascience/giac037 (PMC9112766; doi:10.1093/gigascience/giac037)
Supplement: giac037_Response_to_Reviewer_Comments_Revision_2 [file giac037_response_to_reviewer_comments_revision_2.pdf]

Author's Response To Reviewer Comments

Close

We would like to thank the editor and reviewers for their support throughout this process. We have cited the GigaDB dataset as requested and submit the updated manuscript. We would also like to thank the GigaDB database curation team for their time and diligence.

Close
