# Supplementary material for: NuCLS: A scalable crowdsourcing approach and dataset for nucleus classification and segmentation in breast cancer
Source: Gigascience. 2022 May 17;11:giac037. doi: 10.1093/gigascience/giac037 (PMC9112766; doi:10.1093/gigascience/giac037)
Supplement: giac037_Reviewer_1_Report_Original_Submission — Monjoy Saha, PhD -- 11/21/2021 Reviewed [file giac037_reviewer_1_report_original_submission.pdf]

Reviewer Report

Title: NuCLS: A scalable crowdsourcing approach & dataset for nucleus classification and segmentation in breast cancer

Version: Original Submission **Date:** 11/21/2021

Reviewer name: Monjoy Saha, PhD

Reviewer Comments to Author:

The manuscript is about breast cancer nucleus classification and segmentation. The goal itself is intriguing. This domain has a large number of articles. The authors should modify the title of this manuscript and it should be aligned with the main objectives. I have the following queries:
"This paper presents data and analysis results for single and multi-rater annotations from both non-experts and pathologists. We present a novel method for suggesting annotations that allows us to collect accurate segmentation data without the need for laborious manual tracing of cells"- How the nucleus detection and classification method is different than the existing semi/fully automated methods? A comparative analysis with the existing approaches will be beneficial.
"Our results indicate that even noisy algorithmic suggestions do not adversely affect pathologist accuracy, and can help non-experts improve annotation quality"- How the non-experts will use this tool? Are there any software packages/web tool is available for non-experts? If yes, the authors should share the details for review, and if not, the authors should work on the development of that web tool/ software GUI. If it is associated with HistomicsUI/HistomicsTK share details for review. It will be interesting to check how the tool works on WSIs.
Mask R-CNN has been used in various articles for nucleus detection/segmentation/classification. What are the technical novelties of the proposed approach?
It will be better if the authors focus more on the novel technical contributions and statistical analysis part. Qualitative results need to be improved. Instead of bounding boxes, the authors should use the actual contour of cells/nuclei.
S10. Algorithm-The algorithm needs to be improved. It will be better if the authors share their source codes, trained model, and test data for review and reproduce the result of figure 4a on a whole slide image.

Methods

Are the methods appropriate to the aims of the study, are they well described, and are necessary controls included? Choose an item.

Conclusions

Are the conclusions adequately supported by the data shown? Choose an item.

Reporting Standards

Does the manuscript adhere to the journal's guidelines on [minimum standards of reporting?] Choose an item.

Choose an item.

Statistics

Are you able to assess all statistics in the manuscript, including the appropriateness of statistical tests used? Choose an item.

Quality of Written English

Please indicate the quality of language in the manuscript: Choose an item.

Declaration of Competing Interests

Please complete a declaration of competing interests, considering the following questions:

- Have you in the past five years received reimbursements, fees, funding, or salary from an organisation that may in any way gain or lose financially from the publication of this manuscript, either now or in the future?
- Do you hold any stocks or shares in an organisation that may in any way gain or lose financially from the publication of this manuscript, either now or in the future?
- Do you hold or are you currently applying for any patents relating to the content of the manuscript?
- Have you received reimbursements, fees, funding, or salary from an organization that holds or has applied for patents relating to the content of the manuscript?
- Do you have any other financial competing interests?
- Do you have any non-financial competing interests in relation to this paper?

If you can answer no to all of the above, write 'I declare that I have no competing interests' below. If your reply is yes to any, please give details below.

Some of the authors who contributed to this paper are people I know. Furthermore, I was affiliated with an organization that includes certain authors.

I agree to the open peer review policy of the journal. I understand that my name will be included on my report to the authors and, if the manuscript is accepted for publication, my named report including any attachments I upload will be posted on the website along with the authors' responses. I agree for my report to be made available under an Open Access Creative Commons CC-BY license (http://creativecommons.org/licenses/by/4.0/). I understand that any comments which I do not wish to be included in my named report can be included as confidential comments to the editors, which will not be published.

Choose an item.

To further support our reviewers, we have joined with Publons, where you can gain additional credit to further highlight your hard work (see: https://publons.com/journal/530/gigascience). On publication of this paper, your review will be automatically added to Publons, you can then choose whether or not to claim your Publons credit. I understand this statement.

Yes Choose an item.
