# Supplementary material for: NuCLS: A scalable crowdsourcing approach and dataset for nucleus classification and segmentation in breast cancer
Source: Gigascience. 2022 May 17;11:giac037. doi: 10.1093/gigascience/giac037 (PMC9112766; doi:10.1093/gigascience/giac037)
Supplement: giac037_Reviewer_2_Report_Original_Submission — Chris Armit -- 11/24/2021 Reviewed [file giac037_reviewer_2_report_original_submission.pdf]

Reviewer Report

Title: NuCLS: A scalable crowdsourcing approach & dataset for nucleus classification and segmentation in breast cancer

Version: Original Submission **Date:** 11/24/2021

Reviewer name: Chris Armit

Reviewer Comments to Author:

This important Research Article reports on an imaging-based approach for classifying breast cancer histopathology data. The study referred to in the manuscript utilises The Cancer Genome Atlas (TCGA) histology slides, one slide per breast cancer patient and 125 slides in total. Annotation is accomplished using a low-effort, semi-automated approach, which the authors refer to as "nucleus classification, localization, and segmentation" or NuCLS. This is a known computer vision problem, and the authors are to be commended for providing a software solution to help address this problem and to reduce the need for manual tracing of cells. The crowdsourcing approach referred to in this study obtained a total of 222,396 nucleus annotations, including over 125,000 single-rater annotations and 97,000 multi-rater annotations. The manuscript is well-written and the NuCLS image data and annotations can be accessed from the following Google link: https://sites.google.com/view/nucls/home
In addition, the Breast Cancer Semantic Segmentation (BCSS) dataset that helped contribute to the algorithmic suggestions referred to in the manuscript is accessible from the Digital Slide Archive and can be found at the following link: https://digitalslidearchive.github.io/digital_slide_archive/
The HistomicsUI software tool is available from the following GitHub repository where it has been ascribed an OSI-approved Apache-2.0 License: https://github.com/DigitalSlideArchive/HistomicsUI
In addition, the algorithmic suggestions referred to in the manuscript - which the authors show can improve classification accuracy - are publicly available from the following GitHub repository that has an OSI-approved MIT License: https://github.com/PathologyDataScience/NuCLS
Furthermore, the HistomicsTK Python package that was used to upload algorithmic suggestions is made publicly available from an additional GitHub repository that has an OSI-approved Apache-2.0 License: https://github.com/DigitalSlideArchive/HistomicsTK
Minor comment
In the Data Sources section, it states the following:
"The scanned diagnostic slides we used were generated by the TCGA Research Network (https://www.cancer.gov/tcga). They were obtained from 125 patients with breast cancer (one slide per patient). Specifically, we chose to focus on all carcinoma of unspecified type cases that were triple-negative."
Whereas I see great value in this study, I do wonder why the authors only used 125 TCGA breast cancer histopathology slides. Does this reflect a small number of unspecified (i.e. triple-negative) breast cancer histopathology slides in the TCGA? Or alternatively is the small number of slides indicative of the crowdsourcing effort needed for the manual annotation aspect of this study?

Methods

Are the methods appropriate to the aims of the study, are they well described, and are necessary controls included? Choose an item.

Conclusions

Are the conclusions adequately supported by the data shown? Choose an item.

Reporting Standards

Does the manuscript adhere to the journal's guidelines on minimum standards of reporting? Choose an item.

Choose an item.

Statistics

Are you able to assess all statistics in the manuscript, including the appropriateness of statistical tests used? Choose an item.

Quality of Written English

Please indicate the quality of language in the manuscript: Choose an item.

Declaration of Competing Interests

Please complete a declaration of competing interests, considering the following questions:

- Have you in the past five years received reimbursements, fees, funding, or salary from an organisation that may in any way gain or lose financially from the publication of this manuscript, either now or in the future?
- Do you hold any stocks or shares in an organisation that may in any way gain or lose financially from the publication of this manuscript, either now or in the future?
- Do you hold or are you currently applying for any patents relating to the content of the manuscript?
- Have you received reimbursements, fees, funding, or salary from an organization that holds or has applied for patents relating to the content of the manuscript?
- Do you have any other financial competing interests?
- Do you have any non-financial competing interests in relation to this paper?

If you can answer no to all of the above, write 'I declare that I have no competing interests' below. If your reply is yes to any, please give details below.

I work for GigaScience where I perform checks on the quality of image data and the image analysis approach. My funding is not dependent on the outcome of this review, and I declare that I have no competing interests.

I agree to the open peer review policy of the journal. I understand that my name will be included on my report to the authors and, if the manuscript is accepted for publication, my named report including any attachments I upload will be posted on the website along with the authors' responses. I agree for my report to be made available under an Open Access Creative Commons CC-BY license (http://creativecommons.org/licenses/by/4.0/). I understand that any comments which I do not wish to be included in my named report can be included as confidential comments to the editors, which will not be published.

Choose an item.

To further support our reviewers, we have joined with Publons, where you can gain additional credit to further highlight your hard work (see: https://publons.com/journal/530/gigascience). On publication of this paper, your review will be automatically added to Publons, you can then choose whether or not to claim your Publons credit. I understand this statement.

Yes Choose an item.
