# Supplementary material for: NuCLS: A scalable crowdsourcing approach and dataset for nucleus classification and segmentation in breast cancer
Source: Gigascience. 2022 May 17;11:giac037. doi: 10.1093/gigascience/giac037 (PMC9112766; doi:10.1093/gigascience/giac037)
Supplement: giac037_Reviewer_3_Report_Original_Submission — Jin Tae Kwak -- 12/7/2021 Reviewed [file giac037_reviewer_3_report_original_submission.pdf]

Reviewer Report

Title: NuCLS: A scalable crowdsourcing approach & dataset for nucleus classification and segmentation in breast cancer

Version: Original Submission **Date:** 12/7/2021

Reviewer name: Jin Tae Kwak

Reviewer Comments to Author:

The authors present a new crowdsourcing approach for nucleus segmentation and classification in pathology images. It is extremely labor-intensive work to prepare the ground truth labels for nuclei segmentation and classification due to the large number, variability in shape, and etc. The proposed work provides an alternative way of generating labels for pathology image analysis. I appreciate the authors for their intense and hard work. The results would be valuable for other related studies in digital and computational pathology. However, I have some concerns as follows:
1) The authors defined and used many terms in the manuscript. Several terms are similar to each other. Even though most of them are given in the supplementary material, it is not entirely clear what each means as reading the manuscript. It makes extremely hard to follow and understand the content of the manuscript.
2) This work is about nucleus classification and segmentation dataset. But, nucleus segmentation has not been that well studied. Only one experiment between a pathologist and an algorithm is given in the manuscript. The platform per se seems to be better suited for nucleus detection and classification, not segmentation. Hence, the authors may focus on nucleus detection and classification only.
3) In page 4, "Many nucleus detection and segmentation algorithms were developed using conventional image analysis methods before the widespread adoption of CNNs. These algorithms have little or no dependence on annotations, and while they may not be as accurate as CNNs, they can correctly segment a significant fraction of nuclei.". Perhaps, from the perspective of nucleus detection, this statement is correct. However, in regard with nucleus segmentation, in particular separating touching nuclei, this is no valid, to my understanding. CNN-based methods have already shown its superiority in several literature. Also, the results show that an accurate suggestion by an algorithm could improve the annotations by NPs. So, there is a potential for CNN-based methods could further contribute to the crowdsourding datasets.
4) In page 6, FOV sampling procedure was done by pathologists?

Methods

Are the methods appropriate to the aims of the study, are they well described, and are necessary controls included? Choose an item.

Conclusions

Are the conclusions adequately supported by the data shown? Choose an item.

Reporting Standards

Does the manuscript adhere to the journal's guidelines on [minimum standards of reporting?]() Choose an item.

Choose an item.

Statistics

Are you able to assess all statistics in the manuscript, including the appropriateness of statistical tests used? Choose an item.

Quality of Written English

Please indicate the quality of language in the manuscript: Choose an item.

Declaration of Competing Interests

Please complete a declaration of competing interests, considering the following questions:

- Have you in the past five years received reimbursements, fees, funding, or salary from an organisation that may in any way gain or lose financially from the publication of this manuscript, either now or in the future?
- Do you hold any stocks or shares in an organisation that may in any way gain or lose financially from the publication of this manuscript, either now or in the future?
- Do you hold or are you currently applying for any patents relating to the content of the manuscript?
- Have you received reimbursements, fees, funding, or salary from an organization that holds or has applied for patents relating to the content of the manuscript?
- Do you have any other financial competing interests?
- Do you have any non-financial competing interests in relation to this paper?

If you can answer no to all of the above, write 'I declare that I have no competing interests' below. If your reply is yes to any, please give details below.

I declare that I have no competing interests

I agree to the open peer review policy of the journal. I understand that my name will be included on my report to the authors and, if the manuscript is accepted for publication, my named report including any attachments I upload will be posted on the website along with the authors' responses. I agree for my report to be made available under an Open Access Creative Commons CC-BY license (http://creativecommons.org/licenses/by/4.0/). I understand that any comments which I do not wish to be included in my named report can be included as confidential comments to the editors, which will not be published.

Choose an item.

To further support our reviewers, we have joined with Publons, where you can gain additional credit to further highlight your hard work (see: https://publons.com/journal/530/gigascience). On publication of this paper, your review will be automatically added to Publons, you can then choose whether or not to claim your Publons credit. I understand this statement.

Yes Choose an item.
